# *Salmonella* Modulation of Host Cell Gene Expression Promotes Its Intracellular Growth

**DOI:** 10.1371/journal.ppat.1003668

**Published:** 2013-10-03

**Authors:** Sebastian Hannemann, Beile Gao, Jorge E. Galán

**Affiliations:** Department of Microbial Pathogenesis, Yale University School of Medicine, New Haven, Connecticut, United States of America; Duke University, United States of America

## Abstract

*Salmonella* Typhimurium has evolved a complex functional interface with its host cell largely determined by two type III secretion systems (T3SS), which through the delivery of bacterial effector proteins modulate a variety of cellular processes. We show here that *Salmonella* Typhimurium infection of epithelial cells results in a profound transcriptional reprogramming that changes over time. This response is triggered by *Salmonella* T3SS effector proteins, which stimulate unique signal transduction pathways leading to STAT3 activation. We found that the *Salmonella*-stimulated changes in host cell gene expression are required for the formation of its specialized vesicular compartment that is permissive for its intracellular replication. This study uncovers a cell-autonomous process required for *Salmonella* pathogenesis potentially opening up new avenues for the development of anti-infective strategies that target relevant host pathways.

## Author Summary

Essential for the ability of *Salmonella* Typhimurium to cause disease is the function of a type III secretion system (T3SS) encoded within its pathogenicity island 1 (SPI-1), which through the delivery of bacterial effector proteins modulates a variety of cellular functions. This study reports that the infection of mammalian cells with *Salmonella* Typhimurium results in a profound reprogramming of gene expression that changes over time. The stimulation of this response requires the activity of a specific subset of bacterial T3SS effector proteins, which stimulate unique signal transduction pathways leading to STAT3 activation. We found that the *Salmonella*-stimulated changes in host cell gene expression are required for its intracellular replication. Targeting the mechanisms described in this study may lead to the development of novel anti-infective strategies.

## Introduction

Bacterial pathogens that have sustained long-standing associations with their hosts have developed complex functional interfaces shaped by the concerted activities of molecules from the host and the pathogen [1]–[4]. For many pathogens, this functional interface is largely dependent on the activity of specialized protein secretion machines known as type III secretion systems (T3SSs), which deliver bacterial effector proteins into host cells to modulate a variety of cellular processes [1], [5]. One example of such a pathogen is *Salmonella enterica* serovar Typhimurium (*S.* Typhimurium), a cause of human gastroenteritis, which interacts with host cells through the activities of two T3SSs encoded within its pathogenicity islands 1 (SPI-1) and 2 (SPI-2) [6]–[8]. The SPI-1 T3SS mediates bacterial entry into non-phagocytic epithelial cells, while the SPI-2 T3SS is required for the building and maintenance of a specialized membranous compartment that harbors the intracellular bacteria. Bacterial internalization is mediated by the SPI-1 T3SS effectors SopE, SopE2, and SopB, which activate the Rho family of GTPases Rac1, Cdc42 and RhoG [9], [10]. In addition these bacterial effectors stimulate a transcriptional reprogramming in host cells, which leads to the production of pro-inflammatory cytokines believed to be essential for the initiation of the inflammatory diarrhea that characterizes acute *Salmonella* infection [11], [12]. The early transcriptional responses stimulated by *Salmonella* upon infection of intestinal epithelial cells exhibit many of the hallmarks of the responses seen after the stimulation of innate immune receptors [13]. However, the *Salmonella*-induced responses are unique in that this pathogen is capable of stimulating them independently of innate immune receptors [12], which are largely inactive in intestinal epithelial cells due to robust negative regulatory mechanisms [14]–[16]. Indeed, inflammation is essential for *Salmonella* growth in the intestine since without inflammation this pathogen cannot gain access to essential nutrients [17] and cannot effectively compete with the normal microbial flora [18]. Therefore, despite exhibiting the fingerprints of an innate immune response, the early transcriptional responses stimulated by *Salmonella* can be best characterized as a pathogen-driven process triggered by specific adaptations to cope with the host environment rather than as a hard-wired host defense response to conserved bacterial products.

Although there is ample evidence for a role of this pathogen-induced transcriptional reprogramming of epithelial cells in the initiation of the inflammatory response to *Salmonella* through the production of pro-inflammatory cytokines [19], [20], it is unknown whether these changes in gene expression influence other aspects of *Salmonella* biology. In fact, despite the widely demonstrated ability of many pathogens to stimulate transcriptional responses in infected cells [13], there is surprisingly little evidence for a potential influence of these responses in cell autonomous processes that may affect intracellular pathogen biology. Here we have characterized the transcriptional responses of cultured epithelial cells stimulated by *S.* Typhimurium during its intracellular stage and have dissected the signaling pathways that lead to these responses. Our results demonstrate an important role for the pathogen-induced changes in host-cell gene expression in the establishment of an intracellular niche suitable for *Salmonella* replication. This study uncovers a previously unknown strategy utilized by an intracellular bacterial pathogen to promote its replication within host cells, which represents a remarkable example of pathogen modulation of host responses for its own benefit.

## Results

### *Salmonella* Typhimurium triggers a complex gene expression program in cultured epithelial cells

**Figure 1:**
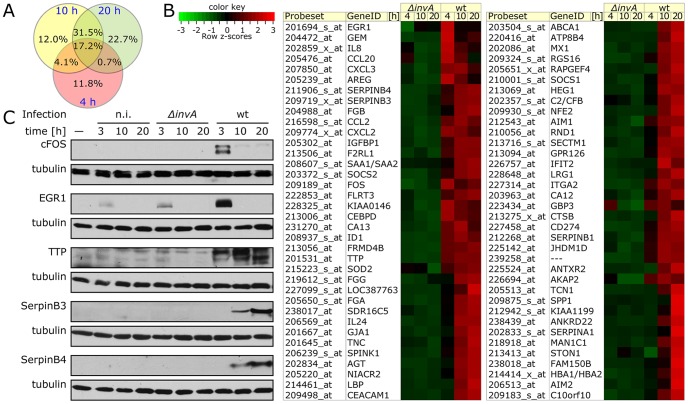
*Salmonella* Typhimurium triggers a complex gene expression program in cultured Henle-407 cells. (**A**) Venn diagram depicting the number of unique and common genes whose expression changed at least 3 fold at the indicated times after *S.* Typhimurium infection. (**B**) Heat map of selected genes whose expression changed at least 12 fold at the indicated times after *S.* Typhimurium infection. (**C**) Western blot detection of selected proteins whose genes were significantly upregulated after *S.* Typhimurium infection. Henle-407 cells were infected (MOI = 10) with wild-type *S.* Typhimurium or the SPI-1 T3SS-defective *ΔinvA* mutant strain for 1 h. At the indicated times, cells were harvested, their lysates separated by SDS-PAGE and probed by immuno blotting with the specified antibodies to the indicated proteins of interest and to tubulin as a loading control. (n. i.: non infected).

We have previously shown that cultured epithelial cells undergo a significant transcriptional reprogramming shortly after infection with *S.* Typhimurium that is strictly dependent on the function of its SPI-1 T3SS [11], [12]. In the present study, we have examined the transcriptional profile of epithelial cells at a much later time point following infection with wild-type *S.* Typhimurium. We found that the majority of the genes whose expression increased 10 h after infection were not upregulated early in infection (Fig. 1A, 1B and Table S1). Twenty hours after infection, the transcriptional response was even more distinct, and only ∼25% of the upregulated genes at this time point were also upregulated early in infection (Fig. 1A, 1B and Table S1). Interestingly, while genes associated with transcriptional innate immune responses dominated the initial response to bacterial infection (Table S1) [12], later in infection genes associated with other biological processes were more prevalent. For example, genes associated with cell adhesion, G protein signaling, lipid metabolism, vesicle traffic, protease inhibition, and processes associated with the general maintenance of cell homeostasis were uniquely induced later in infection. These results indicate that later in infection there is a distinct transcriptional program that results in a pattern of gene expression that is very different from that observed early in infection. However, like the early transcriptional responses, stimulation of the responses later in infection was also strictly dependent on the SPI-1 T3SS (Fig. 1B and Table S1). This observation suggests that the unique features of the late transcriptional responses to *Salmonella* may be simply the result of the intrinsic dynamics of the responses triggered early in infection. In fact, early in infection *S.* Typhimurium induced the expression of a number of transcription factors (e. g. FOS, FOSB, FOSL1, JUN, JUNB, EGR1, EGR4, ATF3, STAT3) (Table S1) [12], which could significantly modify and amplify the transcriptional responses later in infection. Alternatively, this pathogen may utilize specific mechanisms to further modulate host responses later in infection. To discriminate between these two possibilities we examined the transcriptional responses of cells infected with a *S.* Typhimurium *Δasd* mutant [21]. Although its levels of infection are indistinguishable from those of wild-type *S.* Typhimurium (Fig. S1) [21], the *S.* Typhimurium *Δasd* mutant is only able to survive and grow in the presence of L-diaminopimelic acid, an essential component of the cell wall that is absent in mammalian cells. Therefore, shortly after infection, the SPI-1 T3SS of the *S.* Typhimurium *Δasd* mutant becomes non-functional (Fig. S1) and the bacteria mutant dies shortly after entering into mammalian cells (Fig. S1) [21]. Cells infected with the *Δasd* mutant strain showed a gene expression profile that overlapped significantly with that observed in cells infected with wild type, particularly when considering genes whose expression as a consequence of infection changed the most (Fig. S2 and Table S1). These observations suggest that the pattern of gene expression observed in infected cells at later times after infection is defined in large measure by responses triggered early in infection.

We examined in infected cells the levels of a subset of proteins (EGR1, c-FOS, TTP, SerpinB3 and Serpin B4) whose genes were most significantly upregulated after *S.* Typhimurium infection. Consistent with the transcriptional response, we found much higher levels of these proteins in infected cells (Fig. 1C). However, we observed very significant kinetic differences in their expression. For example, although the levels of EGR1 and c-FOS were markedly increased shortly after infection, 10 h after infection these proteins were not detectable in lysates of *Salmonella* infected cells (Fig. 1C). In contrast, expression of TTP was observed throughout bacterial infection, while SerpinB3 and SerpinB4 were not detected until 10 h after infection (Fig. 1C), despite the fact that their mRNA levels were drastically increased shortly after *Salmonella* infection (Table S1). In all cases, and as predicted from the mRNA measurements, stimulation of expression of these proteins was strictly dependent on the presence of a functional SPI-1 T3SS (Fig. 1C). These results indicated that at least for a subset of genes there are post-trancriptional regulatory mechanisms that further modulate the gene expression changes stimulated by *Salmonella*. Taken together, these results revealed a complex host cell response to *Salmonella* infection leading to profound changes in gene expression

### *Salmonella* Typhimurium stimulation of transcriptional responses in infected cells requires STAT3

**Figure 2:**
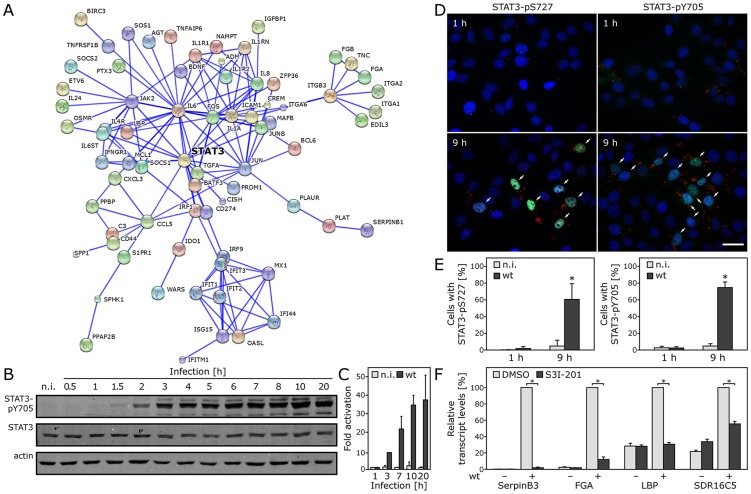
*Salmonella* stimulation of transcriptional responses in infected cells requires STAT3. (**A**) Interaction map of genes whose expression is stimulated by *S.* Typhimurium infection of Henle-407 cells. Shown is the interaction of genes whose expression increased at least 3 fold at 10 or 20 h after infection. The analysis was carried out with STRING 9.0 (http://string-db.org/) using the highest confidence (0.9) parameters. (**B–E**) *S.* Typhimurium induces STAT3 activation. Henle-407 cells were infected (MOI = 10) with wild-type *S.* Typhimurium for 1 h. Following chase in gentamicin containing medium, cells were lysed at the indicated times, separated by SDS-PAGE and probed by immuno blotting with antibodies to the phosphorylated (activated) form of STAT3 (P-Y705), unphosphorylated STAT3 (to detect total amount), and actin (loading control) (**B**). Fold activation of STAT3 (relative to uninfected cells) was quantified by scanning the blots with a LI-COR Odyssey imaging system standardizing the signals with the actin loading control. Values are the means (± SD) of three independent experiments (**C**). Alternatively, Henle-407 cells were infected (MOI = 5) for 1 h with wild-type *S.* Typhimurium, chased for 8 h in gentamicin supplemented medium, fixed, immuno stained for LPS (red), DNA (blue) and phosphorylated STAT3 (green), as indicated. Samples were then analyzed by epifluorescence microscopy (bar represents 10 µm) (**D**). The number of infected cells showing staining of phosphorylated STAT3 was determined and the values represent the mean (± SD) of three independent experiments in which at least 100 cells were quantified. *: indicates values that are statistically significantly different from uninfected controls (*p*≤0.01) (**E**). (**F**) STAT3 is required for the transcriptional responses to *S.* Typhimurium infection. Henle-407 cells treated with the STAT3 inhibitor S31-201 were infected (MOI = 10) with *S.* Typhimurium for 1 h, and chased for additional 3 h in gentamicin containing medium in the presence of DMSO or 100 µM S3I-201. mRNA levels of selected genes whose expression is induced by *S.* Typhimurium infection was analyzed by qRT-PCR. Values represent the mean (± SEM) of GAPDH normalized transcript levels of the indicated genes, relative to DMSO treated uninfected cells. *: indicates statistically significant differences (*p*≤0.0004). (n. i.: non infected).

To gain insight into the signal transduction pathways triggered by *Salmonella* that result in the observed transcriptional responses we examined the profile of transcription-binding sites in the genes that were induced early and late in infection. Although binding sites for transcription factors known to be activated by MAP kinases or NF-κB signaling are prevalent among genes induced early after infection (Table S2), binding sites for signal transducer and activator of transcription 3 (STAT3) [22] are the most highly represented among genes whose expression was increased late in infection (Table S3 and Table S4). We also examined potential relationships among the *Salmonella*-induced genes using the STRING database, which curates data from multiple sources including physical as well as functional association between proteins [23]. This analysis also placed STAT3 as a central interaction node among the genes whose expression was stimulated by *Salmonella* late in infection (Fig. 2A). We therefore hypothesized that STAT3 may play a central role in the orchestration of the transcriptional response to *Salmonella*. Consistent with this hypothesis, we observed phosphorylation of STAT3 at both Y^705^ and S^727^, a measure of its activation, shortly after *S.* Typhimurium infection of cultured epithelial cells (Fig. 2B-2E and Figs. S3 and S4). STAT3 phosphorylation was observed throughout infection, with continuously increasing levels until at least 20 h post-infection (Fig. 2B and 2C). Consistent with its activation, immunofluorescence analysis detected phosphorylated STAT3 in the nucleus of ∼70% of *Salmonella* infected cells (Fig. 2D and 2E) while none was detected in uninfected cells. We then tested whether STAT3 was required for the transcriptional responses stimulated by *S.* Typhimurium infection. We examined the changes in mRNA levels of several genes whose expression had shown among the largest fold-change as a consequence of *Salmonella* infection (Table S1). We found that addition of the STAT3 inhibitor S31-201 [24] effectively blocked expression of the reporter genes in infected cells (Fig. 2F). These results indicate that *Salmonella* induces transcriptional responses through the activation of STAT3.

### *Salmonella* Typhimurium activation of STAT3 requires the SPI-1 T3SS effectors SopE, SopE2 and SopB

**Figure 3:**
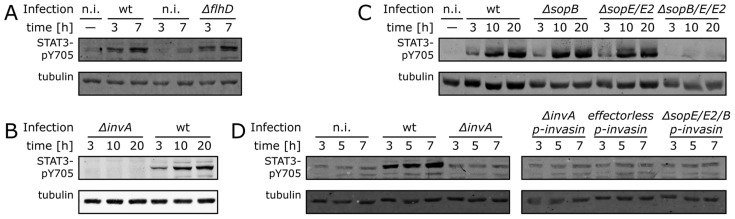
*Salmonella* stimulation of transcriptional responses in infected cells requires the SPI-1 T3SS effectors SopE, SopE2, and SopB. (**A**) *Salmonella* induces STAT3 activation in a flagellin-independent manner. Cultured Henle-407 cells were infected with wild-type *S.* Typhimurium (MOI = 10) or a *ΔflhD* mutant strain (which does not express flagella) (MOI = 100) for 1 h, resulting in an equal number of infected cells (Note: higher MOI was used for the flagellar mutant to compensate for its reduced ability to infect cells). Infected cells were then chased in the presence of gentamicin, lysed at the indicated times, separated by SDS-PAGE and probed by immunoblotting with antibodies to phosphorylated (activated) form of STAT3 (P-Y705), and tubulin (as loading control). (**B–D**) Henle-407 cells were infected (MOI = 10) for 1 h with wild-type *S.* Typhimurium, the SPI-1 T3SS-deficient *ΔinvA* mutant (**B**), or mutants defective in the effectors *sopE*, *sopE2*, and/or *sopB*, (**C**) or the indicated mutants carrying a plasmid encoding the *Yersinia pseudotuberculosis* invasin protein (**D**). Following chase in gentamicin containing medium, cells were lysed at the indicated times, separated by SDS-PAGE and probed by immuno blotting with antibodies to the phosphorylated (activated) form of STAT3 (P-Y705), unphosphorylated STAT3 (to detect total amount), and tubulin (loading control). (n. i.: non infected).

It has previously been reported that flagellin is capable of activating STAT3 [25]. However, a *S.* Typhimurium mutant unable to produce flagellin was equally capable of stimulating STAT3 compared to wild type (Fig. 3A). Instead, we found that STAT3 activation was strictly dependent on the SPI-1 T3SS system, since cells infected with a *S.* Typhimurium *invA* mutant, which is defective in this system [26], showed no STAT3 activation (Fig. 3B and Fig. S3). More specifically, we found that, like the transcriptional responses [12], STAT3 activation was specifically dependent on the SPI-1 T3SS effector proteins SopE, SopE2 and SopB (Fig. 3C and Fig. S5), which in a redundant manner activate Rho-family GTPases [10], [27]. Although mutations in each one of these effectors individually did not result in a significant reduction in bacterially-induced STAT3 phosphorylation, the simultaneous removal of these three effectors completely abolished *Salmonella*'s ability to activate STAT3 (Fig. 3C and Fig. S5). Since these three effector proteins also mediate bacterial internalization [28], we tested whether the presence of intracellular bacteria *per se* was sufficient to stimulate STAT3 activation. We expressed the *Yersinia pseudotuberculosis* invasin protein, which mediates bacterial uptake via the α4-β1 integrin receptors [29], in invasion-deficient *S.* Typhimurium mutant strains defective in the SPI-1 T3SS system (i. e. *ΔinvA*), specifically lacking the SPI-1 T3SS effectors SopE, SopE2 and SopB, or lacking all the known effectors of this T3SS (i. e. “effectorless mutant”). We found no STAT3 activation in cells infected with any of the *S.* Typhimurium mutant strains expressing invasin (Fig. 3D) despite the presence of equivalent numbers of intracellular bacteria (Fig. S6) to those that in the case of wild-type *Salmonella*, were sufficient to stimulate STAT3 activation (Fig. 2B and 2C). Taken together, these results indicate that *S.* Typhimurium stimulates the activation of STAT3 through specific signaling pathways that are dependent on the presence of the SPI-1 T3SS effector proteins SopE, SopE2 and SopB.

### *Salmonella* Typhimurium activates STAT3 by a non-canonical pathway that requires Abl and the p21-activated kinase (PAK)

**Figure 4:**
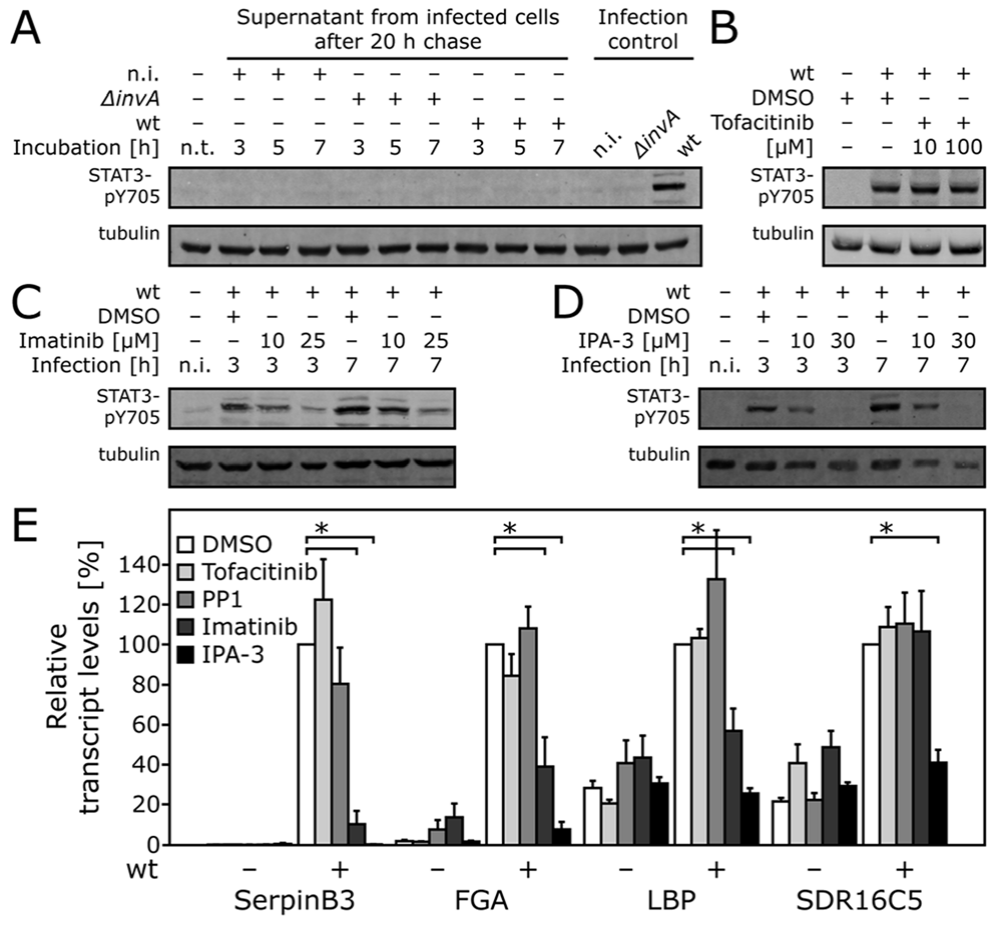
*Salmonella* Typhimurium activates STAT3 by a non-canonical pathway that requires Abl and PAK. (**A**) Culture supernatants from Henle-407 infected cells do not activate STAT3. Culture supernatants were obtained from Henle-407 cells 20 h after infection (MOI = 10) with either wild-type *S.* Typhimurium or the SPI-1 T3SS-defective *ΔinvA* mutant, filtered sterilized, and applied to uninfected Henle-407 cells. At different times after treatment cells were lysed, separated by SDS-PAGE and probed by immuno blotting with antibodies to the phosphorylated (activated) form of STAT3 (P-Y705), and tubulin (loading control). As a control, infected cells were analyzed for STAT3 activation in a similar fashion. (**B**) Activation of STAT3 by *S.* Typhimurium is JAK independent. Henle-407 cells were treated with the JAK inhibitor Tofacitinib at the indicated concentration and then infected (MOI = 10) with wild-type *S.* Typhimurium for 1 h, chased for additional 6 h in gentamicin containing medium in the presence of DMSO or Tofacitinib. Cells were then lysed and analyzed for STAT3 activation as indicated in (**A**). (**C**) Abl kinases are required for efficient *S.* Typhimurium-induced STAT3 activation. Cultured epithelial cells were pretreated with increasing concentrations of the STAT3 inhibitor Imatinib for 1 h, infected (MOI = 10) with wild-type *S.* Typhimurium, chased in the presence of the inhibitor, and at the indicated time cells were lysed and analyzed for STAT3 activation as described in (**A**). (**D**) *S.* Typhimurium-induced STAT3 activation requires PAK activity. Cultured epithelial cells were pretreated with increasing concentrations of the PAK inhibitor IPA-3 for 1 h, infected (MOI = 10) with wild-type *S.* Typhimurium, chased in the presence of the inhibitor, and at the indicated times cells were lysed and analyzed from STAT3 activation as indicated in (**A**). (**E**) Abl and PAK are required for the transcriptional responses to *S.* Typhimurium infection. Henle-407 cells were treated with inhibitors for Abl kinases (imatinib), PAK (IPA-3), JAK (Tofacitinib), and Src (PP1), infected (MOI = 10) with wild-type *S.* Typhimurium for 1 h, and chased for additional 3 h in gentamicin containing medium in the presence of DMSO or the inhibitors. mRNA levels of the selected indicated genes whose expression increase after *S.* Typhimurium infection were analyzed by qRT-PCR. Values represent the mean (± SEM) of GAPDH normalized transcript levels in infected cells relative to DMSO treated uninfected cells. *: indicates statistically significant differences (*p*≤0.02) of the indicated comparisons. (n. i.: non infected).

STAT3 activation most often results from the binding of various ligands to specific cell surface receptors of the Janus tyrosine kinase (JAK) family, which activate different members of the STAT family of cytoplasmic transcription factors [30]. It is well documented that STAT3 in particular can be potently activated by secreted cytokines such as IL-6 [30]. In fact, infection of macrophages by *S.* Typhimurium, which results in the activation of Toll like receptors and the production of cytokines, leads to STAT3 activation [31]. We therefore tested whether the STAT3 activation we observed in epithelial cells in response to *Salmonella* infection was the result of an autocrine and/or paracrine signaling pathway. We treated uninfected cultured epithelial cells with supernatants obtained from *S.* Typhimurium infected cells at different times after infection and examined STAT3 phosphorylation in the treated cells. We found no detectable STAT3 phosphorylation in cells treated with any of the infected cell supernatants (Fig. 4A and Fig. S7) indicating that STAT3 activation as a consequence of *Salmonella* infection is not likely to be the result of autocrine or paracrine pathways. These results suggested that the activation of STAT3 by *Salmonella* infection does not involve the canonical pathways dependent on the JAK tyrosine kinases. To test this hypothesis we examined the effect of a specific JAK tyrosine kinase inhibitor on *Salmonella*-induced STAT3 phosphorylation. We found that addition of the JAK inhibitor Tofacitinib (CP-690550) [32] had no effect on *S.* Typhimurium-induced STAT3 phosphorylation, even when supplied at a concentration 100 fold higher than that required for its inhibitory activity after stimulation by the addition of cytokines to cells that are known to activate this kinase in response to cytokines (Fig. 4B and Fig. S8). These results indicate that the *Salmonella*-induced STAT3 activation may be the result of a non-canonical pathway involving a tyrosine kinase(s) other than members of the JAK family. We reasoned that such a kinase must act downstream of Rho-family GTPase signaling since activation of STAT3 by *S.* Typhimurium required the SPI-1 T3SS effector proteins SopE, SopE2 and SopB (Fig. 3C), which exert their function by redundantly activating Rac1, Cdc42 and RhoG [10], [27]. A candidate to play this role is the Abelson tyrosine kinase (c-Abl) [33], which can be activated by *S.* Typhimurium infection [34] in a SopE/SopE2/SopB-dependent fashion (Fig. S9), and can directly phosphorylate STAT3 [35], [36]. We therefore tested whether *Salmonella*-induced STAT3 activation required this tyrosine kinase. We found that addition of imatinib, a specific inhibitor of c-Abl [37], significantly inhibited *Salmonella*-induced STAT3 activation (Fig. 4C). These results indicate that c-Abl is a component of the signaling cascade triggered by *Salmonella* that leads to STAT3 activation.

*Salmonella*-induced transcriptional reprogramming requires the activation of Cdc42 and Rac1 by the SPI-1 T3SS effector proteins SopE, SopE2 and SopB [10], [12], indicating that a downstream effector(s) of these Rho-family GTPases must be involved in the *Salmonella*-induced activation of c-Abl. The p21-activated kinases (PAKs) are well-characterized Rac and Cdc42 effector proteins, which are involved in a variety of signaling pathways [38]. PAK2 has been shown to phosphorylate and activate c-Abl [39]. Furthermore, *Salmonella* infection has been shown to robustly activate PAK2 [40]. We therefore reasoned that members of the PAK family would be good candidates to link the signaling events triggered by the *Salmonella* SPI-1 T3SS effector proteins with STAT3 activation. We found that addition of IPA-3, a specific inhibitor of PAK1/2/3 [41], effectively blocked *Salmonella*-induced STAT3 phosphorylation (Fig. 4D). Similar results were obtained after expressing a dominant-negative form of PAK3 (Fig. S10). These results indicate that members of the PAK family are the link between Rho family GTPase signaling stimulated by the *Salmonella* SPI-1 T3SS effectors and STAT3 activation, most likely through the activation of c-Abl.

We then tested whether the PAK and Abl kinases were required for the *Salmonella* stimulated transcriptional responses. We found that addition of PAK and (to a lesser extent) Abl kinase inhibitors blocked the transcriptional responses stimulated by *S.* Typhimurium infection (Fig. 4E). In contrast, addition of JAK or Src kinase inhibitors did not (Fig. 4E). Taken together, these results indicate that *S.* Typhimurium triggers pathogen-specific signaling pathways that lead to STAT3 activation and the reprogramming of gene expression in infected cells.

### Host cell gene expression reprogramming is required for *Salmonella* intracellular fitness and replication

**Figure 5:**
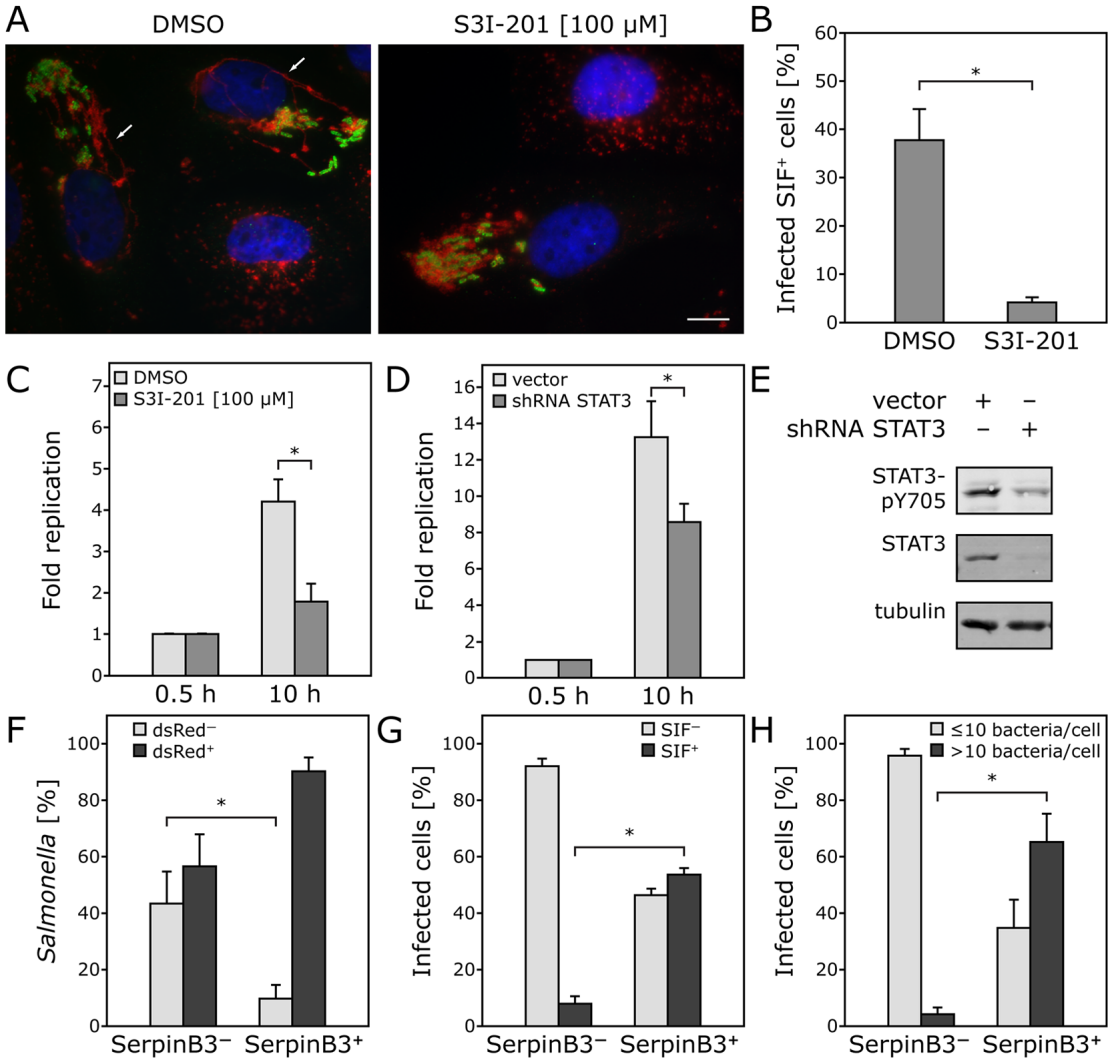
Host cell gene expression reprogramming is required for *Salmonella* intracellular fitness and replication. (**A**) and (**B**) STAT3 activity is required for the formation of *S.* Typhimurium-induced filaments (SIFs). Henle-407 cells treated with the STAT3 inhibitor S31-201 (100 µM) or DMSO were infected (MOI = 5) with wild-type *S.* Typhimurium for 1 h, chased for additional 8 h in gentamicin-containing medium, in the presence of the inhibitor or DMSO. Cells were then fixed, immuno stained for LAMP1 (red) and LPS (green) and the number of infected cells showing the presence of SIFs enumerated by epifluorescence microscopy (arrows denote the presence of SIFs). Values are the mean percentages (± SD) of infected cells showing SIF formation and represent three independent experiments in which at least 100 cells per condition were examined. *: indicates statistically significant differences (*p*≤0.005). (**C**–**E**) STAT3 activity is required for intracellular bacterial growth. Henle-407 cells were treated with the STAT3 inhibitor S31-201 (100 µM) or DMSO and then infected (MOI = 5) with wild-type *S.* Typhimurium for 1 h, chased for the indicated times in the presence of gentamicin and the inhibitor, and intracellular c.f.u. was enumerated by plating dilutions in the appropriate media (c. f. u. at time 0.5 h were as follows: DMSO treated: 7.6×10^5^±3.2×10^5^; S31-201 treated: 9.6×10^5^±4.9×10^5^ (**C**). Alternatively, *S.* Typhimurium growth was examined in HeLa cells transfected with an shRNA construct targeting STAT3 following the same procedure (c. f. u. at time 0.5 h were as follows: vector treated: 6.6×10^5^±2.4×10^5^; STAT3 shRNA treated: 7.3×10^5^±1.8×10^5^ (**D**). Numbers represent fold replication and are the mean (± SD) of three independent determinations (**C** and **D**). *: indicates statistically significant differences (*p*≤0.007). Levels of STAT3 in shRNA-targeted cells were determined by immunoblotting with the indicated antibodies (**E**). (**F**) *S.* Typhimurium shows increased fitness and greater replication in SerpinB3-positive (i. e. transcriptionally reprogrammed) cells. Henle-407 cells were infected (MOI = 10) for 1 h with *S.* Typhimurium encoding dsRed under the control of an arabinose inducible promoter, chased for 17 h in gentamicin supplemented medium, and further incubated for 3 h in the presence of 0.1% arabinose. Cells were then fixed, immuno stained for LPS, endogenous SerpinB3, and DNA, and bacteria expressing dsRed in SerpinB3-positive or negative cells were enumerated by epifluorescence microscopy. Fitness was measured by evaluating the ability of intracellular bacteria to produce dsRed protein. Values are the means (± SD) of the percentages of the bacteria showing dsRed expression after addition of arabinose in SerpinB3-positive or negative cells, and represent data from three independent experiments in which at least 100 infected cells were examined (**F**) (*: *p*≤0.007). (**G**) Increased *Salmonella*-induced filament (SIFs) formation in SerpinB3-positive (i. e. transcriptionally reprogrammed) cells. Cultured epithelial cells were infected (MOI = 10) for 1 h with *S.* Typhimurium, chased for 20 h in gentamicin-supplemented medium, fixed, immuno stained for LAMP1 (to stain for SIFs), LPS and SerpinB3, and analyzed by epifluorescence microscopy. Depicted are the mean percentages (± SD) of infected, SerpinB3-positive cells that show the presence (SIF+) or absence (SIF−) of *Salmonella*-induced filaments (SIFs) from three independent experiments in which at least 100 cells per condition were analyzed (*: *p*≤0.001). (**H**) Increased bacterial replication in SerpinB3-positive cells. Cells were infected with wild type *S.* Typhimurium, (MOI = 10) chased for 20 h in gentamicin supplemented medium, fixed, immuno stained for LPS (to stain for *Salmonella*), endogenous SerpinB3 and DNA, and the total number of bacteria in SerpinB3-positive or negative cells were enumerated by epifluorescence microscopy. Values are the means (± SD) of the percentages of SerpinB3-positive or negative cells that had a bacterial load of up to 10 bacteria or more than 10 bacteria, and represent three independent experiments in which at least 100 cells per condition were examined (*: *p*≤0.00002).

The delineation of the signaling pathways stimulated by *Salmonella* leading to changes in host-cell gene expression provided us with an opportunity to evaluate the potential influence of these responses in *Salmonella* biology. We investigated whether specific inhibitors of the signaling pathways leading to transcriptional responses altered the intracellular growth of this pathogen. *S.* Typhimurium replication and survival within cells have been correlated with its ability to form a specialized bacteria-containing membrane-bound compartment that is characterized by the presence of tubular-like membranous structures known as SIFs (for *Salmonella* induced filaments) that can be stained with the endosomal protein LAMP1 [42]. We found that addition of the specific STAT3 inhibitor S31-201 blocked the formation of SIFs (Fig. 5A and 5B). Furthermore, addition of the STAT3 inhibitor (Fig. 5C) or RNAi-mediated depletion of STAT3 (Fig. 5D and 5E) significantly impaired *S.* Typhimuirum intracellular growth without affecting host cell survival (Fig. S11).

We made the observation that, in epithelial cells, only a subset of *Salmonella*-infected cells undergo transcriptional reprogramming, as indicated by the ability of these cells to express SerpinB3 as well as other genes whose expression levels were shown by microarray measurements to be increased at later times after *Salmonella* infection (see Text S1). Although the *in vivo* relevance of this observation is unknown, it provided us with an opportunity to investigate potential differences in the biology of *Salmonella* when localized within cells that have (SerpinB3-positive) or have not (SerpinB3-negative) undergone changes in their gene-expression profile as a consequence of bacterial infection. We first investigated potential differences between the fitness of bacteria present within these populations of host cells. We used a *S.* Typhimurium strain that expresses the red fluorescence protein (dsRed) under the control of an arabinose-inducible promoter and measured fitness as the capacity of *Salmonella* to produce the red fluorescence protein upon addition of arabinose. We found that most *Salmonella* in SerpinB3-positive cells were able to express dsRed (Fig. 5F). In contrast, a significantly higher proportion of bacteria in SerpinB3-negative cells were unable to activate dsRed expression (Fig. 5F). Differences in dsRed expression could not be explained by differences in the accessibility of the inducer in SerpinB3-positive or SerpinB3-negative infected cells since the total ratio of dsRed+ vs. dsRed- bacteria was maintained in experiments in which the inducer was added to bacteria that had been released from cells (Fig. S12). We also found that the number of *Salmonella* associated with SIFs was significantly higher in cells expressing SerpinB3 than in cells that were not (Fig. 5G). Consistent with the increased bacterial fitness and the increased number of SIFs, we found a significantly higher number of *Salmonella* within cells that have undergone gene expression changes (i. e. SerpinB3 positive) than within cells that have not (i. e. SerpinB3-negative) (Fig. 5H and Fig. S13). Taken together these results indicate that *Salmonella* triggers signaling events that lead to changes in host-cell gene expression and render the host cell more permissive for bacterial growth and survival.

## Discussion

**Figure 6:**
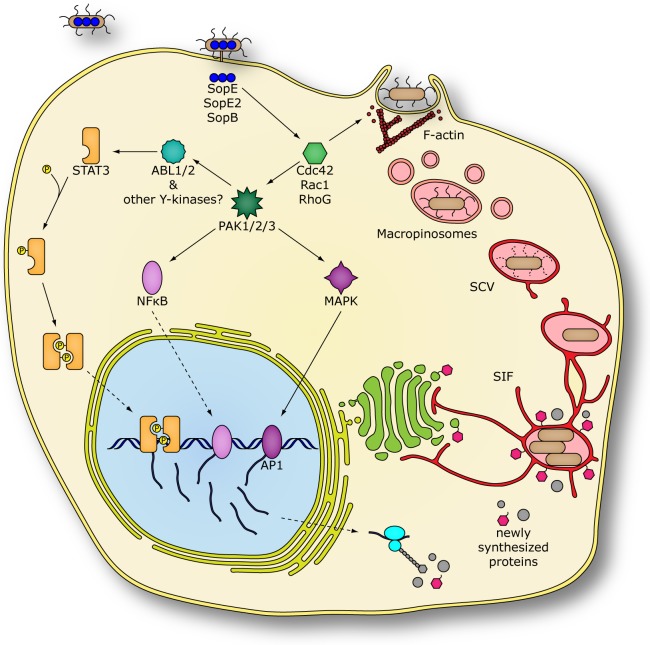
Bacterially-induced reprogramming of host cell gene expression is required for efficient *Salmonella* replication. *S.* Typhimurium infects intestinal epithelial cells by delivering effector proteins into the host cell, using its SPI-1 T3SS. The effector proteins SopB, SopE and SopE2 activate the small GTPases Cdc42, Rac1 and RhoG in a redundant manner, thus inducing membrane ruffling and bacterial uptake. Internalized bacteria reside in macropinosomes, which then maturate into *Salmonella* containing vacuoles (SCVs). Bacterial effector-stimulated small GTPases also activate members of the p21 activated kinase family (PAK). These serine/threonine kinases trigger downstream signaling of mitogen-activated protein kinases (MAPKs) and nuclear factor kappa B (NFκB) [11], [40], [43], resulting in activation of additional transcription factors. In addition, activated PAK proteins also phosphorylate members of the Abl kinase family, thereby triggering auto-phosphorylation of Abl proteins for full activation, subsequently leading to the activation the cytoplasmic transcription factor STAT3. Products of STAT3-controlled genes ultimately influence vesicular trafficking between cellular compartments and the SCV, contributing to the formation of *Salmonella* induced filaments (SIFs), which characterize a fully mature, replication-competent *Salmonella*-containing vacuole.

It is well established that the interaction of microbial pathogens with mammalian cells often leads to significant changes in host-cell gene expression [13]. These responses are most often the result of the stimulation of innate immune receptors by conserved bacterial products, which lead to “hard-wired” transcriptional outputs. *Salmonella*, however, has evolved the additional ability to stimulate transcriptional responses independent of the activation of innate immune receptors [11], [12], [43]. This specific adaptation, which requires effectors of the SPI-1 T3SS, allows *Salmonella* to potentially trigger this response in cells, such as those of the intestinal epithelium, that are subject to robust regulatory mechanisms to prevent the stimulation of innate immune receptors [14]–[16]. In this study we have defined a host-cell signaling pathway leading to *Salmonella*-induced changes in gene expression in epithelial cells and found that STAT3 plays a central role in their orchestration. We have found that *Salmonella* activates STAT3 through a non-canonical pathway that does not require JAK kinases. Instead, this pathway is triggered by the SPI-1 T3SS effectors SopE, SopE2 and SopB, which through Rho-family GTPases, stimulate PAK and Abl tyrosine kinases leading to STAT3 activation (Fig. 6).

Our examination of the host cell gene expression in *Salmonella*-infected cells has revealed a very complex pattern that changes significantly over time. However, our results suggest that the pattern of gene expression is largely established by mechanisms operating shortly after infection. In fact, infection of cells with a conditionally lethal mutant of *Salmonella* that dies shortly after infection led to a transcriptional response that showed significant overlap with that observed in cells infected with wild-type *Salmonella*. We therefore hypothesize that a significant proportion of the changes in the transcriptional response to *Salmonella* late infection is defined by the intrinsic dynamics of the responses triggered shortly after infection. In support of this premise, the expression of several transcription factors was seen elevated shortly after infection (although not late in infection). It is therefore possible that the early stimulation of expression of these transcription factors may be central to the orchestration of changes in gene expression later in infection. The potential mechanism by which these transcription factors may influence the cellular response to *Salmonella* infection is unknown but it is unlikely to involve autocrine or paracrine mechanisms since we found that culture supernatants of infected cells did not stimulate transcriptional responses in non-infected cells.

We speculate that at least some of the changes in gene expression stimulated by *Salmonella* are also under post-transcriptional regulatory control. For example, the SerpinB3 and SerpinB4 proteins were not detected until 10 h after infection despite the fact that we detected a very significant increase in their mRNA levels shortly after *Salmonella* infection. One of the proteins whose expression is induced and maintained throughout infection is the post-transcriptional regulator tristetraprolin (TTP). TTP is an RNA-binding protein that controls gene expression by modulating mRNA decay of messages containing a consensus adenylate and uridylate-rich (ARE) element in the 3′-untranslated region [44]. The extent to which TTP modulates the gene expression changes resulting from bacterial infection is not known but it is intriguing to hypothesize that by accelerating the decay of certain messages TTP may help to shape the nature of the response stimulated by *Salmonella* late in infection.

There is abundant evidence for a role of the transcriptional responses to *Salmonella* as well as to other pathogens in the stimulation of inflammation [19], [20]. In fact, studies have shown that early responses to a variety of different pathogens often lead to the production of pro-inflammatory cytokines [13]. However, there is remarkably little evidence to support a role for pathogen-induced transcriptional responses to modulate cell autonomous processes that may actually help pathogen replication. We found that cells that have undergone changes in gene expression, support more bacterial growth and harbor fitter bacteria than cells that have not. We found that the formation of *Salmonella*-induced filaments (SIFs), membranous structures harboring *Salmonella* that characterize the replication-competent intracellular compartment [42], was more efficient in cells that have undergone changes in gene expression. The mechanisms by which changes in gene expression contribute to *Salmonella* replication and the formation of its intracellular niche are likely to be multifactorial and multigenic. Indeed among the genes whose expression was increased as a consequence of *Salmonella* infection there are several that could potentially contribute to the formation of *Salmonella*'s intracellular niche such as genes involved in G protein signaling, lipid metabolism, vesicle traffic, or protease inhibition. However, more studies will be required to understand the mechanism by which the transcriptional re-programing aids the intracellular growth of *Salmonella*.

We have described here a mechanism by which *S.* Typhimurium renders infected cells more permissive for its survival and replication by stimulating changes in gene expression through a pathogen-specific mechanism. The stimulated signaling mechanisms involve pathways for which suitable inhibitors are available and at advance stages of clinical development for a variety of applications [45]–[47]. Therefore these findings may provide the bases for the development of novel therapeutic strategies to combat *Salmonella* infection that would target relevant host pathways. The results described here constitute a remarkable example of how pathogens modulate cellular functions for their own benefit.

## Materials and Methods

### Bacterial strains, growth conditions, cDNA constructs, and other reagents

All bacterial strains used in this study are derived from *S.* Typhimurium strain SL1344 and are listed in Table S5. For trans-complementation studies, expression plasmids derived from pBAD24 encoding *sopE*, *sopE2*, or sopB were introduced into the effectorless strain SB1011. *S.* Typhimurium was grown under conditions that increase expression of the SPI-1 T3SS [48], and when required, 0.1% arabinose was added to the medium to induce the expression of genes under the control of the arabinose-inducible p*araABC* promoter. To grow the *S.* Typhimurium *Δasd* mutant, the growth media was supplemented with 50 µg/ml sodium diaminopimelic acid (L-DAP, Sigma-Aldrich). Bacterial growth curves in Luria-Bertani (LB) broth supplemented with the different inhibitors used in this study were obtained simultaneously using a multi-well plate and a plate reader (TECAN infinite M1000). Dominant negative mPak3 has been previously described [40]. Antibodies and other reagents were purchased from the indicated companies: rabbit-anti-TTP and rabbit-anti-SerpinB3/B4 (Abcam); rabbit-anti-*Salmonella* O Group B Antiserum (Becton Dickson); rabbit-anti-Phospho-STAT3 (Ser727), rabbit-anti-Phospho-STAT3 (Tyr705), and rabbit-anti-EGR1 (Cell Signaling Technology); mouse-anti-LAMP1 clone H4A3, (Developmental Studies Hybridoma Bank); rabbit-anti cFOS, mouse-anti-SerpinB3, mouse-anti-SerpinB4, and mouse-anti-STAT3 (Santa Cruz Biotechnology); rabbit-anti-actin and mouse-anti-tubulin (Sigma-Aldrich); secondary antibodies (Molecular Probes); 4′,6-diamidino-2-phenylindole (DAPI) and PP1 (both Sigma-Aldrich); S3I-201 (Calbiochem); Imatinib (Enzo Life Sciences); Tofacitinib (SYN KINASE); IPA-3 (Santa Cruz Biotechnology), and λ-Phosphatase (NEB).

### Cell culture and bacterial infections

The human epithelial cell lines Henle-407 and HeLa, the human embryonic kidney epithelial cell line HEK-293T, and the mouse macrophage RAW cells were cultured in antibiotic free Dulbecco's Modified Eagle Medium (DMEM, Gibco) supplemented with 10% bovine calf (Henle-407) or bovine fetal (HeLa,HEK-293T and RAW) sera. The human liver epithelial cell line HepG2 was cultured on antibiotic-free minimal essential medium (MEM, Gibco) supplemented with 10% FBS. For bacterial infections, cells at a confluency of 80% were washed with Hank's buffered salt solution (HBSS) and allowed to equilibrate in HBSS for 15 min at 37°C. Cells were then infected for 1 h with *S.* Typhimurium strains at the multiplicities of infection (MOI) indicated in the Figure legends. To determine ABL1 activation, 24 h serum starved HEK-293T cells were lifted and resuspended in 500 µl HBSS, gently rocked for 2.5 h in the incubator before infected in suspension. In the case of infection with the flagellar mutant, cells were centrifuged for 5 min at 2,000 rpm after addition of the bacteria to facilitate bacteria/host cell contact. Infected cells were then washed once with HBSS and were incubated for 2 h in DMEM containing 50 µg/ml gentamicin, washed again once with HBSS and further incubated in DMEM containing 10 µg/ml gentamicin for the indicated times.

### Bacterial internalization and survival

Bacterial internalization and survival within host cells was examined using a gentamicin protection assay as described before [49]. Briefly, cultured epithelial cells grown in a 12 well plate were infected for 1 h and incubated in the presence of gentamicin as described above. Cells were washed twice with HBSS and then lysed in 300 µl 0.1% Sodium Deoxycholate (DOC) in HBSS to release their bacterial content. Multiple dilutions in buffered saline with gelatine (BSG) were plated onto LB medium, which in case of the *Δasd* strain was supplemented with DAP, to determine the concentration of living bacteria by quantifying colony forming units (c.f.u.).

### Statistical analysis

Statistical significance was calculated by a one-tail distributed paired Student's t-test. Resulting *p*-values of less than 0.05 were considered significantly different.

### Drug treatment

Epithelial cells were pretreated for 1 h in HBSS containing the different inhibitors at the indicated concentrations. Cells were then infected for 1 h and further chased for the indicated periods always in the presence of the respective drugs before harvested either for Western blot analysis, immunofluorescence or qRT-PCR. None of the compounds exhibited any inhibitory effect on bacterial growth (Fig. S14).

### Detection of apoptosis

Henle-407 cells grown in a 6 well plate were pretreated with DMSO or S3I-201 for 1 h in HBSS, infected with *S.* Typhimurium for 1 h (MOI = 5), chased for 2 h in DMEM containing 50 µg/ml gentamicin and further incubated in DMEM containing 10 µg/ml gentamicin for another 6 h, always in the presence of drugs. Subsequently cells were trypsinized and fixed with 4% PFA/PBS for 15 min at RT. Apoptosis was detected in a TUNEL reaction using the “*in situ* Cell Death Detection Kit, Fluorescein” (Roche) according to the manufacture's protocol in combination with a fluorimetric analysis employing a FACSCalibur (BD Biosciences).

### Western blot

Epithelial cells were lysed in 2× SDS sample buffer. Proteins contained in equal volumes of the cell lysates were separated by SDS-PAGE and subsequently transferred to PVDF membranes. After blocking with 3% BSA (phospho specific blots) or 5% milk in Tris buffered saline (TBS), membranes were probed with the respective primary and secondary antibodies in blocking solution supplemented with 0.02% SDS and 0.1% Tween 20. The blots were analyzed using either the Odyssey LI-COR system together with the LI-COR Odyssey application software or visualized by enhanced chemiluminescence (ECL). When indicated, samples were dephosphorylated by incubating them in the presence of 1,200 units λ-Phosphatase (NEB) for 2 hs at 30°C prior to SDS-PAGE and Western blot analysis.

### siRNA-mediated knockdown

Endogenous STAT3 was silenced by using the pSUPER RNAi System (oligoengine). Briefly, a 60 mer oligonucleotide (5′-GATCCCCGGCGTCCAGTTCACTACTATTCAAGAGATAGTAGTGAACTGGACGCCTTTTTC-3′), including the STAT3 target site described elsewhere [50], was cloned into the BglII and HindIII side of the pSUPER vector. Cells were transfected using Lipofectamine2000 (Invitrogen), infected (MOI = 2.5) 48 h or 72 h later with *S.* Typhimurium for 1 h and then incubated in gentamicin (50 µg/ml)-supplemented DMEM. At the indicated time points cells were lysed with 0.1% DOC in HBSS to release intracellular bacteria as described above. Cell lysates were examined for the presence of c. f. u. and analyzed by western blot for the presence of endogenous STAT3 (total and phospho-Y705) as well as tubulin as a loading control.

### Immunofluorescence

Human epithelial cells grown on glass coverslips were infected with different *S.* Typhimurium strains as described above, washed once with HBSS and fixed in 4% PFA/PBS for 15 min at RT. For SerpinB3 staining, cells were permeabilized with 0.1% Triton X-100 in PBS for 2 min at RT, washed once with PBS and then incubated in blocking solution (1% BSA, 0.01% Triton X-100 in PBS) for 20 min at RT. Staining of phosphorylated proteins was done overnight at 4°C in a wet chamber. For staining of *Salmonella*-induced filaments (SIFs), cells on glass coverslips were directly treated with blocking solution (3% BSA, 0.1% Saponin, 50 mM NH_4_Cl in PBS) for 20 min at RT. Subsequently, glass coverslips were incubated in the respective blocking solution containing primary antibodies against the protein of interest for 1 h at RT, washed 3 times in blocking solution and incubated another 30 min at RT in blocking solution containing a mixture of secondary antibodies coupled to Alexa dyes and DAPI for DNA stain. Finally, glass coverslips were washed twice with blocking solution, PBS and water before they were mounted on glass slides and examined by epifluorescence microscopy (Nikon Diaphot) or spinning disk confocal microscopy (Improvision) using a Nikon TE2000 microscope, a Hamamatsu EM-CCD digital camera and Volocity software (Improvision).

### Immunofluorescence microscopy-based bacterial replication assay

Infected epithelial cells on glass coverslips were fixed and immunostained for SerpinB3, LPS (to stain bacteria) and DAPI (to stain DNA) as described above. Cells were randomly imaged using a combination of epifluorescence and bright field microscopy to simultaneously detect SerpinB3 production, presence of *Salmonella* and the outlines of the epithelial cell. These images were then analyzed with ImageJ software (http://rsbweb.nih.gov/ij/) to quantify the proportion of SerpinB3-positive or negative *Salmonella* infected cells and to quantify the number of intracellular bacteria. SerpinB3-positive and negative cells were then categorized by the number of intracellular bacteria that they harbor.

### Fitness assay

Bacterial fitness was evaluated by the ability of *S.* Typhimurium to produce the dsRed fluorescence protein. Henle-407 cells were infected as described above for 1 h with a strain of *S.* Typhimurium carrying a plasmid that encodes the dsRed gene whose expression is under the control of an arabinose-inducible promoter. After 17 h incubation in arabinose free DMEM, expression of dsRed was induced by addition of 0.1% arabinose to the culture medium for 3 h. Cells were fixed, and immunostained for SerpinB3 and LPS, and with DAPI for DNA. The proportion of bacterial cells expressing dsRed in SerpinB3-positive and SerpinB3-negative cells was calculated by evaluating the number of dsRed-positive and dsRed-negative bacteria in a minimum of 100 cells in randomly taken images obtained by epifluorescence microscope using ImageJ software. To control for the accessibility of arabinose in SerpinB-positive or negative cells, bacteria were released from infected cells after 17 h incubation in arabinose free DMEM by addition of 0.1% DOC, pelleted, and resuspended in HBSS or HBSS containing 0.1% arabinose. Bacteria suspensions were incubated for additional 3 h at 37°C, recovered by centrifugation, fixed in 4% PFA/PBS for 15 min at RT and immunostained with an antibody against LPS. The proportion of bacteria producing dsRed was determined by epifluorescence microscopy. At least 100 bacteria per condition were quantified.

### *Salmonella*-induced filament (SIF) formation assay

Formation of SIFs was evaluated by immunofluorescence microscopy as follows. Inhibitor-treated or untreated cultured epithelial cells were infected (MOI = 10) for 1 h, fixed after the indicated periods of incubation in gentamicin containing DMEM, and immunostained for SIFs with a monoclonal antibody to LAMP1, a marker for this compartment. When required, cells were also stained with an antibody to SerpinB3. The number of infected cells exhibiting SIFs was determined by analyzing randomly taken images obtained by epifluorescence microscopy using the ImageJ software. At least 100 infected cells per condition were evaluated.

### FACS sorting and nucleic acid extraction from sorted cells

Uninfected and infected Henle-407 cells were washed once with PBS and treated with trypsin/EDTA for 5 min at 37°C. Detached cells were collected in DMEM containing 5% bovine serum albumin (BSA), centrifuged at 1000 rpm for 5 min, fixed in 1 ml RNase free PBS containing 4% PFA for 15 min at RT, transferred into a fresh 2 ml reaction tube and washed once with RNase free PBS. Cells were resuspended in 1.5 ml RNase free blocking buffer (3% BSA, 0.1% Saponin, 50 mM NH_4_Cl in PBS) containing primary antibodies against SerpinB3 and were incubated for 1 h at 4°C. Cells were then washed three times with blocking buffer and were incubated for additional 30 min at 4°C in blocking buffer containing the secondary antibody coupled to Alexa-488. Cells were washed again, resuspended in 3 ml 1% RNase free BSA in PBS, filtered through a 30 µm nylon mesh and finally subjected to FACS sorting. FACS sorting was done at the Yale Cell Sorting Core Facility using a BD FACSAria. Sorted cells were collected in RNase free PBS containing 2 ml 1% BSA in PBS. Total DNA and RNA from equal numbers of sorted Henle-407 was isolated using the “DNAeasy Blood and Tissue” kit (QIAGEN), following the manufacture's description for cultured animal cells. Briefly, pelleted cells were resuspended in 20 µl proteinase K and 200 µl AL buffer (QIAGEN) and incubated for 10 min at 56°C. After addition of 200 µl ethanol, the lysate was loaded onto a “DNeasy Mini spin column”, bound nucleotides subsequently washed with 500 µl AW1 buffer and AW2 buffer (QIAGEN) and finally eluted with 100 µl RNase free water. Remainining contaminant DNA was removed by treatment with DNAse I (Roche) for 15 minutes.

### Quantitative real-time PCR

RNA isolation, in vitro transcription and quantitative real-time PCR was carried out as described elsewhere (Bruno et. al.). Briefly, 24 h serum starved Henle-407 cells were infected with wild type *S.* Typhimurium for 1 h and chased for additional 3 h. Total RNA was isolated using TRIzol (Invitrogen) reagent according to the manufacture's protocol. RNA was then further purified using the “RNeasy Mini Kit” (QIAGEN). Following DNAse treatment, RNA was transcribed using the iScript reverse transcriptase (BIO RAD). Transcript levels were determined using gene specific primers sets (Table S6), that have been designed by PrimerBank (http://pga.mgh.harvard.edu/primerbank/), and the iCycler real time PCR machine (BIO RAD).

### Microarray analysis

Total RNA was isolated from infected (MOI = 30), serum starved Henle-407 cells at the indicated time points as described for quantitative real-time PCR. Samples were submitted to the Yale University W.M. Keck facility, where further sample preparation and hybridization to the Affymetrix HG U133 Plus 2.0 gene arrays was performed, using an Affymetrix GeneChip Instrument System according to the manufacturer's recommendations. Imaging was done on an Affymetrix GeneChip scanner 3000 according to Affymetrix standard protocols (GeneChip Expression Analysis Technical Manual, Affymetrix, 2004), while the raw data was processed and normalized using “affy” package in Bioconductor 2.9 [51]. For each experimental group, fold changes in gene expression observed for each strain were calculated relative to the uninfected control.

## Supporting Information

Figure S1 SPI-1 T3SS activity, uptake and intracellular survival of the *S.* Typhimurium *Δasd* mutant. (**A**) The SPI-1 T3SS of the *S.* Typhimurium *Δasd* mutant looses its activity upon withdrawal of L-DAP. The *S.* Typhimurium *Δasd* mutant was grown in the absence of L-DAP for 0 or 6 hs and its ability to enter into cultured epithelial cells (a very sensitive functional readout of SPI-1 T3SS function) was evaluated by the gentamicine protection assay after plating in the presence of L-DAP (see Methods). Invasion values are expressed relative to those obtained after growth of the strain in the presence of L-DAP, which were considered 1 and are the mean ± standard deviation of three repetitions. (**B**) Uptake and intracellular survival of the *S.* Typhimurium *Δasd* mutant. The SPI-1 T3SS Henle-407 cells were infected (MOI = 5) with wild-type *S.* Typhimurium or the isogenic *Δasd* mutant derivative for 1 h and chased in the presence of gentamicin. At the indicated times cells were lysed, bacteria released, plated (in the case of the *Δasd* mutant in the presence of L-DAP), and the number of colony forming units determined. Depicted are the mean values (± SEM) of three independent experiments. The detection limit for this experiment was ∼10 c. f. u.
(TIF)

Figure S2 Venn diagram depicting the number of unique and common genes whose expression changed at least 5 fold at the indicated times after infection of Henle-407 cells with wild type *S.* Typhimurium or the isogenic *Δasd* mutant.
(TIF)

Figure S3 The *S.* Typhimurium Δasd mutant induces STAT3 activation. HeLa cells were infected (MOI = 10) with wild-type *S.* Typhimurium or the isogenic *ΔinvA* (T3SS-defective) or *Δasd* mutants for 1 h. Following chase in gentamicin containing medium, cells were lysed at the indicated times, separated by SDS-PAGE and probed by immuno blotting with antibodies to the phosphorylated (activated) form of STAT3 (P-Y705) and tubulin (loading control). (n. i.: not infected).
(TIF)

Figure S4 Phosphatase treatment eliminates the reactivity of the antibody directed to phosphorylated STAT3. Henle-407 cells were infected (MOI = 10) with wild-type *S.* Typhimurium for 1 h. Following chase in gentamicin containing medium for 3 hs, cells were lysed, separated by SDS-PAGE and probed by immuno blotting with antibodies to the phosphorylated (activated) form of STAT3 (P-Y705), and tubulin (loading control). When indicated, samples were treated with λ-phosphatase for 30 minutes prior to loading. (n. i.: not infected).
(TIF)

Figure S5 *Salmonella* stimulation of transcriptional responses in infected cells requires the SPI-1 T3SS effectors SopE, SopE2, and SopB. Henle-407 cells were infected (MOI = 10) for 1 h with wild-type *S.* Typhimurium, a mutants defective in all known effectors of the SPI-1 T3SS (effectorless), or the effectorless mutant complemented with plasmid-borne wild type alleles of *sopE*, *sopE2*, or s*opB*, as indicated. Following chase in gentamicin containing medium, cells were lysed at the indicated times, separated by SDS-PAGE and probed by immuno blotting with antibodies to the phosphorylated (activated) form of STAT3 (P-Y705), and actin (loading control).
(TIF)

Figure S6 Invasin-mediated internalization of SPI-1 T3SS-defective *S.* Typhimurium. Henle-407 cells were infected (MOI = 10) with the indicated strains of *S.* Typhimurium for 1 h. Following chase in gentamicin containing medium for 2 h cells were lysed and c. f. u. enumerated by plating dilutions of the bacterial suspension. Values represent the mean (± SD) of three independent measurements.
(TIF)

Figure S7 Culture supernatants from Henle-407 infected cells do not activate STAT3. Culture supernatants were obtained from Henle-407 cells 2 or 13 h after infection (MOI = 10) with either wild-type *S.* Typhimurium or the SPI-1 T3SS-defective *ΔinvA* mutant, filtered sterilized, and applied to uninfected Henle-407 cells (**A** and **B** panels, respectively). At different times after treatment cells were lysed, separated by SDS-PAGE and probed by immuno blotting with antibodies to the phosphorylated (activated) form of STAT3 (P-Y705), and tubulin (loading control). As a control, infected cells were analyzed for STAT3 activation in a similar fashion.
(TIF)

Figure S8 Effectiveness of the JAK inhibitor Tofacitinib. HepG2 cells (pretreated with 1.0 µM Tofacitinib or DMSO were incubated for the indicated periods with supernatant from activated RAW macrophages in the presence of the inhibitor or DMSO. Cell lysates were applied to SDS-PAGE and immuno blotting.
(TIF)

Figure S9 *Salmonella* activates ABL1 in a *sopB/sopE*/*sopE2*-dependent manner. Cultured HEK-293T cells were infected (MOI = 30) with the indicated strains of *S.* Typhimurium for 30 min in HBSS. Cells were lysed, separated by SDS-PAGE and probed by immuno blotting with antibodies to the phosphorylated (activated) form of ABL1 (P-Y412) and total ABL1 as a loading control (n.i.: non infected).
(TIF)

Figure S10 Expression of dominant negative Pak3 reduces *Salmonella* induced activation of STAT3. Cultured Henle-407 cells were thansfected with a plasmid encoding dominant negative Pak3 or the vector control. Transfected cells were subsequently infected (MOI = 10) with wild-type *S.* Typhimurium for 1 h and chased in the presence of gentamicin. At the indicated times cells were lysed, separated by SDS-PAGE and probed by immuno blotting with antibodies to the phosphorylated (activated) form of STAT3 (P-Y705), and tubulin (loading control). The relative levels of STAT3 activation in the infected cells were calculated after quantification with the Odyssey LI-COR system and are expressed relative to the phospho-STAT3 signal in the control sample 6 h after infection.
(TIF)

Figure S11 *S.* Typhimuirum infection of cultured cells in the presence of a STAT3 inhibitor does not result in significant increase of apoptosis. Henle-407 cells were infected for 1 h with *S.* Typhimurium (m. o. i. 5) in the presence of the STAT3 inhibitor S31-201 or DMSO and the percentage of cells undergoing apoptosis was determined 9 hs after infection by TUNEL statining. Notice that greater than 60% of cells were infected in this experiment and therefore the minor increase in TUNEL positive cells observed overall, which was not statistically significant, is of no biological relevance.
(TIF)

Figure S12 Arabinose-induced expression of dsRed in *S.* Typhimurium after their release from intracellular compartments. Henle-407 cells were infected for (MOI = 10) 1 h with *S.* Typhimurium expressing dsRed under the control of an arabinose-inducible promoter. After 17 h chase in gentamicin supplemented medium cells were incubated for additional 3 h in the presence of 0.1% arabinose to induce dsRed expression. Alternatively, bacterial cells were released, transferred into a test tube containing HBSS and 0.1% arabinose and incubated for 3 h at 37°C. Bacterial cells were fixed, immuno stained for LPS, and analyzed by epifluorescence microscopy to determine the percentage of bacteria expressing dsRed. Numbers are the percentages of dsRed positive and negative bacteria and represent the means (± SD) of three independent experiments in which at least 100 bacteria were quantified. *: indicates statistically significant differences (*p*≤0.03).
(TIF)

Figure S13 Host cell gene expression reprogramming is required for efficient *Salmonella* intracellular replication in HeLa cells. HeLa cells were infected (MOI = 10) for 1 h with *S.* Typhimurium chased for 20 h in gentamicin supplemented medium, fixed, immuno stained for LPS (to stain for *Salmonella*), endogenous SerpinB3 and DNA, and the total number of bacteria in SerpinB3-positive or negative cells were enumerated by epifluorescence microscopy. Values are the means (± SD) of the percentages of SerpinB3-positive or negative cells that had a bacterial load of up to 10 bacteria or more than 10 bacteria, and represent three independent experiments in which at least 100 cells per bacterial strain were examined. *: indicates statistically significant differences (*p*≤0.003).
(TIF)

Figure S14 Effect of chemical inhibitors on *S.* Typhimurium growth. *S.* Typhimurium was cultured on LB containing the indicated inhibitors. Bacterial growth was monitored every 10 min at an OD_600_ for more than 18 h at 37°C. Depicted are the mean values (+ SD) of three technical replicates.
(TIF)

Table S1 Microarray analysis of the transcriptional responses induced by different strains of *Salmonella* Typhimurium. Depicted are alphabetically sorted genes upregulated at least 3-fold after infection in at least one condition.
(PDF)

Table S2 Transcription binding sites in genes whose expression increased at least 4-fold 4 h after infection^1^.
(PDF)

Table S3 Transcription binding sites in genes whose expression increased at least 4-fold 10 h or 20 h after infection^1^.
(PDF)

Table S4 Predicted STAT3 binding sites in genes that are at least 4-fold increased 10 h or 20 h after infection^1^.
(PDF)

Table S5 Bacterial strains.
(PDF)

Table S6 Primers used in this study.
(PDF)

Text S1 Heterogeneity in *Salmonella* Typhimurium-induced gene expression in cultured epithelial cells.
(PDF)
