# Supplementary figures and images for: Salmonella Modulation of Host Cell Gene Expression Promotes Its Intracellular Growth
Source: PLoS Pathog. 2013 Oct 3;9(10):e1003668. doi: 10.1371/journal.ppat.1003668 (PMC3789771; doi:10.1371/journal.ppat.1003668)

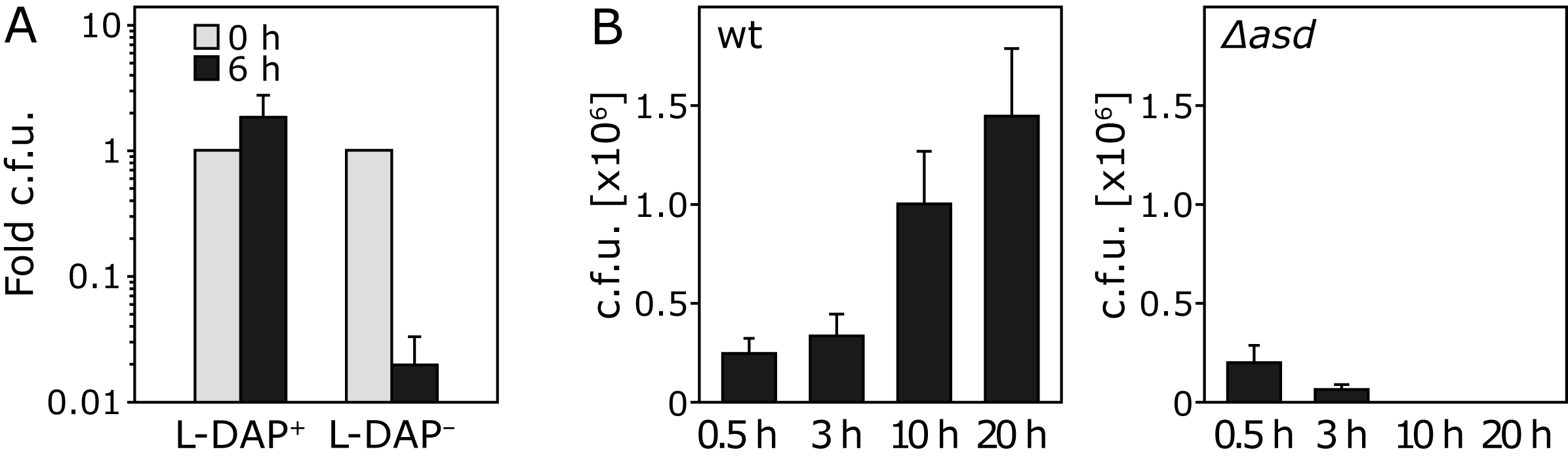

Supplement: Figure S1 — SPI-1 T3SS activity, uptake and intracellular survival of the S. Typhimurium Δasd mutant. (A) The SPI-1 T3SS of the S. Typhimurium Δasd mutant looses its activity upon withdrawal of L-DAP. The S. Typhimurium Δasd mutant was grown in the absence of L-DAP for 0 or 6 hs and its ability to enter into cultured epithelial cells (a very sensitive functional readout of SPI-1 T3SS function) was evaluated by the gentamicine protection assay after plating in the presence of L-DAP (see Methods). Invasion values are expressed relative to those obtained after growth of the strain in the presence of L-DAP, which were considered 1 and are the mean ± standard deviation of three repetitions. (B) Uptake and intracellular survival of the S. Typhimurium Δasd mutant. The SPI-1 T3SS Henle-407 cells were infected (MOI = 5) with wild-type S. Typhimurium or the isogenic Δasd mutant derivative for 1 h and chased in the presence of gentamicin. At the indicated times cells were lysed, bacteria released, plated (in the case of the Δasd mutant in the presence of L-DAP), and the number of colony forming units determined. Depicted are the mean values (± SEM) of three independent experiments. The detection limit for this experiment was ∼10 c. f. u. (TIF) [file ppat.1003668.s001.tif]

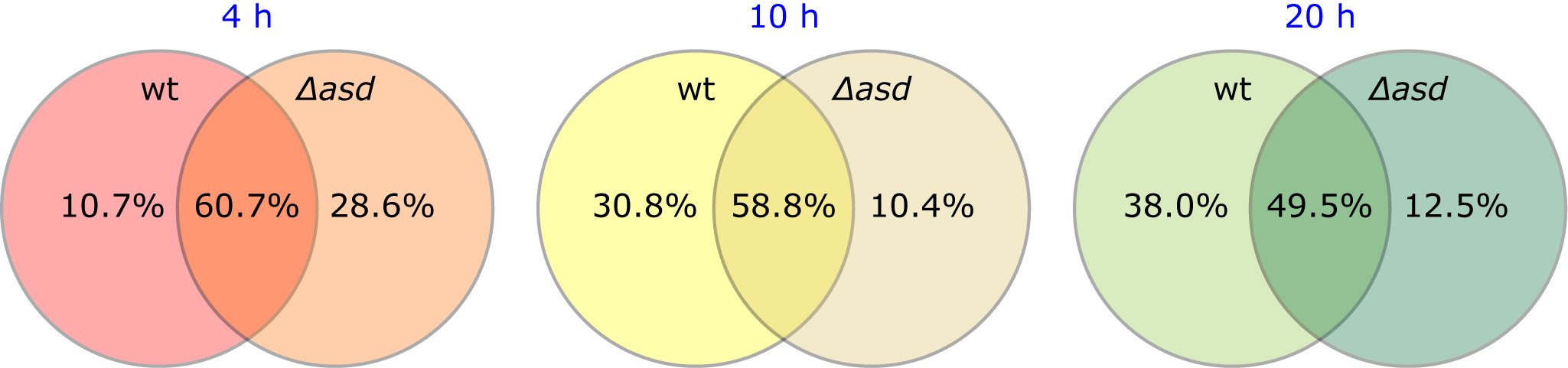

Supplement: Figure S2 — Venn diagram depicting the number of unique and common genes whose expression changed at least 5 fold at the indicated times after infection of Henle-407 cells with wild type S. Typhimurium or the isogenic Δasd mutant. (TIF) [file ppat.1003668.s002.tif]

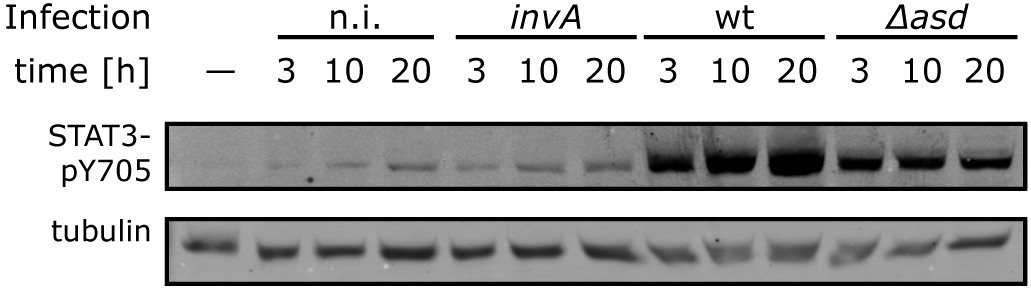

Supplement: Figure S3 — The S. Typhimurium Δasd mutant induces STAT3 activation. HeLa cells were infected (MOI = 10) with wild-type S. Typhimurium or the isogenic ΔinvA (T3SS-defective) or Δasd mutants for 1 h. Following chase in gentamicin containing medium, cells were lysed at the indicated times, separated by SDS-PAGE and probed by immuno blotting with antibodies to the phosphorylated (activated) form of STAT3 (P-Y705) and tubulin (loading control). (n. i.: not infected). (TIF) [file ppat.1003668.s003.tif]

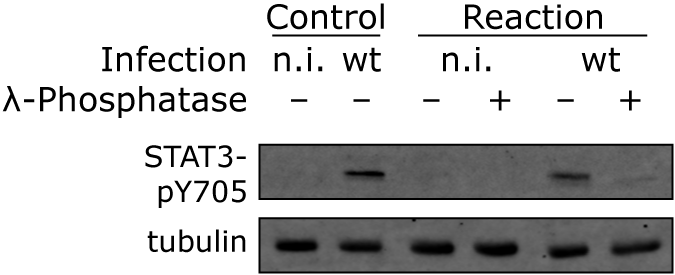

Supplement: Figure S4 — Phosphatase treatment eliminates the reactivity of the antibody directed to phosphorylated STAT3. Henle-407 cells were infected (MOI = 10) with wild-type S. Typhimurium for 1 h. Following chase in gentamicin containing medium for 3 hs, cells were lysed, separated by SDS-PAGE and probed by immuno blotting with antibodies to the phosphorylated (activated) form of STAT3 (P-Y705), and tubulin (loading control). When indicated, samples were treated with λ-phosphatase for 30 minutes prior to loading. (n. i.: not infected). (TIF) [file ppat.1003668.s004.tif]

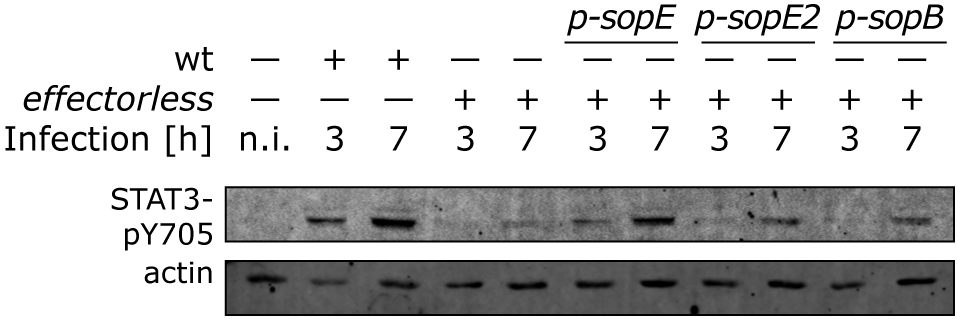

Supplement: Figure S5 — Salmonella stimulation of transcriptional responses in infected cells requires the SPI-1 T3SS effectors SopE, SopE2, and SopB. Henle-407 cells were infected (MOI = 10) for 1 h with wild-type S. Typhimurium, a mutants defective in all known effectors of the SPI-1 T3SS (effectorless), or the effectorless mutant complemented with plasmid-borne wild type alleles of sopE, sopE2, or sopB, as indicated. Following chase in gentamicin containing medium, cells were lysed at the indicated times, separated by SDS-PAGE and probed by immuno blotting with antibodies to the phosphorylated (activated) form of STAT3 (P-Y705), and actin (loading control). (TIF) [file ppat.1003668.s005.tif]

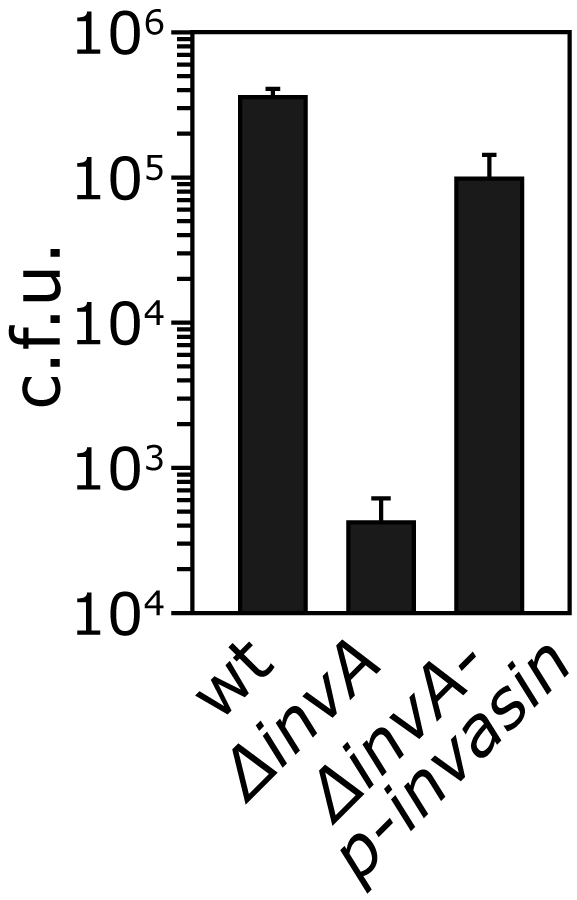

Supplement: Figure S6 — Invasin-mediated internalization of SPI-1 T3SS-defective S. Typhimurium. Henle-407 cells were infected (MOI = 10) with the indicated strains of S. Typhimurium for 1 h. Following chase in gentamicin containing medium for 2 h cells were lysed and c. f. u. enumerated by plating dilutions of the bacterial suspension. Values represent the mean (± SD) of three independent measurements. (TIF) [file ppat.1003668.s006.tif]

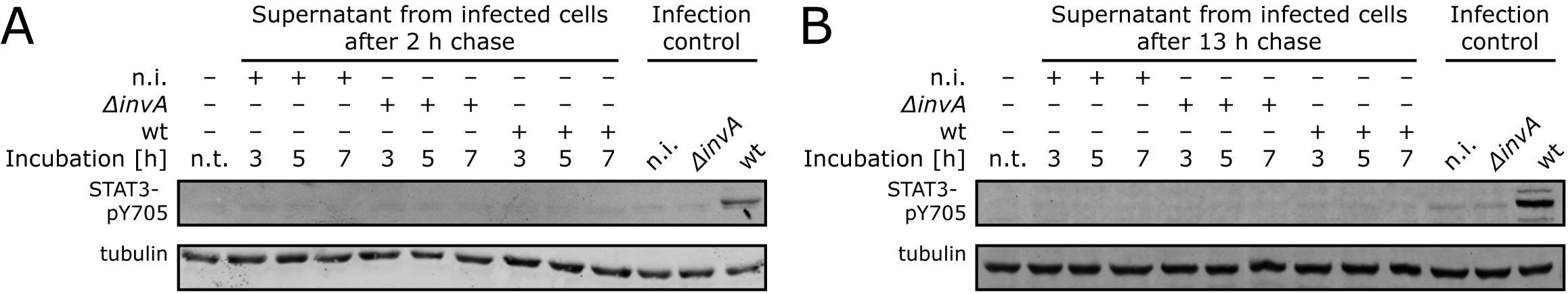

Supplement: Figure S7 — Culture supernatants from Henle-407 infected cells do not activate STAT3. Culture supernatants were obtained from Henle-407 cells 2 or 13 h after infection (MOI = 10) with either wild-type S. Typhimurium or the SPI-1 T3SS-defective ΔinvA mutant, filtered sterilized, and applied to uninfected Henle-407 cells (A and B panels, respectively). At different times after treatment cells were lysed, separated by SDS-PAGE and probed by immuno blotting with antibodies to the phosphorylated (activated) form of STAT3 (P-Y705), and tubulin (loading control). As a control, infected cells were analyzed for STAT3 activation in a similar fashion. (TIF) [file ppat.1003668.s007.tif]

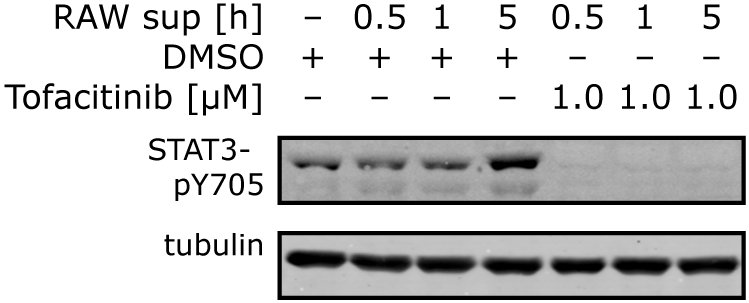

Supplement: Figure S8 — Effectiveness of the JAK inhibitor Tofacitinib. HepG2 cells (pretreated with 1.0 µM Tofacitinib or DMSO were incubated for the indicated periods with supernatant from activated RAW macrophages in the presence of the inhibitor or DMSO. Cell lysates were applied to SDS-PAGE and immuno blotting. (TIF) [file ppat.1003668.s008.tif]

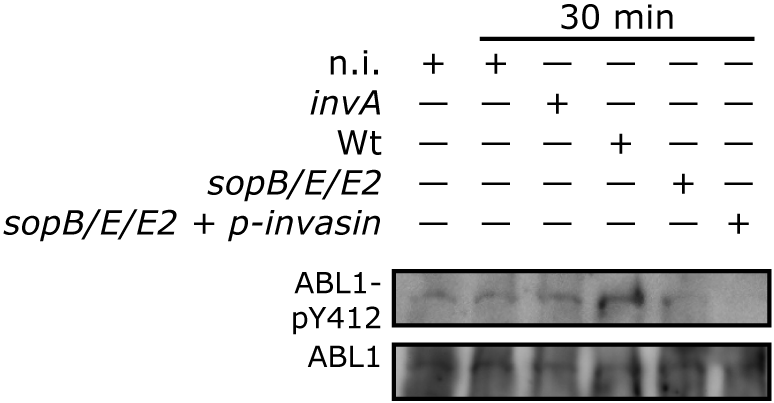

Supplement: Figure S9 — Salmonella activates ABL1 in a sopB/sopE/sopE2-dependent manner. Cultured HEK-293T cells were infected (MOI = 30) with the indicated strains of S. Typhimurium for 30 min in HBSS. Cells were lysed, separated by SDS-PAGE and probed by immuno blotting with antibodies to the phosphorylated (activated) form of ABL1 (P-Y412) and total ABL1 as a loading control (n.i.: non infected). (TIF) [file ppat.1003668.s009.tif]

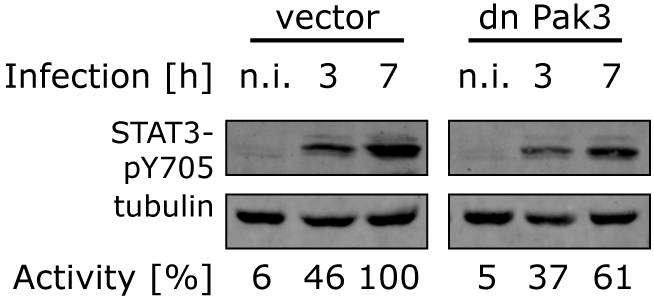

Supplement: Figure S10 — Expression of dominant negative Pak3 reduces Salmonella induced activation of STAT3. Cultured Henle-407 cells were thansfected with a plasmid encoding dominant negative Pak3 or the vector control. Transfected cells were subsequently infected (MOI = 10) with wild-type S. Typhimurium for 1 h and chased in the presence of gentamicin. At the indicated times cells were lysed, separated by SDS-PAGE and probed by immuno blotting with antibodies to the phosphorylated (activated) form of STAT3 (P-Y705), and tubulin (loading control). The relative levels of STAT3 activation in the infected cells were calculated after quantification with the Odyssey LI-COR system and are expressed relative to the phospho-STAT3 signal in the control sample 6 h after infection. (TIF) [file ppat.1003668.s010.tif]

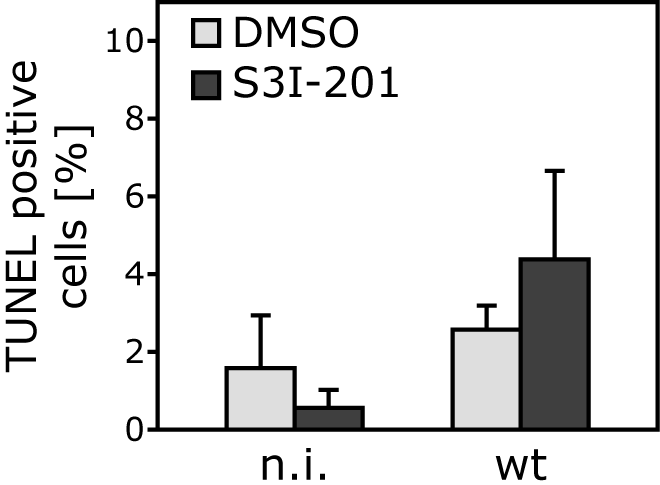

Supplement: Figure S11 — S. Typhimuirum infection of cultured cells in the presence of a STAT3 inhibitor does not result in significant increase of apoptosis. Henle-407 cells were infected for 1 h with S. Typhimurium (m. o. i. 5) in the presence of the STAT3 inhibitor S31-201 or DMSO and the percentage of cells undergoing apoptosis was determined 9 hs after infection by TUNEL statining. Notice that greater than 60% of cells were infected in this experiment and therefore the minor increase in TUNEL positive cells observed overall, which was not statistically significant, is of no biological relevance. (TIF) [file ppat.1003668.s011.tif]

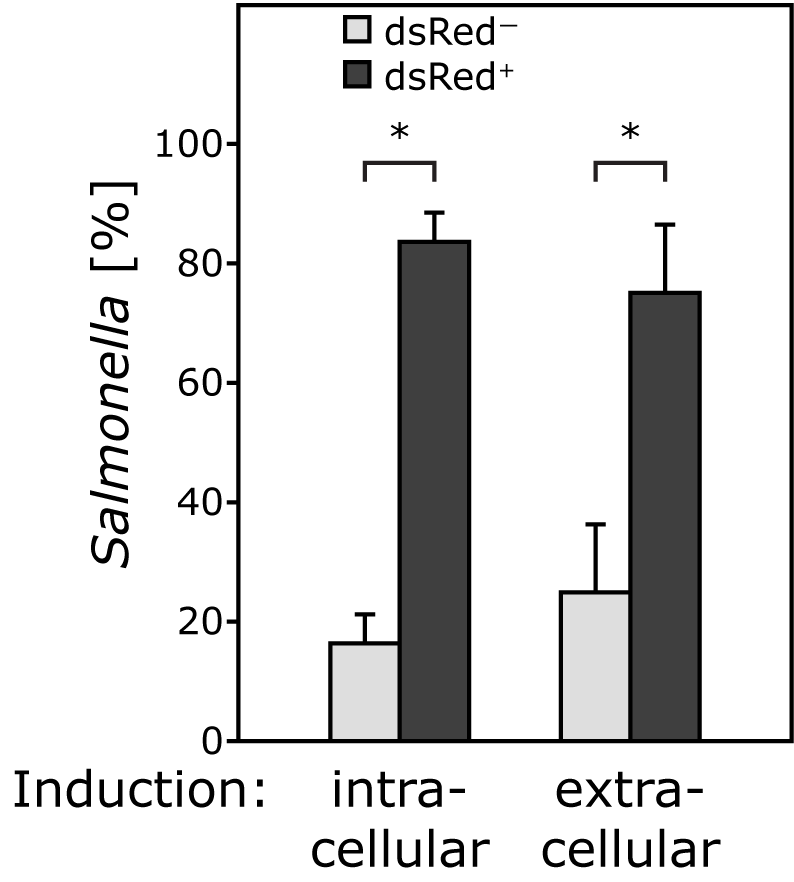

Supplement: Figure S12 — Arabinose-induced expression of dsRed in S. Typhimurium after their release from intracellular compartments. Henle-407 cells were infected for (MOI = 10) 1 h with S. Typhimurium expressing dsRed under the control of an arabinose-inducible promoter. After 17 h chase in gentamicin supplemented medium cells were incubated for additional 3 h in the presence of 0.1% arabinose to induce dsRed expression. Alternatively, bacterial cells were released, transferred into a test tube containing HBSS and 0.1% arabinose and incubated for 3 h at 37°C. Bacterial cells were fixed, immuno stained for LPS, and analyzed by epifluorescence microscopy to determine the percentage of bacteria expressing dsRed. Numbers are the percentages of dsRed positive and negative bacteria and represent the means (± SD) of three independent experiments in which at least 100 bacteria were quantified. *: indicates statistically significant differences (p≤0.03). (TIF) [file ppat.1003668.s012.tif]

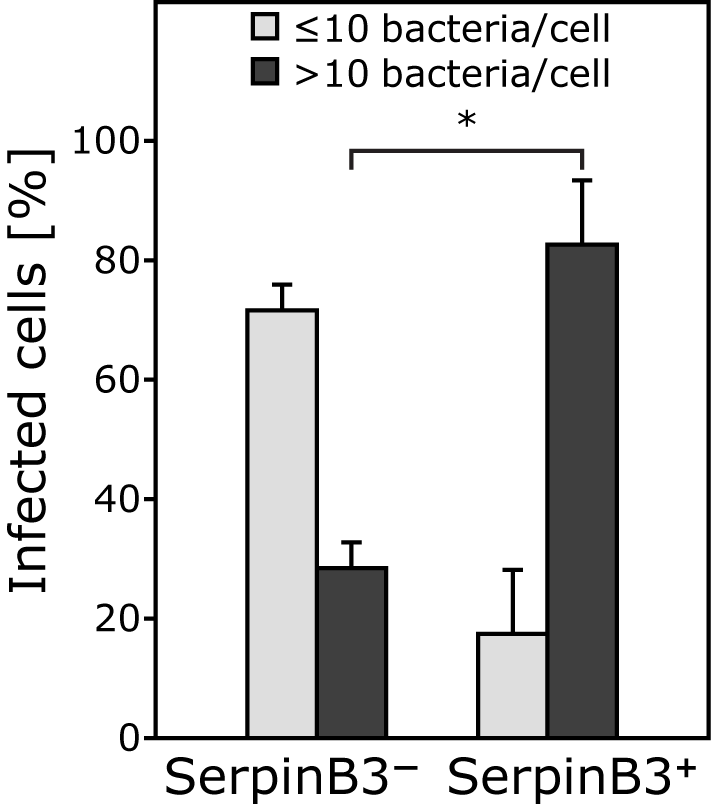

Supplement: Figure S13 — Host cell gene expression reprogramming is required for efficient Salmonella intracellular replication in HeLa cells. HeLa cells were infected (MOI = 10) for 1 h with S. Typhimurium chased for 20 h in gentamicin supplemented medium, fixed, immuno stained for LPS (to stain for Salmonella), endogenous SerpinB3 and DNA, and the total number of bacteria in SerpinB3-positive or negative cells were enumerated by epifluorescence microscopy. Values are the means (± SD) of the percentages of SerpinB3-positive or negative cells that had a bacterial load of up to 10 bacteria or more than 10 bacteria, and represent three independent experiments in which at least 100 cells per bacterial strain were examined. *: indicates statistically significant differences (p≤0.003). (TIF) [file ppat.1003668.s013.tif]

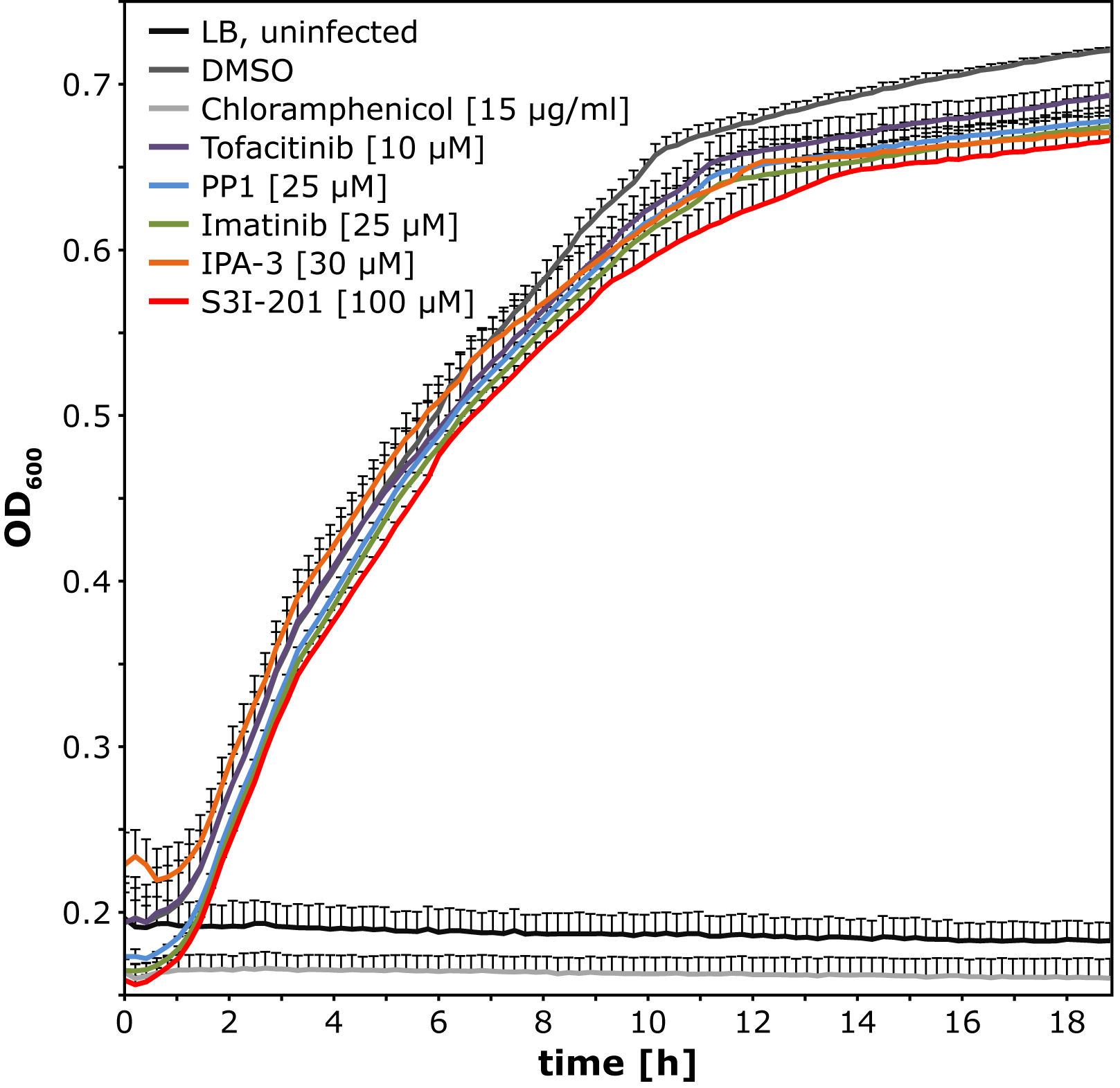

Supplement: Figure S14 — Effect of chemical inhibitors on S. Typhimurium growth. S. Typhimurium was cultured on LB containing the indicated inhibitors. Bacterial growth was monitored every 10 min at an OD600 for more than 18 h at 37°C. Depicted are the mean values (+ SD) of three technical replicates. (TIF) [file ppat.1003668.s014.tif]
