# Supplementary material for: Salmonella Modulation of Host Cell Gene Expression Promotes Its Intracellular Growth
Source: PLoS Pathog. 2013 Oct 3;9(10):e1003668. doi: 10.1371/journal.ppat.1003668 (PMC3789771; doi:10.1371/journal.ppat.1003668)
Supplement: Table S1 — Microarray analysis of the transcriptional responses induced by different strains of Salmonella Typhimurium. Depicted are alphabetically sorted genes upregulated at least 3-fold after infection in at least one condition. (PDF) [file ppat.1003668.s015.pdf]

Table S1: Microarray analysis of the transcriptional responses induced by different strains of *Salmonella* Typhimurium. Depicted are alphabetically sorted genes upregulated at least 3-fold after infection in at least one condition.

				4 h					10 h					20 h		
**Probeset**	**GeneID**	***invA***	***invA***	**wt**	**wt**	***Δasd***	***invA***	***invA***	**wt**	**wt**	***Δasd***	***invA***	***invA***	**wt**	**wt**	***Δasd***
203504_s_at	ABCA1	0.9	1.0	0.8	0.7	0.7	1.0	1.2	12.1	10.8	8.8	0.9	1.2	40.5	22.3	12.9
204567_s_at	ABCG1	1.1	1.2	1.0	1.0	1.0	0.7	1.1	2.5	2.1	1.7	0.8	0.9	9.7	2.7	3.2
204393_s_at	ACPP	1.0	1.0	1.5	1.1	0.9	1.0	1.2	3.1	2.2	2.2	0.9	1.1	8.1	3.6	3.4
1557418_at	ACSL4	0.9	1.0	3.6	3.0	2.9	1.2	0.9	2.3	2.2	2.0	1.1	1.1	1.4	1.6	2.1
1557419_a_at	ACSL4	0.9	1.3	3.3	3.6	3.6	0.9	1.0	2.0	2.1	1.8	1.1	0.9	1.4	1.4	1.6
209765_at	ADAM19	1.1	1.1	1.3	1.3	1.0	1.0	1.0	2.2	1.8	1.4	1.2	1.0	4.6	1.8	1.5
229004_at	ADAMTS15	0.9	1.1	3.0	2.2	2.6	1.1	0.9	3.8	3.5	2.6	1.1	1.2	5.0	3.8	3.1
220578_at	ADAMTSL4	1.0	0.9	2.1	1.6	1.9	0.7	0.9	3.9	3.8	3.8	1.0	1.0	3.6	3.6	3.7
202912_at	ADM	0.9	1.1	6.0	6.4	7.3	1.0	1.1	1.6	2.7	2.4	1.1	1.1	1.9	5.4	2.7
206170_at	ADRB2	0.7	1.5	3.5	4.3	5.1	0.9	1.3	4.2	4.0	2.6	1.5	1.0	4.5	2.7	2.6
226955_at	AFAP1L1	0.9	1.0	0.9	1.1	0.9	1.3	1.1	4.2	3.4	2.9	0.9	0.9	3.4	4.0	2.6
202834_at	AGT	0.9	1.1	2.0	1.7	1.7	0.8	1.0	18.5	36.5	25.8	1.2	0.9	58.3	31.7	29.4
212543_at	AIM1	1.3	1.1	5.6	2.0	2.7	0.8	1.4	11.7	10.3	7.6	0.9	0.8	25.6	8.2	3.2
206513_at	AIM2	0.9	1.2	1.0	1.4	1.1	0.8	0.8	2.0	1.2	1.2	1.0	1.0	19.0	6.5	11.2
225342_at	AK3L1	0.9	0.9	1.4	1.3	1.3	0.8	1.0	2.3	2.7	1.7	1.0	1.1	2.9	3.8	1.9
210517_s_at	AKAP12	1.0	1.4	3.1	3.8	4.4	1.0	0.9	3.7	4.9	4.5	1.0	0.9	3.9	5.3	1.8
231067_s_at	AKAP12	0.8	1.2	3.7	2.9	4.3	0.8	1.0	4.2	3.9	2.6	0.9	0.7	2.8	2.8	1.0
221009_s_at	ANGPTL4	1.0	0.6	1.4	1.2	1.9	1.0	1.0	3.2	3.4	2.4	1.0	1.0	2.6	3.5	2.2
206385_s_at	ANK3	0.9	1.1	0.9	1.3	1.3	0.9	1.3	1.4	1.0	1.0	0.9	0.9	5.9	2.9	1.9
238439_at	ANKRD22	1.0	0.9	1.1	0.9	1.0	1.0	0.9	4.9	2.7	1.8	1.0	1.3	24.4	7.8	3.0
227337_at	ANKRD37	0.8	1.0	2.7	4.7	4.3	1.0	0.8	2.3	2.2	1.2	1.0	1.0	1.7	2.8	1.9
227034_at	ANKRD57	1.0	1.0	4.2	3.4	3.1	1.0	1.0	4.0	4.2	3.8	1.0	1.0	2.1	2.1	2.5
225524_at	ANTXR2	0.7	1.1	0.7	0.8	0.8	1.0	1.4	5.4	5.1	3.3	0.9	1.0	16.4	8.1	6.1
233011_at	ANXA1	1.0	1.4	3.0	2.6	3.3	1.3	0.9	2.9	2.2	1.6	1.1	1.1	3.6	2.8	3.6
209546_s_at	APOL1	1.0	0.9	1.1	1.0	1.2	1.0	1.1	2.7	2.0	2.6	1.0	1.1	5.3	2.3	3.1
221653_x_at	APOL2	2.0	1.7	1.6	1.3	2.0	1.1	1.0	5.1	4.9	4.6	1.0	1.0	6.1	5.1	2.3
1557236_at	APOL6	1.5	1.7	2.8	1.4	1.7	0.9	1.0	13.3	7.6	6.6	1.1	0.7	8.5	2.3	1.4
205239_at	AREG	0.8	1.1	17.5	18.7	16.5	1.1	1.0	12.4	8.2	10.8	1.0	1.0	3.3	2.6	6.6
1557285_at	AREGB	1.0	0.9	4.0	2.6	1.8	0.9	0.9	2.0	1.8	1.5	1.1	1.1	1.3	0.8	1.1
205020_s_at	ARL4A	1.0	1.2	1.6	1.4	1.7	0.9	1.1	2.7	2.4	2.0	1.0	1.1	5.2	4.5	2.8
223586_at	ARNTL2	1.3	1.4	1.9	1.5	1.5	1.0	1.0	6.7	6.4	5.8	1.1	1.4	5.5	5.5	6.4
224797_at	ARRDC3	1.0	0.9	3.2	4.8	4.2	1.2	1.4	5.0	7.2	5.4	0.9	1.2	4.2	4.7	3.6
202672_s_at	ATF3	1.0	0.8	6.3	7.5	7.1	0.9	0.8	2.6	2.4	1.6	0.9	1.0	2.6	2.2	0.9
213238_at	ATP10D	1.1	1.0	2.3	1.4	1.3	1.1	1.3	4.5	3.8	4.2	1.2	1.2	3.5	2.5	2.9
212135_s_at	ATP2B4	1.3	1.0	1.0	0.8	1.2	1.2	0.9	1.7	1.9	1.5	1.1	1.0	3.5	2.8	1.9
220416_at	ATP8B4	1.0	0.9	1.0	1.1	0.8	1.0	1.0	9.0	6.8	4.9	1.0	1.0	45.9	16.9	8.1
221485_at	B4GALT5	1.0	1.2	2.1	2.0	2.3	1.1	1.1	3.3	3.0	2.6	1.0	1.0	1.9	1.8	1.6
220358_at	BATF3	1.0	1.2	2.6	1.8	1.9	1.0	1.1	7.5	6.3	4.6	1.0	1.0	7.0	3.8	1.9
1557257_at	BCL10	1.1	1.4	2.5	4.6	3.8	0.9	1.4	1.0	1.6	1.4	1.0	1.1	0.7	1.3	1.3
204908_s_at	BCL3	1.0	1.1	3.6	2.9	3.2	1.3	1.1	5.0	4.7	4.4	1.2	0.9	3.7	2.6	3.3
228758_at	BCL6	0.9	0.9	2.4	2.0	2.2	0.9	1.3	5.6	7.0	6.1	0.9	1.0	6.1	6.7	4.2
202315_s_at	BCR	1.0	1.1	1.4	1.2	1.2	1.0	0.9	3.9	4.0	3.2	1.0	0.9	4.4	3.3	2.8
226602_s_at	BCR	1.1	0.9	1.3	1.3	1.3	1.3	0.9	4.3	3.7	2.3	1.0	0.9	3.0	2.1	1.6
206382_s_at	BDNF	0.8	1.2	1.7	1.3	1.5	1.1	1.0	2.8	2.3	1.6	0.9	1.1	4.1	2.7	1.1
201170_s_at	BHLHE40	1.0	0.7	3.4	3.3	2.7	1.1	1.0	4.2	5.3	3.5	1.0	1.0	4.1	3.7	2.8
210538_s_at	BIRC3	2.4	3.0	7.5	6.8	10.4	1.0	1.0	4.3	3.8	5.0	1.0	1.3	3.1	5.3	4.2
201848_s_at	BNIP3	1.0	1.0	0.8	0.9	0.9	1.1	1.0	1.3	1.6	1.0	0.9	1.0	3.1	4.8	2.4
209183_s_at	C10orf10	0.7	0.9	1.0	1.6	2.6	0.8	1.4	3.5	5.7	2.5	1.0	0.9	16.0	8.0	1.2
227099_s_at	C11orf96	0.9	0.9	2.3	2.1	4.7	1.2	0.9	12.6	9.5	2.1	1.0	0.8	166.4	117.6	13.0
1555786_s_at	C14orf34	1.1	1.2	1.8	1.6	1.7	0.8	0.9	4.3	3.7	2.4	1.1	1.0	9.1	5.8	5.0
242649_x_at	C15orf21	2.0	2.5	1.7	1.5	1.8	1.5	0.9	3.1	3.9	2.9	1.1	1.2	8.5	5.5	2.9
1559125_at	C16orf23	0.9	1.0	1.4	1.0	1.1	1.1	1.1	2.5	1.4	1.3	0.9	0.9	5.6	1.8	1.4
218130_at	C17orf62	0.9	1.0	1.2	0.9	1.0	1.0	1.3	2.8	2.7	2.2	1.1	1.1	4.5	3.7	2.9
214696_at	C17orf91	1.0	0.9	3.1	2.9	2.9	1.0	1.0	3.7	2.8	2.4	1.1	1.1	4.6	3.4	3.1
1553333_at	C1orf161	1.0	1.0	1.3	1.0	1.0	1.2	1.0	1.8	1.3	1.1	1.2	0.9	3.8	3.6	1.0
227019_at	C1orf226	0.9	0.8	2.1	1.1	1.2	1.1	0.9	3.5	2.8	2.2	0.9	0.9	2.4	1.7	1.9
212067_s_at	C1R	1.1	1.2	0.9	1.0	1.3	1.0	0.9	3.4	2.6	1.9	1.1	1.1	11.5	4.3	4.8
208747_s_at	C1S	0.9	1.0	1.1	1.0	1.0	0.9	1.0	2.1	1.9	2.0	1.1	1.2	6.7	4.2	6.1
202357_s_at	C2/CFB	1.8	2.1	1.8	1.8	3.1	1.7	1.9	6.5	8.1	8.3	1.8	1.3	21.7	12.4	16.8
219474_at	C3orf52	1.2	1.4	3.9	3.7	3.9	1.1	0.9	3.1	2.4	1.9	0.9	1.1	1.5	1.3	1.3
236915_at	C4orf47	1.1	0.9	0.9	1.2	1.0	1.2	1.0	1.1	1.2	0.9	0.9	0.9	2.1	4.5	1.3
230405_at	C5orf56	1.6	1.4	1.2	1.1	1.2	0.9	0.9	2.9	2.9	2.3	1.1	0.9	4.2	2.3	1.4
227598_at	C7orf29	1.3	1.4	0.8	0.9	1.0	1.0	1.2	1.6	2.1	1.5	1.0	1.1	3.8	2.7	1.9
218541_s_at	C8orf4	0.9	1.0	4.6	2.7	5.0	0.8	1.0	2.3	2.7	3.5	0.8	0.9	0.9	1.5	1.2
227443_at	C9orf150	0.9	0.9	1.7	1.0	1.3	0.9	1.0	4.6	3.9	2.9	1.2	1.4	10.0	6.7	6.2
233504_at	C9orf84	1.0	0.9	1.4	1.1	1.1	1.0	0.9	4.8	1.8	1.4	1.1	1.1	3.2	1.7	1.8
203963_at	CA12	1.3	1.5	2.6	2.3	1.9	0.9	1.1	6.3	6.1	5.4	1.1	1.1	16.9	12.1	13.2
231270_at	CA13	1.2	0.8	8.6	4.0	4.0	1.0	1.3	13.6	17.1	13.7	1.1	1.2	5.8	5.5	3.2
205199_at	CA9	0.9	0.8	0.9	1.0	1.0	1.0	1.0	1.0	1.5	0.9	1.1	0.9	2.2	6.7	2.0
1552701_a_at	CARD16	1.3	0.9	1.2	1.0	1.3	1.3	1.1	3.5	2.8	2.2	1.0	1.4	4.0	4.7	6.6
1552703_s_at	CARD16/CASP1	1.4	1.3	1.1	1.0	1.2	1.8	1.3	4.8	3.7	4.2	1.0	1.2	2.7	3.1	5.2
211367_s_at	CASP1	1.4	1.3	0.9	1.0	0.9	1.5	1.2	4.5	3.6	3.5	0.9	1.3	2.8	3.3	5.1
209310_s_at	CASP4	1.0	1.0	1.2	1.2	1.3	1.1	1.1	3.3	3.3	3.0	1.0	1.2	2.0	2.4	2.8
216598_s_at	CCL2	1.1	1.5	41.5	46.1	57.5	0.9	1.2	121.0	182.4	139.6	1.7	1.4	80.5	36.4	17.6
205476_at	CCL20	3.6	5.6	33.2	35.2	64.4	1.3	0.9	10.0	10.1	12.8	0.7	1.3	1.2	1.8	2.3
1405_i_at	CCL5	1.1	1.1	1.2	1.1	1.4	1.1	1.0	1.6	1.4	3.7	1.0	1.1	3.6	3.0	5.2
227458_at	CD274	0.9	1.4	3.0	3.3	5.5	0.7	0.8	8.9	7.3	7.0	1.0	0.8	18.5	8.8	6.9
217523_at	CD44	1.0	1.3	2.5	3.1	3.1	1.1	1.2	3.6	3.0	2.4	1.1	1.2	5.5	4.1	2.5
228748_at	CD59	1.1	1.2	1.9	1.8	1.8	0.9	1.0	3.3	3.4	2.0	0.7	1.3	2.7	1.5	2.0
214721_x_at	CDC42EP4	1.0	1.2	1.9	1.6	1.6	1.2	0.9	3.2	3.3	2.3	1.1	1.0	2.5	1.7	1.9
218451_at	CDCP1	1.1	1.3	3.1	2.2	2.7	1.0	1.1	4.6	4.6	4.0	0.9	1.1	3.4	3.9	4.3
204677_at	CDH5	2.4	3.2	4.6	6.7	7.7	1.2	0.9	1.9	2.4	2.1	1.0	1.1	1.0	1.0	0.9
202284_s_at	CDKN1A	0.8	1.0	2.4	3.6	3.8	1.1	1.0	2.1	2.0	1.6	1.0	0.9	2.9	2.2	2.1
209498_at	CEACAM1	1.1	1.2	1.7	1.2	1.3	0.9	1.0	19.9	12.9	10.9	1.0	1.0	35.0	28.4	35.7
213006_at	CEBPD	1.0	1.5	16.9	18.7	20.8	1.2	1.2	11.0	9.2	5.7	0.9	0.9	7.3	5.3	6.9
219634_at	CHST11	1.3	0.9	1.3	1.0	1.1	1.0	0.8	5.8	4.7	4.1	0.9	0.9	8.2	3.6	4.0
223961_s_at	CISH	1.1	0.8	1.5	1.1	1.2	1.1	1.6	1.9	3.0	2.2	1.4	0.9	4.3	2.1	1.0
209732_at	CLEC2B	1.1	0.9	0.9	0.9	0.8	1.0	0.9	2.5	2.6	1.9	0.9	1.0	7.3	11.7	7.2
1558924_s_at	CLIP1	0.6	2.1	1.7	1.6	3.1	0.7	1.5	4.5	3.0	2.0	0.9	1.2	3.2	2.2	1.3

				4 h					10 h					20 h		
**Probeset**	**GeneID**	***invA***	***invA***	**wt**	**wt**	***Δasd***	***invA***	***invA***	**wt**	**wt**	***Δasd***	***invA***	***invA***	**wt**	**wt**	***Δasd***
226751_at	CNRIP1	1.0	1.0	0.9	1.0	0.8	1.1	1.1	2.4	2.2	1.5	1.0	1.1	3.6	3.0	2.8
219400_at	CNTNAP1	1.2	1.2	1.2	1.2	1.1	0.9	0.8	2.2	1.7	1.1	1.0	1.0	4.4	3.3	1.9
207630_s_at	CREM	1.1	1.2	3.3	3.1	3.1	1.1	0.9	3.6	3.7	3.1	0.9	0.8	2.5	1.8	2.6
221541_at	CRISPLD2	1.0	1.6	4.3	4.5	4.2	0.8	0.9	7.3	5.4	4.5	1.1	0.9	7.6	2.3	1.8
225557_at	CSRNP1	1.0	0.9	8.5	7.0	6.0	1.0	1.0	8.0	7.0	5.4	1.2	1.1	5.9	3.5	4.7
209101_at	CTGF	0.9	0.8	5.7	2.0	1.7	1.0	0.8	1.3	1.1	1.2	1.1	1.3	1.1	1.3	1.2
213275_x_at	CTSB	0.8	1.0	1.2	1.0	1.1	1.1	1.2	6.2	7.5	5.8	1.0	1.0	14.7	14.0	12.6
202087_s_at	CTSL1	1.0	1.1	1.3	1.2	1.2	1.0	1.0	2.5	2.2	1.9	1.0	1.0	3.6	2.6	2.8
1563445_x_at	CTSLL3	1.1	0.8	0.9	0.9	0.8	1.6	1.1	2.6	2.7	2.4	0.8	0.9	4.1	2.8	4.2
202902_s_at	CTSS	2.6	2.1	1.5	1.0	1.6	3.2	2.3	4.1	3.5	5.5	1.5	1.8	6.3	6.6	9.0
209774_x_at	CXCL2	1.3	1.3	67.8	37.0	38.3	1.1	0.9	44.2	38.1	36.0	1.3	0.8	17.5	8.9	15.1
207850_at	CXCL3	1.7	1.3	22.6	10.4	14.1	1.1	0.9	22.5	18.4	17.5	0.9	1.0	5.7	3.6	11.5
202437_s_at	CYP1B1	1.0	3.8	0.7	4.0	4.1	1.8	1.4	3.7	3.3	3.7	1.1	1.5	5.6	2.9	2.8
210764_s_at	CYR61	1.4	0.9	4.8	2.8	3.1	1.4	1.0	1.2	1.2	1.6	1.1	1.2	1.8	2.4	1.9
203139_at	DAPK1	1.0	1.3	0.4	0.9	0.9	1.0	1.3	2.0	2.1	1.8	1.0	1.2	8.0	4.5	3.6
239648_at	DCUN1D3	1.1	0.9	2.0	1.9	1.9	1.1	0.9	4.1	3.2	2.7	1.0	1.1	3.7	2.9	2.4
218943_s_at	DDX58	1.3	1.3	1.0	1.0	1.5	1.2	1.2	8.8	5.5	15.1	1.2	1.0	7.7	6.4	8.4
206090_s_at	DISC1	2.1	1.9	3.5	2.4	2.5	0.9	0.9	6.7	4.2	3.9	1.3	1.1	4.8	2.6	3.1
204602_at	DKK1	1.0	0.9	6.9	3.0	3.3	1.1	1.2	2.1	2.1	2.8	0.9	1.3	1.4	2.4	1.8
208086_s_at	DMD	0.9	1.2	1.7	1.2	1.0	0.9	0.9	2.4	4.1	3.2	0.9	1.0	1.6	2.3	3.2
243938_x_at	DNAH5	1.1	1.2	1.3	1.2	1.1	0.9	0.8	3.0	2.8	2.0	0.9	1.2	4.1	2.6	3.0
225415_at	DTX3L	1.8	1.6	1.8	1.3	1.5	1.1	1.2	3.9	3.8	4.6	1.0	1.1	4.0	2.6	3.1
201041_s_at	DUSP1	0.9	0.5	3.4	2.8	2.7	1.0	0.8	1.5	1.3	1.6	1.0	0.9	0.8	1.1	1.5
204014_at	DUSP4	0.8	1.1	3.1	3.2	4.6	0.9	1.1	1.7	2.3	2.5	0.9	1.1	0.9	1.8	1.4
209457_at	DUSP5	0.8	0.8	4.9	3.9	3.6	0.9	1.1	5.4	4.8	4.6	1.1	1.1	3.3	3.2	2.6
208892_s_at	DUSP6	0.7	1.2	4.9	4.5	4.4	1.2	1.1	8.7	12.2	12.5	0.8	1.2	3.4	7.1	9.6
225275_at	EDIL3	0.9	1.0	1.0	0.9	0.9	1.0	0.9	3.1	2.6	2.3	1.0	1.1	5.5	4.7	5.1
202023_at	EFNA1	1.1	1.0	2.1	1.4	1.6	1.0	0.9	3.2	3.1	2.0	1.0	1.0	3.9	3.6	1.9
202668_at	EFNB2	0.8	0.7	3.3	2.3	2.7	1.0	1.3	4.6	5.0	4.0	1.0	1.1	5.4	4.2	2.5
224314_s_at	EGLN1	1.1	1.5	1.6	1.9	1.8	1.4	0.9	2.6	2.9	1.9	1.1	1.2	2.8	4.1	2.2
201694_s_at	EGR1	1.0	1.2	21.0	30.3	29.5	1.0	1.2	0.9	1.5	1.4	1.3	1.3	1.7	1.7	1.5
207768_at	EGR4	1.0	1.2	4.3	4.4	3.8	1.0	0.9	1.3	1.2	1.2	1.0	0.8	1.2	1.2	0.9
209536_s_at	EHD4	1.0	1.5	2.1	2.2	2.4	1.1	0.9	3.7	2.1	2.2	0.9	1.1	4.3	3.7	2.1
229074_at	EHD4	0.9	1.5	2.5	2.1	2.5	0.9	1.0	4.8	4.8	4.1	1.2	0.7	7.1	4.0	2.4
231292_at	EID3	1.8	2.7	8.7	6.1	6.2	1.2	1.0	1.2	1.2	0.9	0.9	0.7	1.1	0.7	0.8
221773_at	ELK3	1.0	1.1	1.1	1.0	1.0	1.0	1.1	3.9	4.8	3.9	0.8	1.0	2.3	2.6	2.9
213712_at	ELOVL2	0.9	1.0	1.0	1.0	1.2	1.1	1.4	3.8	4.1	3.3	1.1	1.1	3.5	3.1	1.9
201324_at	EMP1	0.9	0.8	2.0	2.1	2.1	1.0	1.1	2.4	2.1	1.9	1.0	1.4	3.1	3.1	2.5
207610_s_at	EMR2	1.0	1.3	2.5	2.5	1.9	1.2	1.2	7.5	6.5	6.1	1.0	1.4	8.1	5.4	10.9
201341_at	ENC1	1.1	1.2	3.1	2.4	1.9	0.9	0.7	3.6	3.5	3.3	1.1	0.7	2.7	1.5	1.5
201313_at	ENO2	1.1	0.9	1.2	1.2	1.1	1.2	0.9	2.2	3.0	1.5	1.0	0.9	3.7	7.5	3.0
200878_at	EPAS1	1.0	1.0	2.1	1.2	1.4	1.0	1.0	8.6	6.4	4.9	1.0	1.0	5.8	4.9	4.0
203499_at	EPHA2	1.2	0.9	4.6	4.2	4.5	1.1	1.0	2.8	2.8	3.2	0.9	1.0	1.8	2.4	3.7
205767_at	EREG	1.1	1.4	6.4	7.5	8.1	1.3	1.6	8.7	9.2	14.0	1.1	1.4	7.5	7.7	22.0
218498_s_at	ERO1L	1.2	1.2	1.6	1.2	1.0	1.1	1.0	1.9	2.0	1.6	1.0	1.0	2.9	4.1	2.2
225750_at	ERO1L	1.2	1.2	1.5	1.2	1.2	1.1	0.9	2.3	2.5	1.6	1.1	1.0	3.0	4.2	2.1
224657_at	ERRFI1	0.9	0.9	4.0	3.4	3.3	1.1	1.0	2.4	2.2	1.9	1.1	1.1	1.5	2.5	1.7
208394_x_at	ESM1	1.0	0.8	1.3	1.1	0.9	1.0	0.8	4.4	2.7	2.0	0.9	0.7	3.6	1.2	1.7
225764_at	ETV6	1.2	1.2	1.5	1.2	1.1	1.2	1.1	4.0	3.4	2.7	1.1	1.2	3.4	2.4	2.1
213506_at	F2RL1	0.8	1.0	7.6	6.3	6.6	1.1	1.5	18.3	24.1	15.3	1.1	1.6	37.5	30.3	10.6
204363_at	F3	1.1	1.0	3.3	3.2	3.5	1.1	1.0	3.5	3.4	3.7	0.9	1.2	1.7	2.8	3.2
225436_at	FAM108C1	1.1	1.1	1.4	1.3	1.2	1.1	1.0	2.8	3.3	2.9	1.0	1.1	3.8	3.1	5.1
241031_at	FAM148A	1.1	1.3	1.6	1.4	1.6	0.9	1.0	3.8	2.4	2.0	1.1	1.4	2.5	1.5	2.0
238018_at	FAM150B	1.0	1.0	1.3	1.4	1.2	0.9	1.0	1.8	1.7	1.2	1.3	0.8	22.3	6.2	1.1
231880_at	FAM40B	0.8	1.1	2.7	2.3	2.4	1.0	1.4	4.5	5.4	3.6	1.0	0.7	3.5	1.8	2.1
226811_at	FAM46C	0.8	0.9	1.6	1.5	1.7	0.8	1.0	4.2	4.7	2.9	1.0	1.0	6.4	3.4	1.4
238460_at	FAM83A	1.0	1.2	2.2	1.6	1.7	1.1	1.0	6.2	6.2	4.4	1.0	1.0	4.4	4.1	4.0
203184_at	FBN2	1.0	0.7	1.0	0.8	0.8	1.1	0.8	3.9	2.9	2.4	1.0	1.1	4.2	3.8	4.2
205650_s_at	FGA	1.0	0.9	4.7	2.1	2.3	0.8	0.6	48.8	35.0	28.1	1.7	0.8	157.1	91.9	69.2
204988_at	FGB	0.7	1.2	12.3	2.8	3.7	0.9	1.1	322.0	215.3	195.2	3.6	0.9	677.9	351.5	378.9
206987_x_at	FGF18	1.0	0.9	6.7	4.0	2.6	1.1	1.2	1.1	1.2	1.1	1.1	1.0	1.1	0.8	0.9
219612_s_at	FGG	0.8	1.0	7.2	1.7	2.3	1.5	1.2	486.0	410.2	310.5	5.2	1.5	722.9	443.6	507.1
243309_at	FLJ27352	1.1	1.1	2.8	3.8	4.6	1.2	0.9	0.4	0.4	0.5	0.9	1.0	0.5	0.7	0.6
229521_at	FLJ36031	1.2	1.2	3.5	4.1	4.6	1.4	1.0	4.9	5.0	3.1	0.9	1.1	8.1	4.8	3.8
222853_at	FLRT3	1.1	1.8	5.2	5.0	5.5	0.9	1.1	11.8	9.6	8.4	1.3	1.3	26.0	13.4	13.1
226184_at	FMNL2	0.9	1.0	1.6	1.3	1.2	0.9	1.1	2.8	3.2	2.7	1.0	1.1	2.1	2.5	2.7
211719_x_at	FN1	1.0	1.6	1.2	1.3	1.1	0.9	0.9	3.7	2.3	2.1	1.0	1.1	2.9	1.8	2.2
229865_at	FNDC3B	1.1	1.6	1.9	1.7	2.2	1.3	1.5	4.1	3.9	4.3	1.0	1.4	4.6	4.4	4.6
209189_at	FOS	0.8	0.8	20.5	30.3	24.5	1.0	0.9	4.9	7.9	4.1	1.0	0.9	7.7	7.2	5.0
202768_at	FOSB	0.8	0.8	4.7	4.8	6.1	0.9	0.9	1.2	1.0	1.1	1.1	0.7	1.1	1.2	1.1
204420_at	FOSL1	0.9	1.3	5.2	3.6	4.2	1.0	0.9	3.6	3.4	3.6	1.0	1.0	2.1	2.9	2.6
227475_at	FOXQ1	1.0	1.4	3.6	3.4	3.3	0.9	0.9	3.6	2.4	2.7	0.9	1.0	1.7	1.6	2.0
213056_at	FRMD4B	0.7	1.2	9.2	6.4	7.0	1.2	1.0	13.5	8.7	5.5	0.8	1.0	14.0	12.8	8.2
225481_at	FRMD6	0.9	1.1	2.1	1.7	1.6	1.0	1.0	3.8	3.9	3.8	0.9	1.2	2.3	2.9	3.4
238551_at	FUT11	1.0	1.2	1.2	1.2	1.0	1.0	1.1	1.9	2.6	1.5	1.1	1.2	2.4	4.4	2.2
208869_s_at	GABARAPL1	1.0	1.0	1.2	1.5	1.6	1.1	1.2	2.4	2.3	2.1	0.9	1.1	3.7	2.8	2.2
211458_s_at	GABARAPL1/L3	1.1	1.2	1.7	1.6	2.1	1.0	0.9	2.2	2.1	1.7	1.0	1.0	3.9	3.0	2.1
207574_s_at	GADD45B	1.0	0.9	6.5	4.6	6.0	1.0	0.9	5.2	4.9	3.0	1.0	1.2	7.0	7.7	2.9
217787_s_at	GALNT2	1.3	1.1	1.5	1.3	1.4	1.3	0.9	2.9	2.3	2.2	1.0	0.9	4.2	2.7	3.0
223991_s_at	GALNT2	1.1	1.1	1.4	1.3	1.5	1.2	1.0	3.1	2.5	2.3	1.0	0.9	4.1	3.0	3.1
202269_x_at	GBP1	1.9	2.4	2.9	2.3	3.8	1.1	1.7	4.8	5.3	9.7	1.9	2.0	2.8	2.6	3.3
223434_at	GBP3	3.9	3.2	5.8	2.9	4.3	1.1	1.2	14.5	14.2	16.8	1.1	1.5	8.3	9.4	8.8
203765_at	GCA	0.8	1.2	0.7	1.1	1.0	1.1	1.1	3.7	2.9	3.2	1.0	1.3	5.3	2.8	2.5
219508_at	GCNT3	1.2	1.3	3.7	2.2	1.8	1.1	0.9	6.0	2.4	2.7	1.0	1.0	3.6	2.0	2.9
204472_at	GEM	1.1	0.8	21.1	19.2	18.5	0.8	1.1	2.3	2.2	1.9	0.8	0.8	4.7	2.2	2.0
205100_at	GFPT2	1.8	3.4	4.4	4.5	5.6	0.8	1.0	3.3	3.9	3.3	1.0	0.8	3.5	3.0	3.1
201667_at	GJA1	1.2	1.4	2.5	1.4	1.4	0.8	1.0	33.2	30.5	16.7	0.9	1.0	92.2	42.3	31.5
204222_s_at	GLIPR1	1.2	1.3	4.6	3.6	4.3	1.2	1.1	5.3	5.3	5.8	1.1	1.1	2.7	3.9	3.4
209276_s_at	GLRX	1.1	0.9	1.3	1.1	1.2	1.2	1.0	3.7	3.3	2.6	1.0	1.2	7.1	8.7	8.9
223079_s_at	GLS	1.1	1.6	3.5	4.2	2.8	1.3	0.8	3.4	2.6	2.7	1.2	1.4	2.6	2.8	3.6
213094_at	GPR126	0.9	0.9	3.4	1.5	1.6	1.0	1.0	16.7	13.9	11.3	1.0	1.1	15.7	11.0	12.1
219936_s_at	GPR87	1.1	1.1	6.0	4.4	4.9	1.0	1.3	1.7	2.3	2.0	1.1	0.9	2.3	1.7	2.6

				4 h					10 h					20 h		
**Probeset**	**GeneID**	***invA***	***invA***	**wt**	**wt**	***Δasd***	***invA***	***invA***	**wt**	**wt**	***Δasd***	***invA***	***invA***	**wt**	**wt**	***Δasd***
203108_at	GPRC5A	1.0	1.0	2.8	2.0	1.8	1.2	1.0	5.9	4.9	4.9	1.0	1.0	3.4	4.6	4.4
219327_s_at	GPRC5C	1.0	1.3	0.8	1.0	1.0	0.9	1.2	1.4	1.8	1.5	1.1	0.9	4.2	3.0	2.8
223541_at	HAS3	0.9	1.3	4.2	2.5	3.0	1.1	1.0	4.2	2.8	2.0	1.1	1.0	3.2	2.0	2.2
214414_x_at	HBA1/HBA2	1.0	0.7	1.9	0.8	1.4	0.9	0.8	2.8	2.6	2.2	0.9	1.0	12.3	15.0	15.7
213069_at	HEG1	1.0	0.9	1.9	1.2	1.2	1.0	1.2	16.4	15.8	13.0	1.1	1.1	21.5	13.2	20.5
232026_at	HERC4	0.8	1.2	3.2	3.9	2.5	1.0	1.0	1.1	1.0	1.1	0.9	1.0	0.8	0.9	1.0
203394_s_at	HES1	0.9	0.9	2.8	3.6	5.5	1.0	1.0	2.2	3.5	3.4	1.1	1.0	2.3	3.3	1.8
210148_at	HIPK3	1.2	1.1	1.2	1.3	1.1	1.4	0.9	3.3	2.3	1.6	0.9	1.0	5.7	2.2	1.6
212641_at	HIVEP2	0.9	1.4	1.6	1.4	1.7	1.2	0.8	4.2	2.9	2.0	1.3	1.2	4.6	3.8	2.1
200697_at	HK1	1.0	1.2	1.4	1.3	1.3	0.9	1.0	3.5	3.4	2.3	1.1	1.0	4.2	4.6	3.5
202934_at	HK2	1.1	0.9	3.6	2.7	2.7	1.0	0.9	7.6	7.6	5.8	0.9	1.1	5.3	6.1	5.6
200904_at	HLA-E	1.2	1.1	1.1	1.4	1.6	1.1	1.0	1.5	1.6	1.4	1.1	1.0	3.2	3.1	1.6
205579_at	HRH1	0.9	1.3	4.4	5.0	5.1	1.1	1.2	9.0	11.6	8.0	1.1	1.1	9.7	8.3	6.6
213418_at	HSPA6	1.1	1.1	4.6	2.2	1.8	1.1	1.0	3.6	2.9	2.0	1.0	1.0	4.9	2.9	1.6
216615_s_at	HTR3A	0.8	1.1	2.1	1.0	1.3	1.0	1.0	4.2	5.1	3.4	1.0	1.2	3.7	2.8	3.6
202638_s_at	ICAM1	1.9	1.6	4.6	2.8	5.3	1.4	1.1	5.1	6.1	4.2	1.3	0.7	4.6	2.3	1.5
208937_s_at	ID1	1.1	0.9	9.5	6.2	6.2	1.2	0.9	16.3	13.9	12.9	1.1	1.0	7.9	8.1	13.8
210029_at	IDO1	1.2	0.9	1.0	1.1	1.0	1.0	1.1	3.3	2.5	3.6	1.1	1.1	7.6	3.4	4.1
201631_s_at	IER3	1.0	0.9	4.6	4.0	4.0	1.1	0.9	9.6	6.3	6.1	1.2	1.0	7.0	7.1	7.5
208966_x_at	IFI16	1.0	0.9	3.3	2.3	2.4	0.9	1.0	6.1	6.5	6.3	1.1	1.0	4.3	3.4	4.3
209417_s_at	IFI35	1.3	1.2	0.9	1.0	0.9	1.3	1.4	1.9	2.0	2.4	1.1	0.9	4.8	2.9	3.2
214453_s_at	IFI44	1.3	1.1	0.9	0.9	1.4	1.4	1.5	5.4	4.2	20.2	0.9	1.1	4.2	4.5	10.1
219209_at	IFIH1	1.8	1.2	1.0	1.0	1.7	1.2	1.4	3.1	3.4	9.1	1.2	1.1	3.6	2.8	3.4
203153_at	IFIT1	1.0	1.1	0.9	0.9	6.2	1.1	1.3	8.7	7.9	13.8	1.1	1.3	2.4	3.4	5.4
226757_at	IFIT2	1.3	1.1	2.4	0.9	18.7	1.2	1.2	18.1	12.3	12.5	1.2	1.2	4.4	6.0	4.9
229450_at	IFIT3	1.8	1.6	1.9	1.0	5.3	1.2	1.3	11.2	10.1	11.5	1.0	1.2	7.6	6.6	6.0
214022_s_at	IFITM1	1.0	1.3	1.1	0.9	1.0	1.1	1.1	3.0	2.5	3.7	1.2	1.1	10.2	9.3	11.8
211676_s_at	IFNGR1	1.6	1.6	2.0	1.9	1.7	1.2	1.0	3.0	3.4	2.6	1.1	1.1	3.9	3.0	2.8
205302_at	IGFBP1	0.8	1.4	6.5	6.1	6.1	1.2	1.4	37.6	63.8	60.3	1.2	1.2	37.7	39.7	60.7
210095_s_at	IGFBP3	1.0	1.3	1.0	1.1	1.2	0.9	0.9	2.6	3.4	2.2	1.1	1.0	6.7	10.3	3.4
206924_at	IL11	1.0	1.0	7.4	10.0	10.9	1.0	1.0	2.8	2.7	3.7	0.9	1.0	2.9	2.4	7.6
210118_s_at	IL1A	1.1	1.7	7.2	10.4	12.3	1.7	0.8	4.2	4.1	5.0	0.9	1.1	1.6	3.9	2.0
202948_at	IL1R1	1.1	1.0	7.3	3.5	4.4	0.8	1.2	11.7	10.9	8.4	0.7	0.9	11.3	7.9	10.1
205403_at	IL1R2	1.1	0.9	1.2	1.1	2.0	1.1	0.8	1.9	1.6	1.5	1.0	0.9	4.4	2.0	3.0
212657_s_at	IL1RN	1.2	0.7	1.3	1.0	0.9	0.9	1.0	1.8	2.2	2.0	1.0	0.8	7.6	3.2	4.5
206569_at	IL24	0.9	0.8	4.0	1.3	1.7	1.2	1.2	84.5	57.0	52.8	1.1	0.9	122.3	35.5	66.4
203828_s_at	IL32	1.7	1.3	1.5	1.2	1.3	2.0	1.5	2.0	3.2	4.3	1.2	1.1	3.0	4.5	4.7
203233_at	IL4R	1.1	1.3	4.2	3.1	3.8	1.0	0.9	6.4	6.9	5.4	1.1	1.0	7.7	5.0	5.2
205207_at	IL6	1.1	1.4	2.8	2.9	4.0	0.8	0.9	4.7	3.1	4.6	1.1	0.9	4.4	2.0	2.9
211000_s_at	IL6ST	1.0	2.1	1.5	1.3	2.3	1.0	1.4	3.5	2.8	2.5	1.4	1.5	3.2	2.7	1.9
226218_at	IL7R	1.9	1.9	4.2	2.5	4.2	0.8	0.9	2.7	2.5	5.2	0.8	1.0	1.7	2.4	2.9
202859_x_at	IL8	2.1	2.8	58.3	40.1	46.3	1.0	1.1	9.9	10.7	16.1	1.0	1.2	4.0	5.6	3.8
238725_at	IRF1	0.9	1.2	5.6	4.4	5.8	1.1	0.9	7.9	6.7	4.0	1.0	1.1	7.2	4.9	2.3
203882_at	IRF9	2.3	1.5	2.5	1.4	1.5	1.4	1.2	4.6	5.0	8.2	1.1	0.9	4.6	4.2	5.3
205483_s_at	ISG15	1.4	1.2	2.3	1.7	3.2	1.2	1.2	3.9	2.9	7.4	1.1	1.0	3.4	4.0	6.4
33304_at	ISG20	1.0	1.1	1.8	1.6	2.4	0.9	0.8	6.7	5.8	7.3	1.0	1.0	8.9	9.9	10.0
227314_at	ITGA2	0.9	0.9	5.5	3.3	3.4	1.1	0.9	14.5	14.6	12.8	1.0	1.2	9.2	10.4	17.5
201656_at	ITGA6	0.9	1.0	1.3	1.3	1.2	1.0	1.0	3.2	3.1	2.7	0.9	1.3	3.0	2.7	2.1
204627_s_at	ITGB3	0.9	1.2	0.8	0.9	0.9	1.1	1.3	2.8	2.8	2.5	1.4	0.8	9.4	2.7	3.0
211339_s_at	ITK	1.2	0.9	1.2	1.1	0.9	1.1	1.0	4.3	2.5	2.2	1.1	1.3	13.9	2.8	3.4
1562031_at	JAK2	0.8	1.1	1.3	1.1	1.3	1.1	1.2	5.4	3.8	2.6	0.9	1.1	2.8	1.9	1.5
225142_at	JHDM1D	0.9	1.5	1.9	3.2	3.4	1.2	1.2	14.3	11.2	8.9	1.1	1.3	9.0	7.8	16.0
201466_s_at	JUN	0.9	1.0	9.2	12.1	11.2	1.2	0.8	7.4	6.1	3.8	1.1	1.3	7.1	5.5	1.7
213281_at	JUN	1.1	1.0	9.2	10.5	12.5	1.1	0.8	3.4	4.2	1.9	0.8	1.3	2.2	3.5	1.7
201473_at	JUNB	1.0	0.9	5.3	5.1	6.3	1.3	0.9	6.4	5.2	4.0	1.1	1.0	5.5	3.9	3.8
212188_at	KCTD12	0.7	1.0	3.4	3.6	4.0	0.8	1.3	7.1	8.6	5.4	1.1	1.2	3.4	4.7	2.3
228325_at	KIAA0146	1.0	1.6	17.4	19.3	18.0	1.1	1.1	10.3	13.2	9.1	1.1	1.0	7.4	5.4	7.3
212942_s_at	KIAA1199	1.0	1.2	0.8	1.0	1.0	1.1	1.0	2.4	1.7	1.7	1.1	1.0	18.3	15.7	26.1
211410_x_at	KIR2DL5A	1.1	0.9	1.2	1.0	0.9	1.1	1.0	3.1	3.0	2.8	0.9	0.9	2.8	2.8	3.1
219371_s_at	KLF2	0.8	1.0	2.4	4.8	5.2	0.9	1.1	3.6	3.6	1.5	1.0	1.0	4.1	4.7	0.9
1555832_s_at	KLF6	1.0	1.2	6.5	8.0	9.5	1.0	1.0	5.5	5.7	4.4	1.1	1.2	4.2	4.3	3.0
203543_s_at	KLF9	0.7	1.0	0.6	1.1	1.0	1.5	1.1	2.4	2.8	1.2	1.1	1.0	8.5	4.5	2.1
209351_at	KRT14	1.1	1.0	1.0	1.1	1.1	0.9	0.9	2.0	1.3	1.2	1.1	1.1	6.3	3.9	3.7
209800_at	KRT16	1.1	1.2	1.8	1.9	1.9	1.2	0.7	2.6	2.4	1.8	0.9	1.0	4.7	3.2	2.8
209125_at	KRT6A	1.1	1.2	1.0	0.9	1.1	1.0	0.9	1.5	1.6	1.6	0.9	0.9	5.0	1.8	1.7
231849_at	KRT80	0.8	0.9	2.6	1.7	1.5	0.9	1.0	3.8	3.0	2.4	1.0	1.1	1.4	1.5	1.4
235252_at	KSR1	1.0	0.9	2.2	1.4	1.3	1.1	0.9	5.3	4.3	3.3	1.0	1.0	4.4	2.9	2.1
1552486_s_at	LACTB	1.8	1.4	2.0	1.8	1.9	1.3	1.0	3.0	3.4	2.8	0.8	1.0	2.4	2.6	1.8
205569_at	LAMP3	1.1	1.3	0.9	1.0	0.8	1.3	1.2	3.5	1.8	2.5	0.8	1.3	4.4	4.9	4.2
214461_at	LBP	1.1	1.1	1.3	1.0	1.2	0.9	1.0	10.6	10.7	6.2	1.3	0.9	57.8	15.4	15.1
210732_s_at	LGALS8	1.2	1.3	1.1	1.4	1.3	1.3	0.9	4.0	4.0	2.8	1.2	1.5	11.2	9.1	5.4
236898_at	LOC100288781	1.5	1.4	4.1	3.2	4.3	1.1	0.9	2.6	1.9	1.8	1.1	1.1	3.2	2.2	1.8
1568768_s_at	LOC100302650	1.4	1.6	8.4	6.6	6.9	0.9	0.7	4.0	4.2	3.9	0.8	0.9	1.3	2.3	2.6
227452_at	LOC100499467	0.8	1.3	2.9	3.3	3.0	1.1	0.9	4.4	4.3	3.8	1.0	1.1	4.2	3.4	4.5
230710_at	LOC100506211	1.0	1.2	0.5	0.7	0.9	1.0	1.2	1.5	2.2	1.0	1.1	1.0	4.2	5.2	2.3
227868_at	LOC154761	1.3	1.2	2.2	1.4	1.4	1.0	0.9	4.3	5.1	3.0	1.0	0.9	7.4	6.6	2.4
204298_s_at	LOX	0.9	1.2	0.9	1.0	1.0	1.0	1.0	2.4	3.3	1.6	1.0	1.1	5.5	12.1	2.9
202998_s_at	LOXL2	1.1	1.0	1.1	1.0	0.9	1.2	0.9	1.9	1.3	1.1	1.1	1.1	5.2	4.3	3.5
230252_at	LPAR5	0.9	1.0	0.9	0.9	1.0	0.9	0.8	2.2	2.6	1.4	1.0	1.2	13.5	8.4	3.3
243874_at	LPP	1.2	1.3	3.7	2.9	2.1	0.8	0.8	1.3	1.2	0.9	0.9	1.0	1.3	1.1	0.8
228648_at	LRG1	0.8	1.0	1.3	1.3	1.3	0.9	1.2	7.7	5.1	3.6	1.3	1.0	21.5	7.6	4.5
231861_at	LRP10	0.8	1.2	1.1	0.9	1.4	1.0	1.4	3.6	2.8	1.4	0.9	1.2	5.2	4.4	2.4
227252_at	LRP10	0.9	1.0	1.0	1.1	1.1	1.0	1.3	3.0	2.7	1.7	1.1	1.0	4.7	4.1	2.5
206584_at	LY96	1.0	1.3	1.0	1.3	1.0	0.9	0.8	2.3	1.4	1.1	1.0	1.0	13.8	2.8	3.2
1554179_s_at	LYNX1	0.9	0.8	1.0	0.9	0.8	1.1	1.0	1.2	1.0	1.2	0.9	0.9	5.1	2.8	4.2
218559_s_at	MAFB	0.8	1.0	5.1	5.7	6.6	1.1	0.7	4.2	3.4	2.4	0.9	1.8	5.5	4.9	3.3
36711_at	MAFF	1.2	1.4	6.8	5.9	6.0	1.1	0.9	5.3	5.8	5.6	1.0	1.0	2.3	4.2	3.9
226206_at	MAFK	1.0	0.8	3.5	2.5	2.3	1.0	0.8	2.9	2.2	2.0	0.9	0.8	2.2	1.9	2.5
218918_at	MAN1C1	0.9	1.2	1.2	1.1	1.0	0.9	0.8	3.7	3.6	1.8	1.0	1.3	17.5	13.5	4.2
205027_s_at	MAP3K8	0.8	1.2	4.6	5.6	5.5	1.0	1.1	5.8	7.8	4.7	0.9	1.0	8.6	6.5	4.6
225613_at	MAST4	1.0	1.4	1.0	1.1	1.6	1.0	1.2	2.1	1.9	1.6	1.1	1.2	4.4	3.2	3.6
203640_at	MBNL2	1.1	1.0	1.6	1.1	1.1	1.0	1.1	3.1	3.2	2.7	1.0	1.1	3.5	3.0	2.7

				4 h					10 h					20 h		
**Probeset**	**GeneID**	***invA***	***invA***	**wt**	**wt**	***Δasd***	***invA***	***invA***	**wt**	**wt**	***Δasd***	***invA***	***invA***	**wt**	**wt**	***Δasd***
214056_at	MCL1	1.1	1.3	4.0	3.8	3.7	1.1	0.9	3.4	3.0	3.0	1.0	1.2	3.1	3.7	3.7
235740_at	MCTP1	0.8	1.1	1.1	0.8	0.8	1.2	1.3	5.1	3.4	3.2	1.0	1.3	4.5	5.6	5.7
220603_s_at	MCTP2	1.0	0.9	1.6	1.1	1.1	1.0	1.0	3.3	3.2	2.7	1.0	1.0	2.7	2.0	1.8
212473_s_at	MICAL2	1.0	1.2	2.5	2.2	2.5	1.0	0.9	3.1	2.9	2.5	0.9	1.2	1.5	2.1	2.1
224917_at	MIR21	1.2	1.2	2.4	2.2	2.2	1.1	1.1	1.8	1.7	2.0	1.1	1.3	2.7	3.6	4.2
203780_at	MPZL2	0.8	0.8	2.0	1.5	1.5	1.0	1.5	4.3	5.6	5.2	0.9	1.1	3.7	4.4	5.6
216336_x_at	MT1E/MT1H/MT1M	1.4	1.2	2.1	1.9	2.3	1.0	1.0	3.4	2.3	2.0	1.0	1.0	2.9	3.4	3.9
217165_x_at	MT1F	1.3	1.4	1.6	2.0	1.9	1.1	1.0	3.3	3.5	3.0	1.1	1.3	3.8	4.9	4.9
206461_x_at	MT1H	1.3	1.2	1.9	1.9	2.1	1.1	1.1	3.2	3.3	3.0	1.0	1.1	3.8	4.4	4.5
211456_x_at	MT1P2	1.3	1.2	1.8	1.8	2.1	1.1	0.9	3.0	2.9	2.7	1.1	1.0	3.8	4.1	3.8
204326_x_at	MT1X	1.2	1.1	2.0	1.9	2.2	1.0	0.9	4.2	4.7	3.6	1.1	1.0	5.7	7.0	5.9
212185_x_at	MT2A	1.3	1.2	2.0	1.9	2.0	1.1	1.0	3.1	2.9	2.7	1.1	1.1	3.8	4.4	3.8
207847_s_at	MUC1	1.7	1.1	1.2	1.1	1.2	1.6	1.2	2.5	2.4	2.4	1.3	1.0	7.4	6.3	5.0
218687_s_at	MUC13	1.0	0.8	2.0	1.3	1.4	1.0	0.9	6.4	4.2	4.0	1.1	0.9	5.0	3.3	3.7
202180_s_at	MVP	1.1	1.1	1.2	1.0	1.0	1.1	1.2	2.4	2.6	2.3	1.2	1.1	5.1	3.4	3.4
202086_at	MX1	1.0	0.7	1.2	1.1	1.3	1.1	0.8	12.5	8.1	10.3	1.4	0.7	38.6	10.9	11.5
228846_at	MXD1	0.9	1.0	1.7	2.0	2.3	0.9	1.3	2.6	3.0	2.0	0.8	1.3	2.7	3.4	3.3
206394_at	MYBPC2	1.1	0.8	1.1	0.9	0.8	1.0	1.0	1.0	1.1	1.2	0.9	1.0	16.7	4.4	1.3
243296_at	NAMPT	0.7	1.2	5.1	4.9	4.3	0.9	0.9	7.3	5.4	3.2	1.5	1.4	10.7	6.2	6.2
210048_at	NAPG	1.2	1.2	1.3	1.4	1.3	1.1	0.9	2.0	1.8	1.6	1.0	1.0	3.8	2.6	2.3
219862_s_at	NARF	0.9	1.1	1.0	1.0	1.0	1.0	1.0	2.8	2.5	1.8	1.1	1.0	4.7	3.7	2.7
225344_at	NCOA7	1.1	1.2	3.0	2.0	2.3	0.8	1.0	4.0	3.6	3.1	1.1	1.1	3.8	3.3	2.5
1562034_at	NCRNA00163	1.2	1.0	4.4	2.5	3.1	0.9	0.8	1.1	0.8	1.0	1.0	1.2	1.0	1.1	1.1
227062_at	NEAT1	0.7	1.1	3.1	2.4	3.3	0.7	1.3	4.3	3.9	2.7	1.2	1.1	5.4	5.0	2.6
209930_s_at	NFE2	1.4	0.9	1.3	1.0	1.4	1.0	1.1	6.2	7.4	5.3	1.0	1.0	21.5	12.3	8.5
203574_at	NFIL3	1.0	0.9	4.1	4.0	4.0	1.0	0.8	3.3	2.7	1.8	0.9	0.9	3.0	2.5	1.5
223218_s_at	NFKBIZ	1.1	1.4	7.9	9.4	10.3	1.1	1.2	5.2	5.4	4.6	1.0	1.3	4.1	3.5	3.6
229491_at	NHEDC2	1.1	1.0	1.9	1.5	1.3	1.2	1.2	4.2	4.0	3.9	1.0	1.2	4.6	3.4	3.9
205220_at	NIACR2	1.2	1.7	2.8	2.8	4.2	0.8	1.0	19.6	17.7	11.7	1.1	1.4	38.3	38.8	20.2
202238_s_at	NNMT	1.3	1.1	3.8	2.4	3.0	1.0	0.9	11.7	11.1	9.3	1.0	1.1	12.5	11.0	10.0
213462_at	NPAS2	1.0	1.2	1.7	1.3	1.3	1.2	0.7	2.6	1.8	1.8	1.0	1.2	3.5	3.1	2.6
202679_at	NPC1	0.8	0.9	1.9	1.5	1.6	1.0	1.0	3.1	2.9	2.3	1.0	0.9	2.6	2.1	2.8
202340_x_at	NR4A1	1.4	0.8	3.9	3.7	3.2	1.1	0.9	0.7	0.7	0.7	1.0	0.8	0.6	0.8	0.8
207978_s_at	NR4A3	0.8	0.6	2.5	3.8	2.3	1.0	0.9	0.7	0.4	0.5	1.1	0.8	0.6	0.5	0.4
212298_at	NRP1	0.8	1.6	1.4	2.3	2.3	1.3	1.2	4.9	5.6	4.9	1.1	1.3	6.7	4.5	3.9
203939_at	NT5E	0.8	1.0	1.8	1.8	1.7	0.9	1.0	3.6	2.5	3.2	1.0	1.1	4.1	4.8	8.6
206291_at	NTS	1.2	1.2	1.4	0.9	0.9	0.9	1.0	4.0	1.5	1.6	1.2	0.9	15.8	7.7	18.1
205660_at	OASL	1.0	0.9	1.1	0.8	1.8	1.1	1.0	8.8	5.6	16.6	1.0	1.2	9.5	7.8	13.9
233085_s_at	OBFC2A	1.0	1.0	5.6	4.7	4.6	1.0	1.1	6.2	6.4	6.3	1.0	1.2	3.2	2.6	2.8
210004_at	OLR1	5.8	5.6	3.8	5.7	9.6	1.6	1.2	3.4	4.2	7.2	1.1	1.2	1.4	1.4	2.2
205729_at	OSMR	2.1	1.2	2.8	2.4	2.6	2.2	0.8	10.2	7.4	6.4	1.2	1.0	11.3	7.1	8.0
226621_at	OSMR	1.0	1.0	3.2	2.2	2.2	1.0	1.1	6.9	6.0	5.4	1.1	1.1	7.6	5.8	5.6
230830_at	OSTbeta	1.0	1.1	3.1	2.5	2.8	1.3	1.2	4.3	3.6	2.6	1.1	0.9	2.2	1.3	0.7
226140_s_at	OTUD1	1.0	1.1	3.4	2.8	3.9	1.1	1.2	4.3	3.9	3.6	0.9	1.1	4.4	3.5	4.5
202733_at	P4HA2	0.8	0.9	1.0	1.0	0.9	1.0	1.1	1.6	1.6	1.3	1.0	1.0	3.5	3.7	2.0
226694_at	PALM2-AKAP2	1.5	2.3	5.7	4.0	5.0	0.8	0.8	12.4	11.7	9.5	0.8	0.9	8.8	7.8	5.4
202760_s_at	PALM2-AKAP2	1.0	2.0	2.8	3.0	4.1	1.1	0.7	5.6	5.1	3.4	0.9	0.9	4.1	2.8	2.2
204715_at	PANX1	1.2	1.4	2.2	1.9	2.4	0.9	1.1	3.1	3.5	3.1	1.1	1.0	2.9	2.3	2.0
228128_x_at	PAPPA	0.9	1.1	1.4	1.0	1.2	0.8	1.5	7.1	10.1	8.3	0.7	1.0	2.7	2.4	2.9
203060_s_at	PAPSS2	0.9	1.0	1.2	0.9	0.9	1.1	1.1	4.6	3.9	2.9	1.0	1.0	3.9	3.8	3.0
224701_at	PARP14	1.8	1.7	1.1	1.2	1.7	1.2	1.5	3.4	3.3	5.7	1.3	0.9	6.6	3.9	3.7
223220_s_at	PARP9	1.2	1.4	1.4	1.2	1.3	1.0	1.2	3.9	4.3	5.4	1.0	1.2	3.9	3.2	3.3
228640_at	PCDH7	0.9	1.0	1.2	0.8	1.0	0.9	1.3	8.5	13.0	8.6	0.8	1.2	0.7	1.1	1.3
218273_s_at	PDP1	0.7	1.6	3.1	3.3	3.1	0.9	1.1	4.7	4.2	3.8	1.1	1.5	1.5	1.8	1.7
209493_at	PDZD2	1.5	1.8	1.8	1.2	1.2	1.2	1.1	9.1	11.2	10.4	1.4	1.2	5.8	5.8	9.2
219630_at	PDZK1IP1	1.4	1.3	1.3	1.1	1.2	1.2	1.3	3.0	2.5	2.1	1.1	1.1	6.4	4.4	3.8
218319_at	PELI1	1.0	1.2	2.3	2.6	2.9	1.2	1.2	3.3	4.1	3.0	1.0	1.2	2.8	2.9	2.4
214660_at	PELO	1.1	1.2	1.0	1.2	1.1	1.3	1.7	3.1	4.3	3.8	0.9	1.3	5.1	5.0	4.9
202464_s_at	PFKFB3	0.8	0.8	3.4	2.6	2.9	0.9	1.0	5.0	5.5	3.4	1.0	1.1	3.7	4.3	2.6
228499_at	PFKFB4	1.1	1.1	1.6	1.4	1.8	1.0	0.9	1.3	1.9	1.1	1.0	1.1	2.4	4.2	2.0
217996_at	PHLDA1	1.1	1.2	4.3	3.8	3.8	1.3	0.8	8.7	6.2	6.6	1.0	1.2	4.4	6.1	5.6
215236_s_at	PICALM	2.2	1.0	2.2	1.7	1.8	1.5	1.0	2.6	3.6	3.0	1.0	1.1	1.8	2.0	2.5
219155_at	PITPNC1	0.9	1.0	1.3	1.1	1.2	0.9	0.9	9.5	7.8	5.8	1.0	1.0	9.3	7.8	8.0
223551_at	PKIB	1.0	1.4	1.2	1.4	1.4	1.4	1.1	2.6	3.6	2.5	0.7	0.9	2.2	1.7	2.4
201860_s_at	PLAT	1.1	1.0	1.2	1.0	1.2	1.1	1.1	3.0	1.6	1.5	1.0	0.9	10.8	5.4	6.3
211924_s_at	PLAUR	1.3	1.1	4.6	3.7	5.8	1.1	0.8	6.0	5.2	4.9	0.8	0.9	6.0	6.8	6.6
202122_s_at	PLIN3	1.1	0.9	1.9	1.5	1.6	1.1	1.0	2.8	2.8	2.8	1.0	1.0	3.6	2.7	3.3
202619_s_at	PLOD2	0.9	1.0	1.1	1.0	1.1	1.2	1.1	3.4	2.5	1.9	1.0	1.1	6.6	5.5	3.5
202446_s_at	PLSCR1	1.3	1.1	2.2	1.4	1.6	1.2	1.0	6.9	5.7	5.3	1.1	1.1	6.6	4.9	4.9
204285_s_at	PMAIP1	1.0	1.1	3.6	3.6	4.9	1.0	1.0	2.3	2.0	2.0	1.1	1.0	1.7	1.5	1.4
212230_at	PPAP2B	0.9	0.9	4.3	2.1	2.2	1.0	0.8	6.1	5.2	4.6	0.9	1.1	5.4	3.6	4.2
214146_s_at	PPBP	1.0	1.3	1.0	1.0	1.1	1.0	1.2	2.5	1.4	1.1	1.1	0.9	5.2	2.0	3.6
214978_s_at	PPFIA4	0.9	1.0	1.0	1.0	1.0	0.9	0.8	1.0	1.9	0.9	0.8	0.8	5.1	17.4	2.7
37028_at	PPP1R15A	1.2	1.0	2.8	3.6	3.6	1.4	0.9	1.5	1.4	1.4	1.1	0.9	1.1	1.2	1.2
222662_at	PPP1R3B	0.9	1.0	6.1	3.6	3.0	0.9	0.9	7.0	8.2	5.4	1.1	1.2	8.3	6.9	4.3
228964_at	PRDM1	1.1	1.1	2.1	2.6	2.4	1.2	1.4	3.6	3.8	3.2	1.0	1.1	3.3	3.1	2.8
203680_at	PRKAR2B	1.0	1.3	0.9	1.5	1.2	1.1	1.3	3.3	2.9	2.3	0.7	1.1	1.6	1.9	2.0
207808_s_at	PROS1	0.9	1.0	1.0	0.9	1.1	0.9	1.1	3.1	3.3	2.4	1.0	1.1	7.4	4.4	3.6
202659_at	PSMB10	1.6	1.6	1.2	1.1	1.3	1.6	1.8	2.0	1.9	2.1	1.0	1.1	3.5	2.8	2.8
204279_at	PSMB9	1.8	2.3	0.9	0.9	0.9	1.5	2.0	1.6	2.1	2.8	1.2	1.0	4.7	3.5	3.5
1554997_a_at	PTGS2	1.1	1.8	7.8	13.4	21.1	1.2	1.3	1.3	1.5	2.1	1.3	1.3	1.1	2.1	1.9
221840_at	PTPRE	1.4	1.3	4.8	2.5	2.4	1.0	0.8	8.5	5.8	4.6	1.0	1.1	6.7	3.3	6.6
208121_s_at	PTPRO	1.2	0.7	0.9	0.9	0.9	1.0	1.2	4.5	2.7	2.8	1.2	1.0	5.2	1.9	2.6
206157_at	PTX3	1.9	2.3	5.1	3.1	7.3	1.2	0.9	2.9	3.5	4.4	1.0	1.3	1.2	2.9	1.4
209514_s_at	RAB27A	1.0	1.0	1.5	1.3	1.2	1.0	1.0	3.5	3.0	2.6	1.1	1.1	3.1	2.3	2.2
217763_s_at	RAB31	1.1	1.1	1.4	1.1	1.2	1.1	0.8	4.8	3.1	2.5	0.9	0.9	7.1	4.6	4.8
214435_x_at	RALA	1.0	1.1	1.3	1.2	1.4	1.2	1.4	3.4	4.0	3.2	1.0	1.1	5.6	4.3	3.7
209050_s_at	RALGDS	0.9	1.0	1.2	1.3	1.5	0.9	1.1	2.9	3.0	1.7	1.1	1.1	5.1	4.3	1.9
205651_x_at	RAPGEF4	1.1	0.8	1.1	0.7	0.8	1.0	1.7	9.3	7.4	3.2	0.9	1.3	23.8	12.4	6.6
1557432_at	RASAL2	1.2	1.1	5.5	4.0	3.1	1.1	0.8	1.9	2.1	1.4	1.1	1.0	1.6	1.5	1.1
223467_at	RASD1	0.9	0.8	4.5	3.2	4.2	0.9	0.8	6.3	5.5	6.2	1.1	1.0	2.7	3.4	6.3
230563_at	RASGEF1A	1.1	1.2	2.4	2.5	2.7	1.0	0.8	9.1	6.7	3.6	1.0	0.8	14.7	5.3	2.0

				4 h					10 h					20 h		
**Probeset**	**GeneID**	***invA***	***invA***	**wt**	**wt**	***Δasd***	***invA***	***invA***	**wt**	**wt**	***Δasd***	***invA***	***invA***	**wt**	**wt**	***Δasd***
204364_s_at	REEP1	0.7	0.9	1.1	0.9	0.9	1.0	0.9	6.3	5.5	3.5	1.0	1.0	7.0	4.5	3.1
209324_s_at	RGS16	0.9	0.9	3.6	2.8	4.6	0.8	1.1	14.3	14.2	10.4	1.1	1.0	26.7	19.7	9.9
220334_at	RGS17	0.7	1.1	1.9	1.7	1.3	1.0	1.0	5.7	5.6	3.2	1.1	1.0	2.4	1.9	1.6
202388_at	RGS2	0.9	0.7	3.2	2.4	2.4	0.9	0.8	3.7	3.2	3.2	1.0	0.9	3.2	2.6	4.1
244675_at	RGS8	1.0	1.0	1.1	1.1	0.9	0.9	1.0	1.9	1.5	1.2	1.1	1.1	9.3	4.6	2.3
212122_at	RHOQ	1.0	1.2	2.1	1.8	1.8	0.9	1.2	6.7	7.0	5.5	1.1	1.3	13.0	8.1	11.3
210056_at	RND1	0.9	1.1	1.7	1.8	2.0	0.8	1.3	7.1	6.4	3.7	1.2	1.1	22.0	9.7	2.2
235199_at	RNF125	0.8	0.9	1.1	1.3	1.0	1.0	0.8	4.3	4.1	2.8	1.0	1.3	4.7	3.7	3.3
204669_s_at	RNF24	1.2	1.1	2.4	1.9	1.6	1.1	1.0	5.5	6.5	4.6	1.0	0.8	9.0	5.0	4.3
224618_at	ROD1	0.9	1.3	1.5	1.6	1.9	0.9	1.6	2.6	3.9	2.8	1.0	1.2	2.3	2.3	2.4
210426_x_at	RORA	0.8	1.4	1.4	1.8	2.5	1.1	1.1	2.1	1.5	1.0	1.1	1.0	4.7	6.6	2.7
210251_s_at	RUFY3	0.9	1.2	1.7	1.7	1.8	1.1	1.2	3.4	3.1	1.9	1.1	1.2	3.1	2.3	1.6
238909_at	S100A10	0.8	0.9	4.0	3.0	3.1	1.0	1.1	3.1	3.3	2.2	1.2	1.2	4.7	4.3	2.3
202917_s_at	S100A8	1.0	0.6	1.3	0.7	0.8	1.1	1.2	1.1	1.4	1.1	1.1	0.9	5.4	2.5	3.3
204642_at	S1PR1	1.0	1.0	1.8	1.6	1.5	1.0	0.9	3.0	2.6	1.9	1.0	0.9	10.0	2.8	2.1
208607_s_at	SAA1/SAA2	2.5	2.5	5.6	4.5	5.7	2.0	1.8	20.2	20.4	16.9	2.0	2.2	32.3	26.9	23.3
212845_at	SAMD4A	1.4	2.4	2.0	2.6	2.8	1.1	1.2	10.3	9.5	6.7	1.3	1.3	10.3	7.4	3.8
213988_s_at	SAT1	1.7	1.9	4.1	4.1	4.6	1.7	1.7	6.7	5.5	5.0	1.0	1.3	5.2	4.5	4.8
202071_at	SDC4	1.1	1.2	3.0	3.4	4.5	1.0	1.2	2.1	2.5	2.4	1.0	1.0	1.9	1.8	1.7
238017_at	SDR16C5	1.9	1.7	4.5	2.0	2.8	1.0	1.6	34.1	41.7	32.6	1.5	1.5	132.2	66.6	88.0
213716_s_at	SECTM1	1.2	1.4	1.6	2.3	2.5	1.4	1.3	15.9	15.5	12.4	1.1	0.7	21.5	9.2	8.9
234725_s_at	SEMA4B	1.0	1.1	2.6	2.1	2.0	1.2	1.0	5.0	4.8	3.3	1.0	1.0	4.6	3.9	5.7
1559025_at	SEPT9	0.9	1.3	3.4	2.8	2.0	1.0	1.1	2.6	2.1	1.9	1.1	1.0	2.7	2.1	1.6
217977_at	SEPX1	1.0	0.9	1.0	0.7	0.8	1.0	1.0	2.1	2.2	1.8	1.0	1.1	3.7	2.8	2.0
224762_at	SERINC2	1.1	1.0	1.0	0.9	0.9	1.1	1.0	1.8	1.6	1.3	1.0	0.8	4.8	2.5	2.3
212812_at	SERINC5	0.9	0.9	1.3	1.4	1.3	1.0	1.1	2.9	3.7	3.0	1.0	1.1	5.0	3.9	3.3
202833_s_at	SERPINA1	1.0	1.0	1.1	1.0	1.2	1.0	1.1	1.2	1.8	1.5	1.0	0.9	26.6	5.4	13.5
212268_at	SERPINB1	1.2	1.0	3.0	1.9	2.2	1.1	1.1	11.0	10.0	7.4	1.2	1.1	14.7	12.0	6.2
228726_at	SERPINB1	0.9	1.0	2.8	2.1	2.2	1.0	1.1	9.6	10.8	7.5	1.0	1.1	10.1	9.4	4.6
209719_x_at	SERPINB3	1.3	1.0	82.4	30.8	33.3	1.5	1.1	725.2	392.3	392.1	5.5	2.0	873.9	753.8	1011.6
211906_s_at	SERPINB4	0.8	1.1	40.8	20.4	20.9	1.3	0.8	481.2	406.2	408.9	2.7	1.1	934.4	709.0	1090.8
206034_at	SERPINB8	1.3	1.2	2.2	1.6	1.9	0.9	1.2	4.1	4.0	4.1	1.0	1.1	5.0	4.3	4.2
212190_at	SERPINE2	1.3	1.5	3.7	2.3	3.2	1.1	1.1	3.4	3.1	3.3	0.8	0.9	2.3	3.6	3.3
223394_at	SERTAD1	1.0	1.0	3.0	2.8	3.6	1.0	1.0	3.3	2.7	2.2	1.0	1.0	3.0	3.1	1.8
227038_at	SGMS2	0.9	0.8	1.6	1.6	1.6	1.1	1.3	2.9	3.4	3.1	0.8	1.2	1.6	1.7	1.7
1553177_at	SH2D1B	1.6	1.8	1.7	2.1	1.7	1.2	0.9	3.9	3.5	3.3	1.0	1.4	2.6	2.5	2.1
217257_at	SH3BP2	0.9	1.5	1.2	1.7	1.3	1.4	1.1	2.9	3.8	1.8	0.9	1.0	3.5	3.6	1.3
219256_s_at	SH3TC1	1.0	1.1	2.7	2.4	2.9	1.0	0.8	3.7	3.8	3.0	1.0	0.9	3.2	1.9	2.0
1557458_s_at	SHB	1.1	1.1	3.4	2.4	3.1	1.1	1.0	3.8	3.7	3.6	0.9	1.1	1.9	2.5	2.4
233587_s_at	SIPA1L2	0.8	1.9	2.6	2.2	2.9	1.0	1.3	4.9	4.7	3.6	0.9	1.1	4.1	2.6	2.9
225619_at	SLAIN1	1.1	0.9	2.0	1.1	1.1	1.0	1.4	5.9	7.6	4.8	1.1	1.1	10.6	5.0	1.7
213664_at	SLC1A1	0.9	1.1	1.1	0.9	1.0	1.1	1.0	2.3	2.2	2.1	1.0	1.1	2.4	3.9	3.2
202800_at	SLC1A3	1.0	1.0	1.7	1.3	1.4	1.0	1.0	6.7	5.5	4.7	1.0	1.1	7.3	6.2	1.8
205896_at	SLC22A4	1.0	1.2	1.4	1.0	1.0	1.0	1.0	3.5	2.8	2.1	1.0	0.9	6.0	3.6	3.8
222528_s_at	SLC25A37	1.1	1.2	2.0	1.4	2.1	1.1	1.1	3.1	3.2	2.6	1.0	1.1	2.2	2.4	1.9
232277_at	SLC28A3	1.3	1.3	1.2	1.1	1.3	1.4	1.0	7.7	1.7	2.8	1.1	1.1	2.7	1.5	3.4
209267_s_at	SLC39A8	1.2	1.1	1.1	1.1	1.1	1.2	1.2	3.1	3.1	3.2	0.9	1.1	1.9	2.1	2.7
206376_at	SLC6A15	1.1	1.0	1.1	1.0	1.1	1.1	0.8	5.8	4.5	3.9	1.0	1.1	6.5	6.3	7.5
205921_s_at	SLC6A6	0.9	2.8	1.0	2.2	1.9	1.4	1.1	5.4	5.6	6.3	1.0	1.0	6.3	4.0	4.6
219911_s_at	SLCO4A1	0.8	1.0	1.5	1.5	1.6	1.0	0.9	4.1	3.4	2.8	0.9	1.0	4.1	3.5	4.1
205315_s_at	SNTB2	0.7	1.1	1.2	1.1	1.2	0.7	1.1	3.9	3.6	2.2	1.1	1.2	6.5	4.6	2.3
210001_s_at	SOCS1	0.9	1.2	1.5	1.6	2.3	1.1	1.0	14.8	9.7	6.4	0.9	1.1	26.6	8.2	6.7
203372_s_at	SOCS2	1.3	0.9	7.8	5.0	4.6	1.2	0.9	22.8	29.9	16.0	1.3	1.0	33.6	18.1	7.1
227697_at	SOCS3	0.8	0.5	5.5	5.6	6.4	1.1	1.0	6.6	7.2	5.7	0.9	0.9	4.3	3.7	3.3
215223_s_at	SOD2	2.4	2.7	4.9	4.2	5.6	2.2	3.0	9.0	8.5	9.5	1.1	1.3	13.3	12.4	13.6
222513_s_at	SORBS1	1.2	1.0	1.1	1.1	0.9	1.0	1.2	2.6	2.1	1.9	0.9	0.9	4.2	2.6	1.4
212780_at	SOS1	1.0	1.3	1.1	1.3	1.1	0.9	1.0	1.8	1.9	1.4	1.0	1.1	4.2	3.7	1.4
202935_s_at	SOX9	0.9	1.2	3.5	4.0	5.0	1.2	0.9	2.6	1.7	1.3	0.9	1.0	4.0	2.8	1.0
209762_x_at	SP110	1.1	1.1	1.0	0.9	0.9	1.0	0.8	4.3	2.6	2.4	1.1	0.9	2.3	1.6	1.8
219257_s_at	SPHK1	1.9	1.5	4.2	3.3	3.7	1.3	0.9	3.0	3.1	3.0	0.9	0.8	1.6	1.2	1.1
206239_s_at	SPINK1	1.2	1.2	3.5	2.6	2.1	1.0	1.4	20.3	21.1	13.9	1.2	0.9	76.2	26.3	27.3
1553973_a_at	SPINK6	1.1	1.0	1.3	1.3	1.6	1.0	0.9	2.3	2.4	2.4	1.0	1.0	3.2	2.8	5.9
209875_s_at	SPP1	1.1	1.0	1.1	0.9	1.2	0.9	1.0	0.9	1.0	0.9	1.0	1.1	37.3	4.2	2.3
217995_at	SQRDL	1.1	1.3	1.3	1.1	1.4	1.0	1.3	4.2	4.2	4.4	1.1	1.2	5.9	4.8	5.7
204955_at	SRPX	1.2	1.9	1.2	1.2	1.7	0.7	1.0	1.4	1.4	1.2	0.8	1.0	3.6	4.3	2.2
202440_s_at	ST5	1.0	1.1	1.1	1.0	1.0	1.1	1.0	2.9	2.8	2.0	1.1	0.9	4.7	2.3	2.0
227607_at	STAMBPL1	1.3	1.1	2.9	1.8	1.9	1.0	0.8	3.3	2.8	2.6	1.0	1.0	2.3	3.2	1.9
243213_at	STAT3	1.2	1.1	5.0	4.2	2.4	1.0	1.0	2.0	1.3	1.5	0.9	1.1	1.3	1.3	1.4
208992_s_at	STAT3	1.5	1.1	1.9	1.6	1.5	1.0	1.1	3.0	2.9	2.6	1.1	1.0	4.7	3.0	2.9
205542_at	STEAP1	1.1	1.3	1.8	1.7	1.5	1.0	1.0	3.6	3.5	3.1	0.9	1.0	4.2	3.5	3.4
1554830_a_at	STEAP3	1.2	1.1	1.2	0.8	1.1	0.9	1.2	2.3	2.4	2.0	0.7	0.9	3.9	2.4	1.3
220187_at	STEAP4	1.2	1.2	2.7	2.7	2.8	1.1	0.9	5.6	5.2	4.3	0.9	1.1	8.5	5.9	3.6
1556185_a_at	STEAP4	1.0	1.2	1.8	1.3	1.4	1.1	0.9	5.9	4.2	2.8	1.0	1.2	4.1	3.1	2.3
201060_x_at	STOM	1.0	0.9	1.9	1.7	1.5	1.0	1.1	5.0	5.8	4.7	1.0	1.0	5.6	4.4	2.9
213413_at	STON1	0.9	1.4	0.5	1.1	1.0	1.1	1.2	1.4	1.8	1.7	1.1	1.3	19.1	10.6	16.6
203767_s_at	STS	1.0	1.0	1.1	1.1	1.1	1.2	1.2	3.2	3.3	3.0	1.0	1.1	3.6	3.2	2.7
220030_at	STYK1	0.9	1.2	2.4	1.1	1.3	1.2	1.0	10.1	6.8	4.4	1.2	1.2	14.2	7.1	2.8
205513_at	TCN1	1.1	0.8	1.1	1.1	1.0	0.8	0.9	5.4	4.3	4.7	0.9	1.1	39.9	24.5	25.0
205016_at	TGFA	1.2	1.4	1.4	1.4	1.0	1.0	1.0	2.8	3.5	3.1	1.0	1.3	1.7	2.3	2.4
201042_at	TGM2	0.9	0.8	2.5	1.6	1.6	1.1	0.7	9.7	7.2	5.2	0.8	0.8	6.6	5.7	6.5
230380_at	THAP2	1.2	1.5	4.1	4.7	5.3	1.1	1.1	3.1	3.8	4.9	0.9	1.1	2.0	2.0	5.4
203887_s_at	THBD	1.0	1.1	3.1	3.4	2.0	1.0	0.9	4.4	4.6	3.1	0.8	0.9	2.6	1.6	2.0
235086_at	THBS1	1.0	2.2	3.3	5.4	5.7	1.3	1.2	3.2	2.4	2.0	0.9	1.1	1.4	1.4	1.3
1557275_a_at	TLCD2	1.0	0.8	1.7	1.0	1.3	0.9	1.2	2.7	2.3	1.4	1.1	1.0	4.7	3.0	2.1
241359_at	TLCD2	0.8	1.0	1.1	1.1	1.2	0.8	1.2	2.7	2.5	1.6	1.0	0.9	5.6	3.0	1.9
240304_s_at	TMC5	0.6	1.5	2.7	1.8	2.6	0.8	1.4	5.0	5.4	3.6	1.0	1.4	4.4	4.3	3.8
224916_at	TMEM173	1.2	1.0	1.7	1.1	1.2	1.2	1.2	4.7	5.4	4.2	1.1	0.9	12.3	6.9	5.0
218113_at	TMEM2	1.0	1.1	3.2	2.7	2.4	1.1	0.9	8.4	6.9	5.5	1.1	1.1	9.6	7.6	5.2
223784_at	TMEM27	0.8	0.9	0.8	0.8	1.1	1.1	1.2	2.6	1.7	3.0	1.0	1.1	5.0	3.7	5.3
219410_at	TMEM45A	1.0	0.9	0.8	0.9	0.9	0.9	1.0	0.7	1.0	0.7	1.0	1.1	2.9	4.5	2.4
230323_s_at	TMEM45B	0.8	1.3	0.7	1.0	1.0	1.0	0.9	2.2	2.2	1.6	1.0	1.3	3.0	3.4	3.7
226931_at	TMTC1	0.6	0.8	0.9	0.7	0.5	1.1	0.8	3.4	2.7	2.1	0.8	1.0	1.7	2.4	2.4

				4 h					10 h					20 h		
**Probeset**	**GeneID**	***invA***	***invA***	**wt**	**wt**	***Δasd***	***invA***	***invA***	**wt**	**wt**	***Δasd***	***invA***	***invA***	**wt**	**wt**	***Δasd***
225302_at	TMX3	0.9	0.9	2.3	1.6	1.6	1.0	1.1	3.4	3.2	2.8	1.0	1.2	1.7	1.5	1.7
201645_at	TNC	1.4	1.1	3.1	1.1	2.1	1.7	1.4	22.5	11.8	9.5	1.4	1.3	97.1	36.6	33.2
202643_s_at	TNFAIP3	1.3	1.4	6.4	5.1	7.4	1.3	1.2	2.3	2.0	2.7	0.9	1.2	1.0	1.2	1.2
206026_s_at	TNFAIP6	2.1	4.6	4.3	4.0	10.8	0.9	0.9	11.4	6.6	5.6	1.1	1.0	6.3	2.7	5.2
227345_at	TNFRSF10D	1.0	1.0	3.4	2.5	3.2	0.9	1.2	5.6	5.5	2.4	0.9	1.2	4.8	5.3	2.7
204932_at	TNFRSF11B	1.0	1.2	1.5	1.4	1.4	1.0	0.8	4.8	2.0	2.9	1.2	1.2	7.3	1.7	2.4
218368_s_at	TNFRSF12A	0.9	1.0	2.9	2.0	2.2	1.0	1.0	3.6	2.5	2.7	1.0	1.0	1.9	2.2	2.1
203508_at	TNFRSF1B	0.9	1.4	1.2	1.8	1.9	1.0	0.7	3.0	3.1	1.9	0.9	0.9	6.1	5.4	1.3
214581_x_at	TNFRSF21	1.3	1.3	2.6	2.1	2.6	1.4	1.1	8.3	7.9	7.4	0.9	1.0	10.2	7.9	5.9
210314_x_at	TNFSF13	1.1	0.9	1.3	1.0	1.0	1.0	1.0	2.7	2.4	1.7	1.1	1.0	3.3	2.7	2.5
48531_at	TNIP2	1.1	1.1	1.7	1.5	1.7	1.1	1.1	3.6	3.6	2.5	1.0	1.0	4.2	4.1	2.2
230398_at	TNS4	0.8	0.9	2.2	1.7	1.6	1.0	0.7	4.2	3.4	3.2	1.0	0.8	2.4	1.5	2.2
221218_s_at	TPK1	1.0	1.2	0.9	1.1	0.8	1.1	1.2	3.6	4.1	3.4	0.8	1.1	4.6	4.3	3.9
202241_at	TRIB1	1.0	1.2	5.5	4.4	4.1	1.0	0.9	3.9	3.7	3.0	1.1	1.1	3.8	3.2	2.6
221627_at	TRIM10	1.2	1.2	1.9	1.2	1.5	0.9	1.0	8.3	5.6	3.7	0.9	1.0	16.5	6.4	3.4
36742_at	TRIM15	0.9	1.2	2.0	1.2	1.3	1.0	1.2	5.6	4.4	3.1	1.1	0.9	7.4	2.4	1.8
1553079_at	TRIM40	0.9	1.0	1.1	1.2	1.5	0.9	1.1	4.6	7.1	4.8	0.9	1.0	6.9	6.9	5.4
231403_at	TRIO	0.6	1.7	2.9	2.7	2.6	0.8	1.2	4.1	3.4	2.4	1.9	0.9	2.9	1.9	1.2
218693_at	TSPAN15	1.3	0.9	1.2	1.0	1.1	1.0	1.1	2.0	2.1	1.8	1.0	1.2	4.2	2.3	2.2
210652_s_at	TTC39A	0.9	1.0	1.0	1.0	0.9	0.9	0.8	1.7	1.2	1.0	0.8	1.1	6.8	3.2	2.5
201531_at	TTP	1.0	1.0	7.7	8.0	7.7	1.1	0.9	13.8	12.4	7.8	1.2	0.9	15.6	10.2	6.2
209118_s_at	TUBA1A	0.9	1.4	0.9	1.6	1.6	0.9	1.0	1.1	1.4	1.1	1.0	0.9	3.5	3.9	1.9
209340_at	UAP1	1.1	1.0	1.8	1.5	1.7	1.0	0.9	3.1	3.4	3.0	1.0	1.0	3.4	3.0	2.3
238462_at	UBASH3B	1.0	0.7	2.5	1.4	1.3	1.4	0.8	10.8	5.3	4.1	0.8	0.9	3.9	3.3	4.2
204881_s_at	UGCG	0.6	1.3	3.0	2.4	2.5	1.0	1.3	6.2	4.9	3.9	0.8	1.4	2.5	2.8	3.0
215125_s_at	UGT1A1/A3-A10	1.0	1.2	1.1	0.9	1.2	0.9	1.0	2.9	3.2	3.4	1.1	0.9	4.3	1.7	4.5
238542_at	ULBP2	1.1	1.0	2.5	1.8	1.8	1.1	0.9	3.6	3.4	3.0	0.9	1.2	4.4	4.8	4.9
220370_s_at	USP36	0.9	1.3	2.8	3.6	4.1	0.9	0.9	1.3	1.1	1.0	0.9	1.0	0.7	0.8	0.8
209822_s_at	VLDLR	0.8	1.0	1.7	1.2	1.1	1.1	0.8	4.0	4.2	3.2	0.8	1.0	4.4	5.3	2.9
235023_at	VPS13C	1.0	1.2	1.8	1.2	1.4	1.0	1.0	2.7	2.3	1.5	1.1	1.1	3.4	2.7	2.0
200628_s_at	WARS	1.2	1.0	1.6	1.3	1.4	1.1	0.9	3.2	3.2	3.7	1.1	0.8	4.6	3.0	2.8
216074_x_at	WWC1	1.2	1.4	2.3	1.8	2.3	1.1	0.9	7.3	5.6	4.1	1.0	1.1	3.7	2.4	2.3
241994_at	XDH	1.0	1.1	1.6	1.5	1.7	1.0	1.3	7.2	3.3	2.5	1.0	0.9	2.1	1.4	1.4
227020_at	YPEL2	0.9	1.0	0.8	0.8	0.7	0.9	1.2	4.9	5.1	2.7	1.0	1.0	14.8	8.2	3.5
218810_at	ZC3H12A	1.3	1.5	3.4	3.9	3.4	1.0	1.0	1.8	2.0	2.1	0.9	0.8	2.3	1.9	1.8
220104_at	ZC3HAV1	1.3	1.3	1.4	1.2	1.8	1.0	1.1	3.3	2.9	6.1	1.1	1.1	2.2	2.4	2.4
1569369_at	ZFYVE28	0.8	1.1	1.6	1.1	1.2	1.0	1.1	3.4	3.9	2.4	1.1	0.8	3.9	3.7	1.7
1556545_at	---	1.2	1.5	2.1	4.5	4.8	1.2	1.0	1.4	1.1	1.0	1.1	0.9	1.2	1.2	0.8
1559037_a_at	---	1.0	0.9	1.8	1.3	1.1	1.1	1.0	4.3	3.5	3.1	0.9	0.8	3.0	2.0	2.1
1559663_at	---	0.6	1.1	4.5	1.5	2.1	0.8	1.3	3.7	2.7	1.7	1.1	1.0	1.3	1.7	1.3
1562056_at	---	1.0	1.2	6.2	1.9	3.9	1.0	1.2	3.2	2.1	2.0	1.1	1.3	1.4	1.4	1.6
1562529_s_at	---	1.0	1.2	1.1	1.6	1.2	0.7	0.8	1.6	2.3	1.1	1.1	1.1	2.8	5.7	1.8
212444_at	---	0.9	0.9	2.8	2.1	1.9	1.0	1.0	4.8	5.1	4.2	0.9	1.0	3.3	5.1	4.1
214967_at	---	0.8	1.1	6.7	4.6	5.2	0.8	0.9	6.0	5.2	3.1	1.3	1.3	9.2	5.1	2.3
221159_at	---	1.4	1.1	4.5	1.8	1.7	0.9	1.1	4.6	4.4	4.5	1.1	0.8	6.3	3.8	2.7
226542_at	---	1.2	0.8	2.3	1.7	1.4	1.0	0.7	6.7	5.5	3.7	1.0	1.1	12.1	8.2	6.7
226756_at	---	1.0	1.1	3.0	2.5	3.4	1.2	1.3	6.8	5.9	4.7	1.0	1.3	5.6	2.7	3.7
228919_at	---	0.7	1.7	1.5	1.3	2.8	0.6	0.9	1.0	3.8	1.5	1.1	1.0	2.1	7.3	2.8
229242_at	---	1.1	1.1	1.7	0.9	1.0	0.9	0.9	1.9	1.7	1.9	0.8	1.1	2.0	4.0	4.3
230333_at	---	1.9	3.0	8.8	7.8	8.0	0.8	1.3	5.8	5.8	4.5	1.2	1.2	4.0	2.8	3.4
230357_at	---	1.0	1.2	1.4	1.6	1.6	1.2	1.0	3.4	2.2	1.6	0.9	1.0	3.5	2.8	3.2
230503_at	---	1.1	1.2	5.3	5.2	4.0	0.8	1.1	5.4	3.4	2.2	0.9	1.0	4.3	4.2	2.3
230688_at	---	1.1	1.1	4.0	2.5	1.7	0.6	1.0	2.7	3.1	1.5	0.8	1.0	2.0	2.1	1.2
230711_at	---	0.9	0.8	4.0	3.7	2.9	0.9	0.7	6.7	5.2	3.1	0.9	0.8	5.4	4.5	3.7
231035_s_at	---	0.9	1.1	2.8	2.8	3.8	1.1	1.1	3.7	3.3	2.8	0.9	1.2	3.9	3.3	4.0
232174_at	---	1.1	1.1	3.5	3.5	3.2	1.0	0.8	1.6	1.6	1.4	1.0	1.0	2.0	2.7	1.5
232202_at	---	1.2	1.1	5.1	2.9	2.7	1.0	1.4	9.2	8.7	5.8	1.0	0.8	10.4	5.5	5.8
232290_at	---	1.2	0.7	4.8	2.7	2.4	1.1	0.8	4.0	4.1	3.7	1.0	0.8	2.6	4.8	3.8
232369_at	---	1.0	1.2	3.8	3.1	2.4	0.9	0.9	2.9	2.3	1.7	1.0	1.1	3.1	2.5	1.8
232451_at	---	0.7	1.2	1.0	1.4	1.7	1.1	0.8	2.3	2.5	0.9	0.9	1.3	4.7	4.7	2.7
232472_at	---	1.0	1.2	4.5	3.2	2.4	0.9	0.8	3.2	2.5	2.2	1.0	1.0	2.5	3.2	2.2
232584_at	---	1.1	1.0	3.6	3.2	2.8	1.0	1.1	1.9	1.9	1.6	1.1	1.1	1.2	1.5	1.1
232784_at	---	1.0	1.3	2.3	2.6	2.8	0.9	0.9	3.7	3.2	2.0	1.2	1.0	5.9	4.7	1.9
232883_at	---	1.2	1.3	1.0	1.1	0.9	0.9	1.3	1.7	2.0	1.0	1.1	1.0	3.4	2.8	1.0
232978_at	---	0.9	1.3	4.4	1.9	3.0	0.7	1.0	3.0	1.5	1.3	0.9	1.0	2.9	3.1	1.0
233309_at	---	0.8	1.0	3.1	1.5	1.9	0.8	1.2	4.5	3.4	2.0	1.1	0.9	6.7	1.9	1.4
233388_at	---	0.8	1.4	2.2	2.1	1.5	1.1	0.7	4.8	2.5	1.7	1.0	1.1	6.4	4.5	3.4
233506_at	---	0.7	1.1	1.6	1.7	1.7	0.8	1.1	4.0	3.5	2.6	1.0	1.0	1.9	1.9	2.3
233771_at	---	0.9	1.2	6.2	4.8	3.9	0.8	0.9	2.3	2.1	1.6	0.9	0.8	1.7	1.6	1.2
235295_at	---	1.5	1.5	1.9	2.2	2.2	1.2	1.0	3.4	3.0	2.8	0.9	1.2	2.0	2.0	1.9
235419_at	---	0.9	1.3	5.8	5.0	5.7	1.0	1.0	1.9	1.9	1.6	0.9	1.2	1.4	2.5	1.9
235680_at	---	1.1	1.1	3.7	3.1	2.8	0.9	0.8	2.9	2.0	1.8	1.1	0.9	3.7	2.0	1.7
235756_at	---	1.0	1.0	2.0	1.5	1.4	1.4	0.7	4.4	2.6	1.9	1.1	1.2	3.9	3.0	2.0
236067_at	---	0.8	1.1	2.9	2.5	1.7	1.0	1.5	3.3	4.0	2.8	1.2	1.0	4.2	3.5	2.1
236439_at	---	1.0	1.7	1.7	1.4	1.9	0.9	1.0	3.6	2.7	2.0	0.9	0.9	3.9	2.1	1.8
236685_at	---	0.9	1.1	2.6	2.3	2.1	0.9	1.0	2.6	2.7	1.7	1.0	0.8	3.4	2.9	1.7
236699_at	---	1.0	0.9	3.9	2.7	2.7	1.1	1.0	3.1	2.9	2.0	1.0	0.9	3.7	3.0	1.8
237299_at	---	0.9	0.9	1.4	1.2	1.1	0.9	1.1	3.5	3.3	2.1	1.1	0.9	3.0	1.9	1.7
237616_at	---	0.8	1.1	1.8	1.5	1.4	1.0	0.9	2.1	2.0	1.1	1.1	0.9	3.6	2.9	1.4
239102_s_at	---	0.9	0.9	4.1	3.8	3.1	1.1	1.3	2.2	2.8	2.7	1.1	1.0	1.8	1.8	2.4
239258_at	---	0.9	0.8	4.0	2.4	1.9	0.8	1.1	15.2	9.7	6.2	1.0	1.0	15.3	7.1	7.4
239269_at	---	1.1	1.1	2.0	1.5	1.7	0.9	1.2	3.5	3.1	2.7	1.0	1.0	3.4	2.9	1.6
239331_at	---	0.8	1.0	9.1	7.6	7.9	0.9	1.1	4.8	5.0	3.9	0.8	0.9	2.8	4.3	3.1
239448_at	---	0.9	1.2	3.9	3.2	2.3	1.0	1.0	3.0	2.6	1.4	0.8	0.9	1.4	2.2	1.1
239455_at	---	1.2	1.0	1.0	1.1	1.1	1.1	1.3	2.4	2.6	1.7	0.9	0.8	5.2	2.0	1.2
239808_at	---	1.1	1.1	3.8	1.4	1.1	0.9	0.7	8.1	4.4	3.5	1.0	1.1	7.2	5.4	5.9
239845_at	---	0.6	1.2	3.9	3.6	7.3	0.8	0.9	1.5	1.6	1.0	1.2	1.1	1.6	1.8	1.2
240960_at	---	1.0	1.4	3.3	2.7	4.0	0.9	1.0	2.9	2.3	1.8	1.2	1.0	2.6	2.3	1.6
241497_at	---	1.0	1.1	4.0	2.1	1.6	0.9	1.1	1.9	1.8	1.2	1.1	1.0	1.3	1.5	1.4
241613_at	---	0.9	1.1	2.1	1.3	1.7	0.8	1.0	4.4	3.9	1.9	1.1	1.1	6.9	5.4	1.7
241722_x_at	---	0.9	0.9	3.6	3.1	4.0	0.9	1.0	2.5	2.5	2.9	1.0	0.9	1.9	2.0	3.7
241916_at	---	0.9	1.6	7.4	5.7	7.7	1.0	0.9	9.0	8.0	5.0	1.1	1.1	7.6	4.2	3.3

		4 h					10 h					20 h				
**Probeset**	**GeneID**	***invA***	***invA***	**wt**	**wt**	***Δasd***	***invA***	***invA***	**wt**	**wt**	***Δasd***	***invA***	***invA***	**wt**	**wt**	***Δasd***
242126_at	---	1.4	1.1	2.8	3.2	2.1	1.1	0.7	1.4	1.3	1.0	1.4	1.0	1.9	2.3	1.2
242457_at	---	1.2	1.1	4.4	3.0	2.7	1.1	1.0	1.9	2.9	1.8	1.0	0.7	1.6	2.7	1.1
242868_at	---	1.0	0.9	5.6	4.1	3.8	0.8	0.8	7.4	6.8	5.2	0.9	1.0	7.2	6.8	4.5
242907_at	---	2.1	3.1	1.2	1.3	1.6	1.1	1.7	1.3	1.5	1.6	1.0	2.2	7.2	5.1	3.5
243509_at	---	1.1	1.5	1.6	2.6	1.9	1.0	1.2	2.4	2.4	1.5	0.9	0.9	3.5	3.0	1.8
243858_at	---	1.3	2.5	1.8	2.2	2.0	1.2	0.9	4.9	3.9	2.4	0.8	0.8	2.8	2.5	1.7
244022_at	---	1.1	1.2	2.7	1.8	2.0	1.0	1.1	2.8	3.6	2.6	0.8	1.1	2.7	3.0	2.1
244530_at	---	0.9	1.0	1.3	1.2	1.2	1.1	1.3	3.0	1.8	1.4	0.9	0.9	15.3	4.5	3.6
244536_at	---	0.9	1.0	2.9	3.6	2.6	0.9	0.8	1.4	1.4	1.2	1.0	1.0	1.1	1.4	1.1
