# Supplementary material for: Salmonella Modulation of Host Cell Gene Expression Promotes Its Intracellular Growth
Source: PLoS Pathog. 2013 Oct 3;9(10):e1003668. doi: 10.1371/journal.ppat.1003668 (PMC3789771; doi:10.1371/journal.ppat.1003668)
Supplement: Table S2 — Transcription binding sites in genes whose expression increased at least 4-fold 4 h after infection1. (PDF) [file ppat.1003668.s016.pdf]

Table S2: Transcription binding sites in genes whose expression increased at least 4-fold 4 h after infection[1]

Transcription Factor	Matrix-ID	z-Score	p-Value	Sample Average	Sample StDev	Background Average
V$CREB_Q2	M00177	5.69657	0.000000006	0.888133	0.056356	0.840576
V$CREB_01	M00039	5.12732	0.000000144	0.915694	0.065138	0.861159
V$CREB_Q4	M00178	5.08473	0.000000180	0.890082	0.059943	0.843390
V$CREBP1_Q2	M00179	4.82576	0.000000681	0.872625	0.062210	0.831952
V$ATF_01	M00017	4.33748	0.000007086	0.863570	0.065751	0.822283
V$CREBP1CJUN_01	M00041	4.20164	0.000013086	0.900789	0.069761	0.852650
V$CREB_02	M00113	3.88629	0.000050385	0.892368	0.048196	0.860612
V$NFKAPPAB50_01	M00051	3.81528	0.000067328	0.877879	0.064581	0.837096
V$TAXCREB_01	M00114	3.79223	0.000074000	0.814254	0.044856	0.784025
V$CREBP1_01	M00040	3.34728	0.000405450	0.854335	0.063792	0.814704
V$TATA_01	M00252	3.11767	0.000906489	0.881359	0.047870	0.853336

[1]The -950 to +50 nucleotides region around the transcription start sites of genes whose expression increased at least 4-fold in Henle-407 cells 4 h after infection with wild type *S.* Typhimurium was analyzed using PSCAN (http://159.149.160.51/pscan/) for the presence of transcription factor binding sites (p-Value < 0.001; sample size: 275 genes).
