# Supplementary material for: Salmonella Modulation of Host Cell Gene Expression Promotes Its Intracellular Growth
Source: PLoS Pathog. 2013 Oct 3;9(10):e1003668. doi: 10.1371/journal.ppat.1003668 (PMC3789771; doi:10.1371/journal.ppat.1003668)
Supplement: Table S3 — Transcription binding sites in genes whose expression increased at least 4-fold 10 h or 20 h after infection1. (PDF) [file ppat.1003668.s017.pdf]

Table S3: Transcription binding sites in genes whose expression increased at least 4-fold 10 h or 20 h after infection[1]

Transcription Factor	Matrix-ID	z-Score	p-Value	Sample Average	Sample StDev	Background Average
V$STAT3_01	M00225	5.45721	0.000000020	0.740905	0.044974	0.727700
V$STAT1_01	M00224	4.86047	0.000000496	0.761326	0.047528	0.749272
V$STAT_01	M00223	4.65422	0.000001438	0.896872	0.048058	0.883862
V$NFKAPPAB65_01	M00052	3.75149	0.000079781	0.862484	0.056143	0.851240
V$AP1_Q6	M00174	3.40031	0.000313411	0.887850	0.045857	0.879007
V$STAT5B_01	M00459	3.38061	0.000340827	0.808563	0.049307	0.798259
V$STAT5A_01	M00457	3.282	0.000483816	0.837026	0.049666	0.827533
V$NFKAPPAB_01	M00054	3.19382	0.000655057	0.876067	0.051535	0.867134
V$NFKB_C	M00208	3.13344	0.000811715	0.830389	0.049680	0.821640

[1]The -950 to +50 nucleotides region around the transcription start sites of genes whose expression increased at least 4-fold in Henle-407 cells 10 h or 20 h after infection with wild type *S.* Typhimurium was analyzed using PSCAN (http://159.149.160.51/pscan/) for the presence of transcription factor binding sites (p-Value < 0.001; sample size: 275 genes).
