# Supplementary material for: Salmonella Modulation of Host Cell Gene Expression Promotes Its Intracellular Growth
Source: PLoS Pathog. 2013 Oct 3;9(10):e1003668. doi: 10.1371/journal.ppat.1003668 (PMC3789771; doi:10.1371/journal.ppat.1003668)
Supplement: Table S4 — Predicted STAT3 binding sites in genes that are at least 4-fold increased 10 h or 20 h after infection1. (PDF) [file ppat.1003668.s018.pdf]

Table S4: Predicted STAT3 binding sites in genes that are at least 4-fold increased 10 h or 20 h after infection[1]

Probeset	GeneID	Score	Position	Sequence	Strand
206513_at	AIM2	0.890126	-482	GGGCAATTCCCGGAATCGGGG	-
238725_at	IRF1	0.888048	-130	CGTCATTTCGGGGAAATCAGG	-
207847_s_at	MUC1	0.884589	-509	GGGCTATTCCGGGAAGTGGTG	+
216598_s_at	CCL2	0.862372	-145	GTGGATTTCCAGGAAGTAGGA	-
203882_at	IRF9	0.850041	-17	CGTGATTTCTGGGAAAAGGCC	+
231270_at	CA13	0.847946	-29	GAGGACTTCCAGGAAGTGAAT	-
208747_s_at	C1S	0.841093	-229	AGTGATTTCTGGGAAAAGGCG	-
202437_s_at	CYP1B1	0.839695	-345	CTGGCTTTCCGGGAAGCAAGC	+
210652_s_at	TTC39A	0.838805	-562	TCCTATTTCCCAGAAGTTAGT	-
205315_s_at	SNTB2	0.831947	-120	CTGCACCTCCCGGAAGCGGCT	+
207574_s_at	GADD45B	0.829826	-273	CCTGATTTCCCGGCAGCCGAG	-
201531_at	ZFP36	0.824660	-105	GGGCGCTTCCCGGACGCGCGC	-
200697_at	HK1	0.822368	-457	CCTCATTTCCGAGGAATGGGA	+
234725_s_at	SEMA4B	0.820632	-64	CCGTGTTTACGGGAATTGTCC	-
213238_at	ATP10D	0.820044	-7	TTTTTTTTCCCGGAAGGCAAA	+
213506_at	F2RL1	0.818622	-104	GCGCGAGTCCGGGAAGCGGAG	+
224701_at	PARP14	0.816328	-32	TTTCGTTTCCTGGAAAACTCC	-
206394_at	MYBPC2	0.813996	-494	AGGAGTTTTCGGGAAACCGGA	-
1557236_at	APOL6	0.813756	6	GGGCTCTTCCAGGAAATCATG	-
202086_at	MX1	0.813454	-277	CTGCATTTCCTGCAAGTCCGT	-
205239_at	AREG	0.811855	-489	GGCCACTTCACAGAAATGGGT	+
205239_at	AREG	0.811855	-491	GGCCACTTCACAGAAATGGGT	+
220187_at	STEAP4	0.811299	-83	ACTTGTTTCCTGGAAGAGTTC	-
214978_s_at	PPFIA4	0.808285	-635	TGCTGTATCAGGGAAATGGAG	+
218130_at	C17orf62	0.808129	-90	GCGGATTTCCGGGATCCGGGC	-
205569_at	LAMP3	0.807143	-907	CGCGGTTTGCGGGAAGCAACA	-
212268_at	SERPINB1	0.806853	-178	CGTGGCTTCCGGGCAGCGCCC	-
210001_s_at	SOCS1	0.806617	-625	CGGGCTTCCCGGGAAGCGGCG	-
229521_at	FLJ36031	0.803548	-304	GGAGATTTCCCTGAAGGATGT	-
48531_at	TNIP2	0.801486	-351	GGAAAGTTCCGGGAAGGGCGG	+
205579_at	HRH1	0.801154	-605	TGGCAATTACCAGAAATCCAA	-
205896_at	SLC22A4	0.800605	-497	AGAAGTTTCCCGGGAACCGAC	+
202238_s_at	NNMT	0.799596	-574	ACTTAATTCCAGGAACTGGCC	-
229004_at	ADAMTS15	0.798302	-134	CGGCAGTTCCCAGAAAGGAGA	-
201170_s_at	BHLHE40	0.796892	-683	GGGCAATTCCTGGACGCGAGG	+
222662_at	PPP1R3B	0.794846	13	GCTGGGTTCCCGGAGATGTCG	-
204988_at	FGB	0.789839	-262	TCTTGTTTCTGGTAAATTAAT	-
205302_at	IGFBP1	0.789763	-653	TCTCATTTCTCGGATATTCTC	-
231861_at	LRP10	0.788820	-561	AGTAATTTCCTGGAGGTCACA	+
202948_at	IL1R1	0.788789	-545	TTTGATTTCCTGAAAGTATCC	+
206584_at	LY96	0.787318	-123	TCTCATTTACAGTAAGTGTTT	+
228648_at	LRG1	0.785654	-180	AACAATTTCCAGGAAGCAATT	+
210517_s_at	AKAP12	0.785414	-300	GCACATTTCCGGTAACAGCCT	+
201473_at	JUNB	0.784194	-318	GTGGACTCCAGGGAAATCATC	+
202446_s_at	PLSCR1	0.783393	-651	ACCAGTTTCACGGAAGAGGAT	-

Probeset	GeneID	Score	Position	Sequence	Strand
227697_at	SOCS3	0.782949	-70	CCCCGATTCCTGGAACTGCGC	-
226694_at	AKAP2/PALM2-AKAP2	0.782846	-440	GAGCGACTCCCGGAACTGGTT	-
204279_at	PSMB9	0.782494	-350	TTCGCTTTCCCCTAAATGGCT	-
214461_at	LBP	0.782281	-586	GGGCAAGTCCCTGAAATTGAA	+
203887_s_at	THBD	0.781704	-533	GTGGACTTCCCCGAAGACAGG	-
224916_at	TMEM173	0.780914	-96	GGCTCTTTGCAGGAAATGGCC	-
213716_s_at	SECTM1	0.780721	-645	ACCAAACTCCGGGAAACCCTC	-
206034_at	SERPINB8	0.779898	26	AGTCATCTGCCGGAAGCCTTG	+
209875_s_at	SPP1	0.779278	-178	TTTCATTTACGGGATGGGCAT	-
243296_at	NAMPT	0.779014	-661	GAGTATTGCCGGGAAGGAAAA	-
238439_at	ANKRD22	0.777348	-491	TTTCAATTCTGGGAAAGTTTG	+
210732_s_at	LGALS8	0.776583	-922	CAGGGTTTCCCTCAAGTGATC	-
204393_s_at	ACPP	0.776158	-51	TAAAGTTTCCAGGAAGCGTAG	-
229865_at	FNDC3B	0.775823	-403	CGGAGATTCCGGGAGGGGGAG	-
212543_at	AIM1	0.775204	29	AGGAATTTCCCGGCGAGGAAT	-
201645_at	TNC	0.774563	-365	TCCTCTTTCCAGGAACTGGGC	+
207850_at	CXCL3	0.774394	-5	GGGAAATTCCCGGAGCTCCAG	-
209774_x_at	CXCL2	0.774394	-3	GGGAAATTCCCGGAGCTCCAG	-
212185_x_at	MT2A	0.774142	-182	GGTCAGTTCCCTGAAGGCGGC	-
210056_at	RND1	0.773634	-846	GCTTGAATCCGGGAGATGGAG	+
209189_at	FOS	0.773491	-304	AGGGATTGACGGGAACTGCTC	-
208892_s_at	DUSP6	0.773229	-907	GCTCCTTGCCCAGAAATCATT	+
225524_at	ANTXR2	0.772873	-399	TGTCATTTCCCTGCAAGCTCC	-
205650_s_at	FGA	0.772404	-132	GTTGGTATCCCAGAAATTCTT	-
209930_s_at	NFE2	0.771723	-666	TCCCCTTTACGGGAGGTGAAT	+
204567_s_at	ABCG1	0.770759	-852	GGCCATTTCCTGGTATCCATG	-
220358_at	BATF3	0.769586	-598	GGTGAATTCCCGGAGCGGAGG	-
223467_at	RASD1	0.769226	-521	TCTTGTTCCCGGGAGGCGACC	-
201466_s_at	JUN	0.768552	-752	TTGTATTGCCCGAAACTGGAC	-
221218_s_at	TPK1	0.768359	-73	CTCCGGCTCCCGGAAGCGGGC	+
227034_at	ANKRD57	0.767833	-814	GGCAAAGACCGGGAAGTCGTT	+
223218_s_at	NFKBIZ	0.765493	-261	GGGAATGTCCGGGACTCGGGT	+
223434_at	GBP3	0.764901	-21	ACTTCTTTCAAGGAAGTGACT	-
202998_s_at	LOXL2	0.763791	-155	CTGAGATGCCGGGAAGGGGGC	-
213275_x_at	CTSB	0.763632	-706	GTTTATTACCGGGAGGCCCTG	-
209351_at	KRT14	0.763527	-567	GTGTATTTCAGGGATGGGACA	+
227020_at	YPEL2	0.763027	-793	TTTTAATTCGCCGAAATTACT	+
209498_at	CEACAM1	0.762554	-505	CTGCAGCTCCCGGATATGGAG	-
227345_at	TNFRSF10D	0.762450	-93	CCTGAGTGCCTGGAAGTGACT	+
204326_x_at	MT1X	0.761800	-244	GGCAATGTCAGGGAAATTGGG	+
221541_at	CRISPLD2	0.760484	-623	CCTGGATTCCTGGGAACGCGT	+
227099_s_at	C11orf96	0.758911	-144	CGGGGGTTCCGGGAGAAGCCG	-
216615_s_at	HTR3A	0.757860	-320	CTGTAGTTACGGGAAGGGTAA	-
206461_x_at	MT1H	0.757854	-276	GGTAATCTCAGGGAACTGGGC	+
204932_at	TNFRSF11B	0.757770	-349	TGCGAACTCCCCGAAAAGGGC	+
202357_s_at	CFB	0.757401	-317	TGGAATTTCCCAGTTATGAAA	+
202902_s_at	CTSS	0.757212	-805	AGTGATTTGTGGGAGGTGTGT	-
214453_s_at	IFI44	0.756706	-207	TTGCATTTTCCTTAAATAAAA	+
235740_at	MCTP1	0.754870	-155	ATGTCTTTCTGGGAGATGTTG	+

Probeset	GeneID	Score	Position	Sequence	Strand
220416_at	ATP8B4	0.754725	-880	AGGAATTTTCCAGAAGTTGCA	-
219630_at	PDZK1IP1	0.754539	-200	TCCCTTTTCCTGGCAGTGGGG	-
220334_at	RGS17	0.754130	-502	TGGGGGTTCAAGGAAGTGTGA	-
202800_at	SLC1A3	0.753958	-641	GCTTATTTCATGGAAAAAAAA	+
238018_at	FAM150B	0.753811	-182	CGCCATGGCCGGGAGGTGGAG	+
202619_s_at	PLOD2	0.752396	-695	TCTAATTTCACGAAAATTTAG	+
202638_s_at	ICAM1	0.751976	6	TCCCGTTTCCCGGCCAGGCTA	-
219862_s_at	NARF	0.751468	-590	GCTGGTTTCCGTTAAGCTCTT	+
213988_s_at	SAT1	0.750928	-620	TTGGATTTCCCAATAATCTTT	-
206026_s_at	TNFAIP6	0.750090	-355	TGTCAATTATGGGATATGGGG	-
227868_at	LOC154761	0.749272	-251	TGGGCTGTCCCGGAGGCGGGG	-
226140_s_at	OTUD1	0.749130	-804	GCTTGAGCCCGGGAAGTCAAG	+
214581_x_at	TNFRSF21	0.748105	-683	TTTGAACTCCTTGAAATGTAT	-
205220_at	GPR109B	0.747770	-327	TGAGGTTTTCCAGAAACACTA	-
201060_x_at	STOM	0.747675	-156	AGTCACTTCCCGCTAGTTCTG	-
219634_at	CHST11	0.747355	-924	TCGTATGTCAGGGAAAAAAAA	-
209732_at	CLEC2B	0.747218	-768	TGGCAATTCTTGTAAGTCTGA	-
205651_x_at	RAPGEF4	0.746964	-452	ACAGATATCCCAGAAGTTCTC	+
228640_at	PCDH7	0.745659	-58	GCGCGATTCAGGGAGGAGATT	-
217165_x_at	MT1F	0.745404	-342	CTGGGACTCCAGGAAAGGCTT	+
203153_at	IFIT1	0.744807	-446	GCTTATTTCCGTCAAGCTGAA	+
1555832_s_at	KLF6	0.744175	-599	GGGGGCTTCCCGGTCGCCAAC	-
205027_s_at	MAP3K8	0.743695	-887	GTGGATTTCGGGAGAGTGGCG	+
214660_at	ITGA1	0.743621	-138	GTCGAATTCCTCTAAATGCGC	-
216074_x_at	WWC1	0.743405	-419	CGCGCTGCCCCGGAAACGGGA	-
212812_at	SERINC5	0.742926	-168	CGCCCCAACCGGGAAGTGATC	+
210538_s_at	BIRC3	0.742873	-103	CTGGAGTTCCCCTAAGTCCTA	+
212657_s_at	IL1RN	0.742810	-330	TCCCAATGCCTGGAAGAGTGC	+
1562031_at	JAK2	0.741992	-696	CGCTGTTTCGTGGCAATGCGC	-
224797_at	ARRDC3	0.741606	-825	TCTTCCTTCGAGGAAGTGATC	+
212230_at	PPAP2B	0.740018	-389	TTGTATTACCCTGAAAATATA	-
229074_at	EHD4	0.739167	-863	GCTTGAACCCGGGAAGCGGAG	+
219612_s_at	FGG	0.738789	-449	TCACTATTCCCAGGAATGAAT	-
238542_at	ULBP2	0.738781	-128	AGGGACGCCCGGGAAGGGGAG	+
217996_at	PHLDA1	0.738225	-95	AGGAGTTTCCGGGGCTCGGGT	+
1555786_s_at	C14orf34	0.737714	-471	CCTACTTTCAGGGAAAGCAGG	-
204908_s_at	BCL3	0.737659	-570	TGGCATCTCTGGGACGGGGTT	-
223784_at	TMEM27	0.737135	-115	AGAGGTTTCCTTGACATGCAC	-
228758_at	BCL6	0.736378	-496	ACCAATTTCCAGCAAGTTCTG	-
225275_at	EDIL3	0.736129	-469	AGGAAGTTCAGGGGAATCCTT	+
1568768_s_at	LOC100302650	0.736124	-115	AGCAATTTCCCCCAAGGGGCA	+
202834_at	AGT	0.735261	-528	TGAAATTACCCAGGAATGGAA	-
221840_at	PTPRE	0.734753	-203	TGCCCCTTCCGGGGAGGCCTG	-
241359_at	TLCD2	0.734687	-524	CAGGTTTGCCGGGATATGGTA	+
208937_s_at	ID1	0.733753	-642	CTGGGTGGCTGGGAAGTGAAC	-
209457_at	DUSP5	0.733435	-332	TTCAGCTTCACGGACGTGGGC	-
214435_x_at	RALA	0.733124	-3	CCCAGCGCCCCGGAAGTGATC	+
221627_at	TRIM10	0.732739	-840	TAGCATTGCTCGGAAGACAGA	+
203963_at	CA12	0.732394	-859	CAAGCATTCCGGGAGTTGTTT	-

Probeset	GeneID	Score	Position	Sequence	Strand
1552703_s_at	CARD16/CASP1	0.732144	-632	TGGGATTTGCCTGTAATTTTA	+
210029_at	IDO1	0.731187	-374	TTGTGTTTCCGGGCTGCTGAA	+
210095_s_at	IGFBP3	0.730997	-411	GCCAGTTTCCCCGACACCGGC	+
238462_at	UBASH3B	0.730702	-171	GGTGACTCTCGGGAGGTGAAG	+
208965_s_at	IFI16	0.730269	-78	AGCCCTTGCCAGGAAACTGTT	+
201313_at	ENO2	0.729871	-571	TTTGATCTCCTGGAGGTAGAA	-
212067_s_at	C1R	0.729567	-79	GGACCATTCCCGGAGGAATGT	-
204627_s_at	ITGB3	0.728972	-531	TTTGGTTTTCCGGTAGACTAC	-
205513_at	TCN1	0.728695	-188	TTTGCCTTCCGTGCAGTGAAG	-
209276_s_at	GLRX	0.728578	1	TTGCATTCCTGGGCATTGCTA	+
205767_at	EREG	0.728462	-2	GGAAATATCAGGCAAGTGAGC	-
232277_at	SLC28A3	0.728357	-903	TGAGGTTTGTGGGAACTCAAG	-
231880_at	FAM40B	0.727671	-383	GGTGGTAACAGGGAAACGGAG	+
227452_at	LOC100499467	0.726651	-115	TACGACTCCCAGGAAGCCGTT	+
213069_at	HEG1	0.726629	-897	ATTTGTTTACATGAAATCTTT	+
211924_s_at	PLAUR	0.726462	-402	GTGCAATGCCTGGAATAGCTG	+
209493_at	PDZD2	0.725896	-268	GGCCTTCTCCAGGAAGTTTCC	-
206239_s_at	SPINK1	0.725570	-486	ACTCAGTTCCCTAAAGCGTGT	-
211368_s_at	CASP1	0.724952	-786	TGCAATTTCCCTCAAAAATAA	+
212845_at	SAMD4A	0.724731	-249	CTGGATTTCGGCGAACCCAAC	+
218113_at	TMEM2	0.724414	-895	TGCCAAATCCGGGCCGTCGTC	-
217995_at	SQRDL	0.723979	-467	CTGTGTTTCTGGGCAGCTGTG	+
203780_at	MPZL2	0.723139	-254	CCAGGTCTCCTGGAAAGCTAC	+
221773_at	ELK3	0.722468	-238	CGCGCTTCCCGGGACACCGGG	-
213006_at	CEBPD	0.722360	-631	CCCGGATTCCCCGAGGCGCCC	+
218451_at	CDCP1	0.722208	-538	TCACAATTCAACGAAGTGGTG	+
212122_at	RHOQ	0.720964	-328	CCGCAGCGCCCGGAAGCGTCC	-
219155_at	PITPNC1	0.720955	-45	TTTTAATTTTTGGAAATCTCT	+
212641_at	HIVEP2	0.720819	-173	TCGGGCCACCGGGAAGGGGCT	-
217523_at	CD44	0.720752	-108	GCCGGATTCAGAGAAATTTAG	+
214022_s_at	IFITM1	0.720741	14	GTGTATTTCTCTTAAGTTTCT	-
225557_at	CSRNP1	0.720739	-822	CTTGGTTGCCGGGGCATGTCG	+
217763_s_at	RAB31	0.720734	-720	GCTCATTTCCCAGTTAGGAAC	+
213056_at	FRMD4B	0.719764	-274	TGTGGCTGCCCAGAGGTGTTT	+
204881_s_at	UGCG	0.719467	-482	GGCAGCTGCCCGGAACCCGTG	+
240304_s_at	TMC5	0.719108	-98	GCTCAATTCTCAGAAGAAAGC	+
1553079_at	TRIM40	0.719066	-706	CAGTGTTTCTAGGAAAGCATG	+
203939_at	NT5E	0.718987	-660	CCTTGCTTCTGGGACACCCTC	-
203139_at	DAPK1	0.718832	-415	GCAGGTTTCCGGAAGGCAGGG	+
203372_s_at	SOCS2	0.718672	-230	GGGCATTTCCCAGCTAACAAA	-
213413_at	STON1	0.718672	-805	AGTGACTTGCCTGGAGTCATT	+
209822_s_at	VLDLR	0.718657	-708	TTTAGTTTTCGGCAAAACGGC	+
203058_s_at	PAPSS2	0.717540	-203	CGCCTCTTCAAGGAAGTCTTC	-
230252_at	LPAR5	0.716547	-280	TGGTGTTTGCGGGGAGGTGGG	+
204364_s_at	REEP1	0.716177	-102	AGACCATTCCCCCAAATAGTC	-
218918_at	MAN1C1	0.715563	-264	GGACGTGTCCCGATAGTCGAG	+
233587_s_at	SIPA1L2	0.715550	-871	TTCAGTCTCCAGGAATGGATT	-
212298_at	NRP1	0.714801	-172	CTGTCTTTCCCAGAGACCCTG	-
208607_s_at	SAA1/SAA2	0.714528	-62	GCTGGTTTCTTGGGAGCGAAG	-

Probeset	GeneID	Score	Position	Sequence	Strand
201667_at	GJA1	0.714142	-447	TTACATTTAAAGGAAGGGAAT	+
226811_at	FAM46C	0.714089	-122	TGGCTCTTCGGGTGAATGGTT	-
206170_at	ADRB2	0.714058	-58	GGGAACTTTCGGCCAATGGCA	-
210426_x_at	RORA	0.713876	-254	GGGCAATGCCTGGAGCTGGGT	+
206090_s_at	DISC1/TSNAX-DISC1	0.712404	-232	GCCTGTCTCCGGGATCTCAGG	-
203543_s_at	KLF9	0.712371	-434	CTGAGTTACAGAGAAGTGACT	-
202833_s_at	SERPINA1	0.711589	5	CTGCACTTACCGAAAGGAGTC	-
209050_s_at	RALGDS	0.709448	-607	GGTGCTGTCAGGGAAGACAAG	+
223220_s_at	PARP9	0.709438	-312	GTGGAGTTCAGTGAAAGGGCA	-
33304_at	ISG20	0.708855	-416	TGGGGCCTCCCCGAAGGCCTG	+
200878_at	EPAS1	0.708621	-889	TGGAAGTCCTGGGAAACCCAA	-
235252_at	KSR1	0.708404	-630	TGGGGTGATCGGGAAAGGCCT	+
36742_at	TRIM15	0.707675	-51	GAGATTCTGCCGGAAGTGTGT	-
228325_at	KIAA0146	0.707563	-239	GGGGAGTTCGGGGGAGCTGCT	-
210413_x_at	SERPINB3/ SERPINB4	0.707558	-769	TGGAAATTCTGGTAGATACAA	+
209719_x_at	SERPINB3	0.707558	-711	TGGAAATTCTGGTAGATACAA	+
219371_s_at	KLF2	0.706965	-108	GTCCCCATCCGGGACGCGTTT	+
206385_s_at	ANK3	0.706952	-884	GGGAAATACATGGAAATAGAA	-
215223_s_at	SOD2	0.706812	-105	CGGGGTGTACGGCAAGCGCGG	+
202859_x_at	IL8	0.706609	-439	GAGAGTTTCTAGGAACTGTTA	-
227458_at	CD274	0.706441	-92	GCCGATTTCACCGAAGGTCAG	+
227443_at	C9orf150	0.706420	-862	TTATATGTCACAGAAATAGAG	+
218687_s_at	MUC13	0.706156	-420	ACATGTTTCCCAAAATTATTA	-
202180_s_at	MVP	0.705346	-851	CTGAGCTTCCTGGAGAAGAGG	-
238017_at	SDR16C5	0.704575	-433	GCCCATATCTGGGACAGGTCT	-
202464_s_at	PFKFB3	0.704033	-272	GGTTTCTTCCCGGACCAGTTT	+
205660_at	OASL	0.703100	-184	GGGTGTCTCCAAGAAGGGGGA	+
227314_at	ITGA2	0.703043	-940	AGCAATGTGCTGGAAATTTGT	+
226142_at	GLIPR1	0.702565	-843	TGCCGTTTCCAGAATGGGTTA	+
205020_s_at	ARL4A	0.701793	-427	GGCCAGTTCCCCGGGATGCTG	+
205921_s_at	SLC6A6	0.701690	-701	TGGCACTTCCTAGATGCCGGG	+
218943_s_at	DDX58	0.701383	-601	ATGGATTTACAGGAGTTATTT	-
207808_s_at	PROS1	0.701015	-142	ATTGACTTCCAGGTTTTGGTT	+
244675_at	RGS8	0.699635	-841	AGAAAATGTCCAGAAATGAAG	+
219508_at	GCNT3	0.699526	18	GGGCAGTTCCCGTGACTTAGA	-
233085_s_at	OBFC2A	0.699376	-599	TTAAGTATGCCTGAAATGAGG	+
205729_at	OSMR	0.698500	-40	CGCAAAGTCCGGGTTGTGAGG	+
238460_at	FAM83A	0.697861	-195	GCGCATCTCAGGGATGGCATC	+
222853_at	FLRT3	0.697671	-495	CTCTAATTCCGGCACTTTGGG	-
202934_at	HK2	0.697305	-619	CTGCAACTCCAGGAGAGGACC	+
223586_at	ARNTL2	0.696225	-47	CGGGGCTTCCCGGCGGGAAAG	-
203504_s_at	ABCA1	0.694983	-313	ATTTGCTTCCTCTAAATCCTG	-
204669_s_at	RNF24	0.694471	-99	CCGGATGCCCGGGCAGTTTCC	-
242649_x_at	C15orf21	0.694416	-352	GCTGAAATACCTGAAGTAAAA	+
203233_at	IL4R	0.694097	-17	GGGGCTTTCCTGGGCGCGGAG	-
202668_at	EFNB2	0.694017	-419	TTCCATGTCCCGGAGCACGGA	-
207610_s_at	EMR2	0.693849	-706	CCAGAATTCAAGGACTTGGTT	-
203108_at	GPRC5A	0.693132	-640	TGTGGTTTTTTGGGAGTGAAA	+
225142_at	JHDM1D	0.692724	-762	CGTTAGTTGCGGGAGGGCTGT	-

Probeset	GeneID	Score	Position	Sequence	Strand
206291_at	NTS	0.692358	-32	TGGCCATTCCCCTATATATAT	-
202269_x_at	GBP1	0.692254	-235	CTACAATTTCCTAAAATAATA	-
204642_at	S1PR1	0.692117	-608	AGGGGTTTCCCTCAGCTGAGC	-
212188_at	KCTD12	0.691168	-396	CGTGAACTTTCGGAAGCCTGT	-
203508_at	TNFRSF1B	0.690542	-369	TCTTGGTTCCCCTAAAGCAGC	+
221653_x_at	APOL2	0.689486	-294	GGGCAGAGCCTGGAATTGCAC	+
211339_s_at	ITK	0.689473	-579	TGACGTTTTGTGGAAGAGTGA	-
226757_at	IFIT2	0.688351	-307	TCACCTTAGCAGGAAGTGGGG	+
36711_at	MAFF	0.688027	-75	CGGGGCTTCCAGGGGGCGGGG	+
230563_at	RASGEF1A	0.687715	-894	TGCGGTTACCCTGCAGGGTCT	-
212942_s_at	KIAA1199	0.687476	-665	ACCCATTTTCCTGATGAGGAA	+
205199_at	CA9	0.687153	-405	GGTGGAGTCAGGGATGTATAC	+
201860_s_at	PLAT	0.687109	-619	TCGAATTTCAGGCATGTTGGC	+
218273_s_at	PDP1	0.686527	-538	CCGGGCGTCCGGGCTGTGGGG	+
209324_s_at	RGS16	0.685975	-800	GGTTAGTACCCGGTCACGGCT	-
228128_x_at	PAPPA	0.685112	-577	TGAGGTTTACCAGCAAAGTGC	+
225619_at	SLAIN1	0.684481	-327	CGCTCCTTCCCCGAGACCCTG	+
241994_at	XDH	0.684268	-504	GTCAAATGCCAGGATTTGAGT	+
218559_s_at	MAFB	0.683724	-858	GCCGGCTCCCCAGCAGTGGAG	+
229450_at	IFIT3	0.681480	-583	CTCTATTTCCATAACATGAGG	+
203765_at	GCA	0.680214	-276	TGGGGTTTCTAGAAAGAAAAG	+
210118_s_at	IL1A	0.679408	-145	TTTTAAATAGAGGAAATGAAC	-
206569_at	IL24	0.679393	-713	GTCTGTGTCCCCTGAGTGGTT	+
205476_at	CCL20	0.678936	-269	CCCTGTGACCCAGAAAAGACT	-
213094_at	GPR126	0.678846	-162	GGGGCTGTCCCGAAGGCGCAG	-
206376_at	SLC6A15	0.678786	-475	GCCGGCTTGGGGGAATAGGTA	+
214414_x_at	HBA1/HBA2	0.676396	-553	AGGGAGCTGCAGGAAGCGAGG	+
229491_at	NHEDC2	0.669293	-102	AGGAAATACAGAGAAAAGGAA	+
204298_s_at	LOX	0.667988	-142	GGGTACTTACCGTACTGGAAG	-
220030_at	STYK1	0.667523	-166	TGTAACACCCCGGCAAAGCCT	+
201631_s_at	IER3	0.667202	-120	TGGAAATTCCGACGATTAAAC	-
209183_s_at	C10orf10	0.661231	-484	CCTCATATCTGGTGAACAAGT	+
215717_s_at	FBN2	0.650986	-689	GGCCAATTGACGGGGACGGGA	+
201042_at	TGM2	0.648701	-707	ATTGGTGTCTAGGAGGTCTGT	+
235199_at	RNF125	0.634783	-944	TTTGTTTTTTGAGACATGGTC	+

[1]Genes whose expression was increase at least 4-fold in Henle-407 cells 10 h or 20 h after infection with *S.* Typhimurium revealing a predicted STAT3 binding site (V$STAT3_01, M00225, p-Value = 1.99×10^{-8}, http://159.149.160.51/pscan/) in a region -950 to +50 nucleotides around the transcription start sites.
