# Supplementary material for: Salmonella Modulation of Host Cell Gene Expression Promotes Its Intracellular Growth
Source: PLoS Pathog. 2013 Oct 3;9(10):e1003668. doi: 10.1371/journal.ppat.1003668 (PMC3789771; doi:10.1371/journal.ppat.1003668)
Supplement: Table S5 — Bacterial strains. (PDF) [file ppat.1003668.s019.pdf]

Table S5: bacterial strains

Strain	Genotype	References
SB300	wild type	1
SB136	*ΔinvA*	2
SB762	Δ*flhD*	3
SB924	*ΔsopB*	4
SB1011	*ΔsopA ΔsopB ΔsopD ΔsopE ΔsopE2 ΔavrA ΔsptP ΔslrP ΔsspH1*	5
SB1301	*ΔsopE ΔsopE2*	4
SB1302	*ΔsopE ΔsopE2 ΔsopB*	4
SB1400	*Δasd*	6
SB2176	*ΔspiA*	7

1 Kaniga, K., Bossio, J. C. & Galán, J. E. The *Salmonella typhimurium* invasion genes *invF* and *invG* encode homologues to the PulD and AraC family of proteins. *Mol. Microbiol.* **13**, 555-568 (1994).

2 Galán, J. E., Ginocchio, C. & Costeas, P. Molecular and functional characterization of the *Salmonella typhimurium* invasion gene *invA*: Homology of InvA to members of a new protein family. *J. Bacteriol.* **17**, 4338-4349 (1992).

3 Eichelberg, K. & Galan, J. E. The flagellar sigma factor FliA (sigma(28)) regulates the expression of Salmonella genes associated with the centisome 63 type III secretion system. *Infect Immun* **68**, 2735-2743 (2000).

4 Zhou, D., Chen, L. M., Hernandez, L., Shears, S. B. & Galán, J. E. A Salmonella inositol polyphosphatase acts in conjunction with other bacterial effectors to promote host cell actin cytoskeleton rearrangements and bacterial internalization. *Mol Microbiol* **39**, 248-259 (2001).

5 Hernandez, L. D., Pypaert, M., Flavell, R. A. & Galan, J. E. A Salmonella protein causes macrophage cell death by inducing autophagy. *The Journal of cell biology* **163**, 1123-1131, doi:10.1083/jcb.200309161 (2003).

6 Galán, J. E., Nakayama, K. & Curtiss, R. d. Cloning and characterization of the *asd* gene of *Salmonella typhimurium*: use in stable maintenance of recombinant plasmids in *Salmonella* vaccine strains. *Gene* **94**, 29-35 (1990).

7 Spanò, S., Xiaoyun Liu, X. & Galán, J. E. Rab29 is a Specific Cellular Determinant for the Human Pathogen Salmonella Typhi. *Nature (under review)* (2011).
