# Supplementary material for: Salmonella Modulation of Host Cell Gene Expression Promotes Its Intracellular Growth
Source: PLoS Pathog. 2013 Oct 3;9(10):e1003668. doi: 10.1371/journal.ppat.1003668 (PMC3789771; doi:10.1371/journal.ppat.1003668)
Supplement: Table S6 — Primers used in this study. (PDF) [file ppat.1003668.s020.pdf]

Table S6: primers use in this study

Gene	Forward primer	Reverse primer
ABCA1	AACTCTACATCTCCCTTCCCG	CTCCTGTCGCATGTCACTCC
AGT	TAGTCGCTGCAAAACTTGACA	ACGCCCCATAGCTCACTGT
β-actin	GAGCGCGGCTACAGCTT	TCCTTAATGTCACGCACGATTT
EGR1	ACCTGACCGCAGAGTCTTTTC	GCCAGTATAGGTGATGGGGG
FGA	CCGGACCTGGCAAGACTAC	CCCCTTTGGGTTAGTAAGTGGAG
GAPDH	CTTGAGGCTGTTGTCATACTTC	GTCCACTGGCGTCTTCAC
IL-24	CTTTGTTCTCATCGTGTCACAAC	TCCAACTGTTTGAATGCTCTCC
IL-8	TCTCAGCCCTCTTCAAAAACTTCTC	ATGACTTCCAAGCTGGCCGTGGCT
LBP	GTGTTCAGCTCTACGACCTTG	CTCTCATGTATTGGACATTGGCA
SDR16C5	TATACCTGCGATTGCAGCCAA	CGATTCCGGCATTGTTGATTAGG
SerpinB3	CGGTCTCGTGCTATCTGGAG	ATCCGAATCCTACTACAGCGG
SPINK1	AGTCTATCTGGTAACACTGGAGC	ACACGCATTCATTGGGATAAGT
STON1	CTCTCCTCCCCCATTGTAGATT	GGGAAGTCTTTGGTAGGTGTAGA
TCN1	GCCTTGATTATACTGGCTTTGGG	AGTGCCATTGTGTGCTTCCA
TNC	GCACACAGTAGATGGGGAAAA	CAGCAGCTCCTTAACATCAGG
TTP	GACTGAGCTATGTCGGACCTT	GAGTTCCGTCTTGTATTTGGGG
