# Supplementary material for: Salmonella Modulation of Host Cell Gene Expression Promotes Its Intracellular Growth
Source: PLoS Pathog. 2013 Oct 3;9(10):e1003668. doi: 10.1371/journal.ppat.1003668 (PMC3789771; doi:10.1371/journal.ppat.1003668)
Supplement: Text S1 — Heterogeneity in Salmonella Typhimurium-induced gene expression in cultured epithelial cells. (PDF) [file ppat.1003668.s021.pdf]

Supplementary Text S1:

Heterogeneity in *Salmonella* Typhimurium-induced gene expression in cultured epithelial cells

To identify cells that have potentially undergone gene expression changes as a consequence of *Salmonella* infection, we examined by immunofluorescence the presence of SerpinB3, whose expression was significantly stimulated by bacterial infection. Surprisingly, we detected SerpinB3 expression in only ~50% of infected cells (Supplementary Fig. S14A and S14B). Infection of cells with the non-replicating *S.* Typhimurium *Δasd* mutant or a mutant defective in the SPI-2 T3SS, which is required for intracellular replication (Waterman and Holden, 2003), yielded a similar number of SerpinB3 positive cells 20 h after infection (Fig. S14B). These results indicate that the heterogeneity observed in the transcriptional reprogramming is not the consequence of differences in bacterial replication. We did not observe any instances of uninfected, SerpinB3-producing cells, consistent with the hypothesis that autocrine and/or paracrine mechanisms are unlikely to be central to the *Salmonella*-induced transcriptional responses later in infection. Furthermore, we saw no evidence for the presence of cytoplasmic bacteria in either SerpinB3-positive or negative cells since the vast majority of bacteria were contained within Lamp1-positive vacuoles, indicating that the integrity of the *S.* Typhimurium vacuole was equally maintained in both types of cells (Fig. S14C). To ascertain whether this observation represented a unique feature of SerpinB3 expression or was a reflection of heterogeneity in the transcriptional response as a whole we sorted SerpinB3-expressing and non-expressing cells using FACS and examined the mRNA levels of several genes whose expression levels were shown by microarray measurements to be increased at later times after *Salmonella* infection (Fig. 1B and Table S1). We found that the levels of expression of all the examined genes were significantly higher in the SerpinB3 expressing cells than in the non-expressing cells (Fig. S14E). The mRNA levels of control genes whose expression was shown unaltered at later times of *Salmonella* infection were indistinguishable in the two cell populations (Fig. S14E). The observed heterogeneity is likely to be the result of heterogeneity in the host cell population rather than in the bacterial inoculum since the bacterial factors required for the stimulation of changes in host cell gene expression are the same as those required for bacterial entry into cells.

Reference:

Waterman, S.R., and Holden, D.W. (2003). Functions and effectors of the Salmonella pathogenicity island 2 type III secretion system. Cell Microbiol *5*, 501-511.

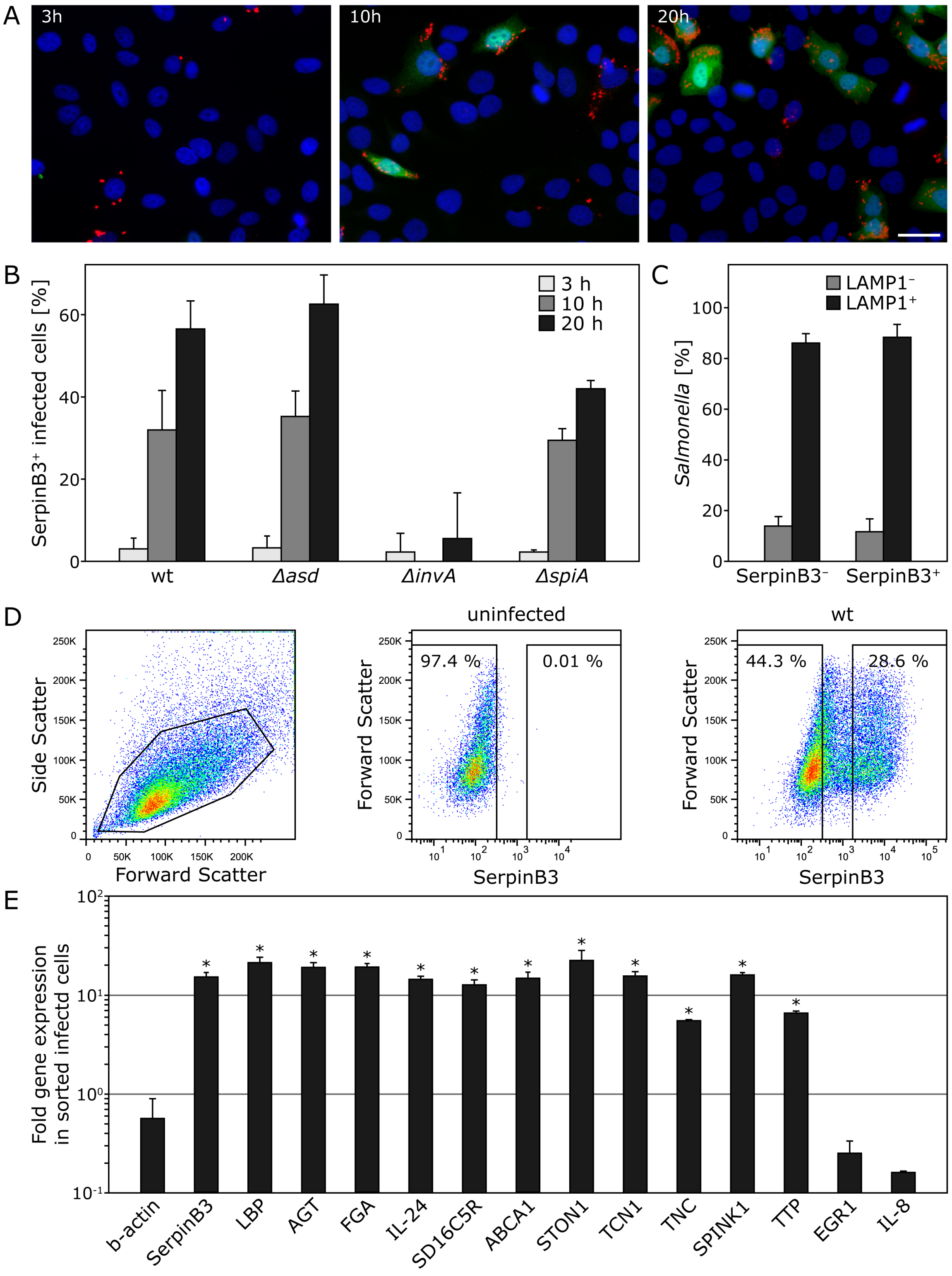

Figure S15. Heterogeneity in *Salmonella* Typhimurium-induced gene expression in cultured epithelial cells. (**A**) and (**B**) Cultured Henle-407 cells were infected (MOI = 10) with wild-type *S.* Typhimurium, the SPI-1 T3SS-defective Δ*invA,* the SPI-2-T3SS-defective Δ*spiA*, or the Δ*asd*

mutants (as indicated) for 1 h and chased for the indicated times in the presence of gentamicin. Cells were then fixed, and stained for LPS (red), SerpinB3 (green), and DNA (blue), and analyzed by epifluorescence microscopy (Bar represents 10 µm) (**A**). The number of infected cells expressing detectable levels of SerpinB3 was quantified. Values represent the means (± SD) of the percentages of *S.* Typhimurium-infected, SerpinB3-positive cells at the indicated time points and correspond to three independent experiments in which at least 100 cells per time point were analyzed (**B**). (**C**). Comparison of the localization of *S.* Typhimurium within membrane-bound vacuoles in SerpinB3-positive and SerpinB3-negative cells. Cultured Henle-407 cells were infected (MOI = 5) for 1 h with wild-type *S.* Typhimurium and chased for 20 h in gentamicin-supplemented culture medium. Cells were then fixed, stained for LPS, SerpinB3, LAMP1 and DNA and analyzed by epifluorescence microscopy. The number of *S.* Typhimurium in SerpinB3-positive or negative cells that were or were not located in LAMP1-positive vesicles was quantified. Note that the ~10% of the bacteria that were found not to be contained within LAMP1 vesicles were not necessarily located within the cell cytoplasm since LAMP1 does not label all *Salmonella*-containing vacuoles. In any case, there were no differences in the number of bacteria within LAMP1-positivie vacuoles in SerpinB3-positive or SerpinB3–negative cells. The values represent the means (± SD) of the percentages of the respective *S.* Typhimurium and correspondent to three independent experiments in which at least 175 bacteria per condition were evaluated. (**D**) FACS-sorting of SerpinB3-positive and negative cells. Henle-407 cells were infected (MOI = 30) with wild-type *S.* Typhimurium (wt) for 1 h, and 20 h post infection cells were fixed and immuno stained for SerpinB3. SerpinB3-positive and negative cells were separated by FACS sorting. In each case, four million cells were sorted. Depicted are the gated cells as well as SerpinB3-positive and SerpinB3-negative infected cells. (**E**) Relative expression of a selected group of genes in SerpinB3-positive cells. Cultured Henle-407 cells were infected (MOI = 30) with wild-type *S.* Typhimurium and 20 h post infection cells were fixed, stained for SerpinB3, and FACS-sorted. RNA was isolated from SerpinB3 positive and negative populations and expression levels of the indicated genes were determined by qRT-PCR. Values were normalized to those of GAPDH and represent the means (± SEM) of the fold differences in gene expression between SerpinB3-positive and SerpinB3-negative cells of three independent experiments. *: indicates statistically significant differences with control values ($p \leq 0.03$).
